# Development of ^18^F-Labeled Radiotracers for PET Imaging of the Adenosine A_2A_ Receptor: Synthesis, Radiolabeling and Preliminary Biological Evaluation

**DOI:** 10.3390/ijms22052285

**Published:** 2021-02-25

**Authors:** Thu Hang Lai, Susann Schröder, Magali Toussaint, Sladjana Dukić-Stefanović, Mathias Kranz, Friedrich-Alexander Ludwig, Steffen Fischer, Jörg Steinbach, Winnie Deuther-Conrad, Peter Brust, Rareş-Petru Moldovan

**Affiliations:** 1 Helmholtz-Zentrum Dresden-Rossendorf (HZDR), Department of Neuroradiopharmaceuticals, Institute of Radiopharmaceutical Cancer Research, Research Site Leipzig, 04318 Leipzig, Germany; m.toussaint@hzdr.de (M.T.); s.dukic-stefanovic@hzdr.de (S.D.-S.); mathias.kranz@uit.no (M.K.); f.ludwig@hzdr.de (F.-A.L.); s.fischer@hzdr.de (S.F.); steinbach-joerg@web.de (J.S.); w.deuther-conrad@hzdr.de (W.D.-C.); p.brust@hzdr.de (P.B.); 2 Department of Research and Development, ROTOP Pharmaka Ltd., Dresden 01328, Germany; s.schroeder@hzdr.de; 3 PET Imaging Center, University Hospital of North Norway (UNN), 9009 Tromsø, Norway; 4 Nuclear Medicine and Radiation Biology Research Group, The Arctic University of Norway, 9009 Tromsø, Norway

**Keywords:** adenosine receptors, A_2A_ receptor, fluorine-18, positron emission tomography, vipadenant

## Abstract

The adenosine A_2A_ receptor (A_2A_R) represents a potential therapeutic target for neurodegenerative diseases. Aiming at the development of a positron emission tomography (PET) radiotracer to monitor changes of receptor density and/or occupancy during the A_2A_R-tailored therapy, we designed a library of fluorinated analogs based on a recently published lead compound (**PPY**). Among those, the highly affine 4-fluorobenzyl derivate (**PPY1**; *K*_i_(*h*A_2A_R) = 5.3 nM) and the 2-fluorobenzyl derivate (**PPY2**; *K*_i_(*h*A_2A_R) = 2.1 nM) were chosen for ^18^F-labeling via an alcohol-enhanced copper-mediated procedure starting from the corresponding boronic acid pinacol ester precursors. Investigations of the metabolic stability of [^18^F]**PPY1** and [18F]**PPY2** in CD-1 mice by radio-HPLC analysis revealed parent fractions of more than 76% of total activity in the brain. Specific binding of [^18^F]**PPY2** on mice brain slices was demonstrated by in vitro autoradiography. In vivo PET/magnetic resonance imaging (MRI) studies in CD-1 mice revealed a reasonable high initial brain uptake for both radiotracers, followed by a fast clearance.

## 1. Introduction

Adenosine is an endogenous ubiquitous purine nucleoside formed by the hydrolysis of extracellular 5′-adenosine triphosphate (ATP) by ectonucleotidases (CD39, CD73) and degraded by phosphorylation to adenosine monophosphate or by deamination to inosine [1,2]. In addition, the extracellular levels of adenosine are regulated by equilibrative and concentrative nucleoside transporters expressed at the plasma membranes of a great variety of cells [3]. It acts as a signaling molecule, which binds to four subtypes of purinergic P1 receptors, namely A_1_R, A_2A_R, A_2B_R and A_3_R, which are coupled to different G protein-mediated intracellular pathways [4,5]. In this way, adenosine regulates energy homeostasis and affects the function of various organs and tissues such as brain, heart, brown adipose tissues and others. Targeted drug interaction with the specific receptor subtypes, metabolizing enzymes or transporters are regarded to have important therapeutic potential.

The function of adenosine in the central nervous system (CNS) has extensively been studied. It regulates the release and uptake of neurotransmitters, modulates synaptic plasticity and protects from ischemic, hypoxic and oxidative stress [6,7]. One of its major targets in the CNS, the A_2A_R, is highly expressed in the striatum, where it is involved in the indirect basal ganglia pathway [8]. Lower densities of the A_2A_R were reported in GABAergic medium spiny neurons post-synapses [9], astrocytes [10], microglia [11], oligodendrocytes [12] and endothelial cells [13]. The neuromodulatory role of the A_2A_R in the CNS is strongly related to its ability to interact with other receptors forming heteromers, such as dopamine D_2_R-A_2A_R [14], glutamate mGluR_5_R-A_2A_R [15], cannabinoid CB_1_R-A_2A_R [16] and adenosine A_1_R-A_2A_R [17].

A_2A_R ligands have been investigated in neurodegenerative diseases, such as Huntington’s disease (HD), Alzheimer’s disease (AD) and Parkinson’s disease (PD) [7,18,19,20]. HD is defined by the loss of medium spiny neurons [21] and several studies have demonstrated a marked loss of striatal A_2A_R [22]. In transgenic animal models of HD, A_2A_R agonists have shown to reverse motor deficits, whereas A_2A_R antagonists worsen motor performance [23,24,25]. Imaging of the A_2A_R with selective positron emission tomography (PET) radiotracers is regarded as a useful tool to advance our understanding of the functional role of this receptor in healthy and diseased brains [20]. The ^11^C-labeled PET radiotracer [^11^C]**KF18446** has been used to demonstrate the reduced A_2A_R expression in an animal model of HD [26]. This study showed a significant reduced binding potential of [^11^C]**KF18446** in the quinolinic acid-lesioned striatum. AD is a progressive disease characterized by the loss of cholinergic neurons and changes in the protein structure. Disturbances in the protein folding result in the formation and aggregation of β-amyloid (Aβ), which is resistant to the enzymatic proteolysis [27]. In AD, the A_2A_R is upregulated in the frontal cortex and hippocampus [28]. In vivo experiments in animal models of AD revealed that A_2A_R antagonists prevent the neurotoxicity and synaptotoxicity of Aβ [29] as well as enhance memory function [30]. So far, no PET imaging of the A_2A_R has been performed in HD and AD patients. Thus, in vivo imaging of the A_2A_R might increase the understanding of the disease pathogenesis and enable the development of emerging therapies.

**Figure 1:**
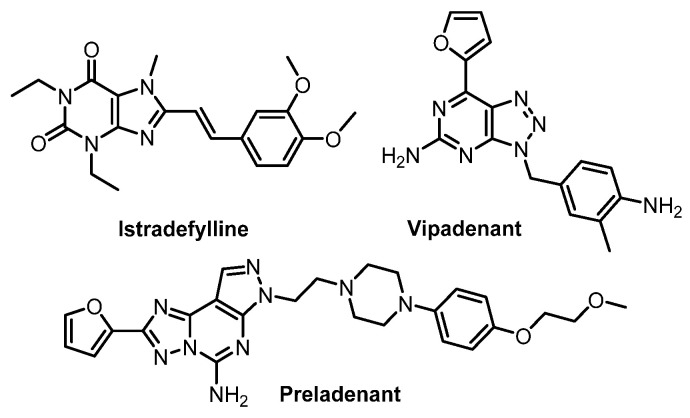
Representative A_2A_R antagonists in clinical trials [36].

In contrast to HD and AD, the A_2A_R expression has been intensively studied in PD patients. The most established therapy of PD is based on the administration of L-3,4-dihydroxyphenylalanine (L-DOPA) in combination with inhibitors to decrease the metabolism of dopamine. Although L-DOPA therapy has increased the life quality of patients with PD by reducing motoric disorders, long-term treatment with L-DOPA often leads to a decreased efficacy over time accompanied with pronounced adverse effects, such as dyskinesia, “on” phase shortening and psychotic syndromes [31]. Results from clinical II and III trials demonstrated that the adjunctive treatment with A_2A_R antagonists improves the mobility of PD patients as well as reduces adverse effects of long-term L-DOPA treatment [32,33]. One of the most investigated A_2A_R antagonists is **istradefylline** (former KW-6002; Nourianz^®^, Kyowa Kirin Inc., Bedminster, NJ, USA), which was approved by the U.S. Food and Drug Administration (FDA) in April 2019 for adjunctive treatment in patients with PD [34,35]. Several other A_2A_R antagonists, such as **vipadenant** and **preladenant** (Figure 1), have also been studied in clinical trials for treatment of neurodegenerative diseases, but so far, none of them has been approved by the FDA [36]. Furthermore, some findings suggest that A_2A_R antagonists might also be efficacious as monotherapeutic drug in PD patients at an early stage of the disease [36]. The early clinical diagnosis of PD is highly challenging which reinforces the importance of the functional imaging targeting the pathophysiology of the disease progress. PET imaging of the A_2A_R would be an effective technique to monitor the disease progression, to assess dose-dependent occupancy of the receptor population by therapeutic drugs and to evaluate the efficacy of the therapy.

**Figure 2:**
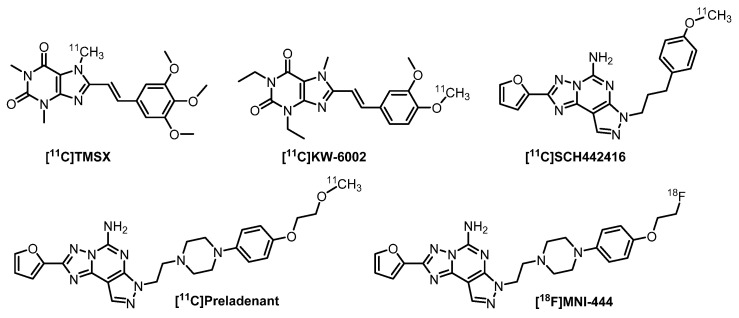
A_2A_R PET radiotracers in clinical trials [18].

Five radiotracers for PET imaging of the A_2A_R, namely [^11^C]**TMSX**, [^11^C]**preladenant**, [^11^C]**SCH442416**, [^11^C]**KW-6002** and [^18^F]**MNI-444**, have been evaluated in humans (Figure 2) [18]. Among those, [^11^C]**SCH442416** is the only radiotracer investigated in PD patients with L-DOPA-induced dyskinesia despite suffering from high levels of background non-specific binding [37]. Furthermore, fluorine-18 is the most predominant radionuclide for PET imaging due to its attractive half-life (109.7 min) that allows multistep synthesis and distribution to various PET centers after production, and its lower positron energy (635 keV) facilitating high image resolution. Thus, an ^18^F-labeled radiotracer, namely [^18^F]**MNI-444**, was developed for clinical application. Although [^18^F]**MNI-444** presents an excellent ratio of specific-to-non-specific binding in healthy subjects, the relative slow kinetics of this radiotracer may limit its routine clinical use due to too-long scan times needed [38].

**Figure 3:**
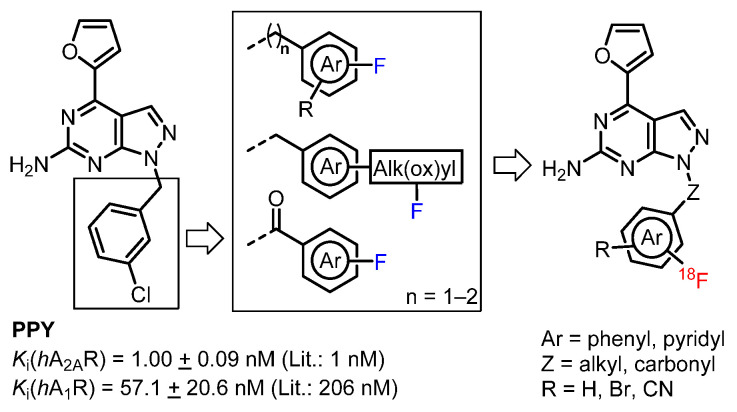
Lead compound **PPY** and illustration of approached strategies towards novel ^18^F-labeled derivatives.

Aiming to develop a new A_2A_R radiotracer presenting the advantages of fluorine-18, a high specific binding and suitable pharmacokinetic for clinical use, we selected **PPY** (Figure 3) [39,40], an analog of the extensively studied A_2A_R antagonist **vipadenant** (Figure 1), as lead compound due to its high A_2A_R binding affinity of 1 nM and 260-fold selectivity over the A_1_R subtype. Furthermore, it possesses all favorable physicochemical and absorption, distribution, metabolism, and excretion (ADME) properties for a CNS PET radiotracer, e.g., M = 326 g/mol, c*LogD*_7.4_ = 4.01, polar surface area = 83 Å^2^ and H-bond donors = 2 [41]. The 4-(furan-2-yl)pyrazolo-[3,4*-d*]pyrimidine building block was used as starting point for further investigations. Fluorinated derivatives have been synthesized by varying the substituents at the *N*1 position of the pyrazole. Hence, the lead compound was modified by replacing the benzyl moiety by various substituted fluorobenzyl, fluorobenzoyl or fluoropyridyl groups and linker extensions to study the impact on the binding affinity towards the A_2A_R (Figure 2). Thus, a series of 20 novel A_2A_R ligands bearing the fluorine atom either at aromatic or aliphatic positions was developed. The ligands with the highest binding affinity and off-target selectivity were selected for the development of ^18^F-labeled ligands by radiofluorination of the corresponding precursors and for preliminary biological investigations of the novel potential A_2A_R radiotracers.

## 2. Results

### 2.1. Chemistry

**Scheme 1:**
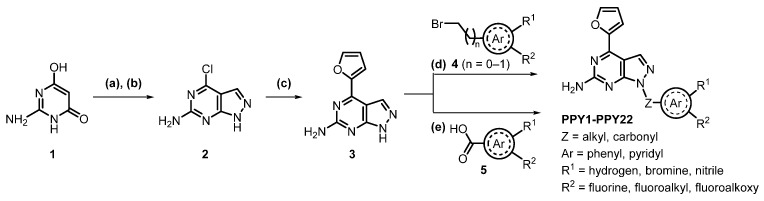
Synthesis of novel fluorinated **PPY** derivatives, reagents and conditions: (**a**) DMF, POCl_3_, 0–100 °C, 5 h; (**b**) N_2_H_4_, H_2_O, NEt_3_, RT, 3 h; (**c**) tributyl(furan-2-yl)stannane, PdCl_2_(PPh_3_)_2_, DMF, 90 °C, 18 h; (**d**) (bromomethyl)aryl **4** (n = 0–1), K_2_CO_3_, DMF, RT, 6 h; (**e**) benzoic acid **5**, BOP, NEt_3_, CH_2_Cl_2_, RT, 18 h.

The synthesis of the novel fluorinated derivatives has been performed as described for **PPY [39,40]** with minor modifications. First, commercially available pyrimidine **1** is formylated via a Vilsmeier–Haack reaction. Then, an electrophilic aromatic substitution led to an iminium ion, which was hydrolyzed during workup to the corresponding aldehyde. After cyclization with hydrazine and purification by recrystallization from *N*,*N*-dimethylformamide (DMF)/water, compound **2** was isolated with 70% yield over two steps. In the literature, the pyrazolo[3,4-*d*]pyrimidine **3** was obtained by protection of the amine in **2** with a *tert*-butoxycarbonyl (Boc) group, followed by Stille coupling with 2-(tributyl-stannyl)furan in the presence of bis(triphenylphosphine)-palladium(II) and, finally, thermal Boc-group removal using aqueous dimethylamine with an overall yield of 16% [39,40]. In our hands, the Stille coupling was optimized by using the unprotected compound **2** directly to give **3** with 82% yield. The key building block **3** was used for the development of all further derivatives described herein as shown in Scheme 1.

**Scheme 2:**
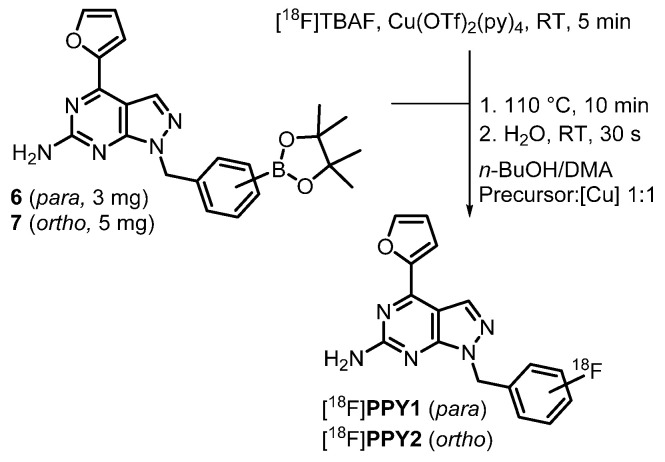
Radiosynthesis of [^18^F]**PPY1** and [^18^F]**PPY2** by an alcohol-enhanced copper-mediated radiofluorination of the corresponding boronic acid pinacol ester precursor **6** or **7**.

The fluorinated target products were synthesized by either a benzylation or a coupling reaction of **3** with the corresponding benzoic acids (Scheme 1). The (bromomethyl)pyridine and (bromomethyl)benzene derivatives **4**, used for the synthesis of compounds **PPY1-PPY19** and **PPY22**, were either purchased or synthesized via a Wohl-Ziegler reaction with *N*-bromosuccinimide (NBS) and 2,2′-azobis(2-methylpropionitrile) (AIBN) as radical initiator in carbon tetrachloride. The regioselective benzylation with the corresponding (bromomethyl)aryls was modified from literature by using potassium carbonate instead of sodium hydride [39,40]. Under these conditions, 6 h instead of 1 h were necessary for complete conversion, but fewer by-products were formed and higher yields could be achieved (82% vs. 16% [39]). The amide coupling of compound **3** with 2- or 4-fluorobenzoic acid **5** was performed in the presence of benzotriazol-1-yloxy-tris(dimethylamino)phosphonium hexafluorophosphate (BOP) as coupling reagent. Analogous to the fluorinated derivatives, the boronic acid pinacol ester precursors **6** and **7** (Scheme 2) were synthesized by a benzylation reaction of **3** with the corresponding (bromomethyl)phenyl moieties.

### 2.2. Structure–Activity Relationship (SAR) Studies

**Figure 4:**
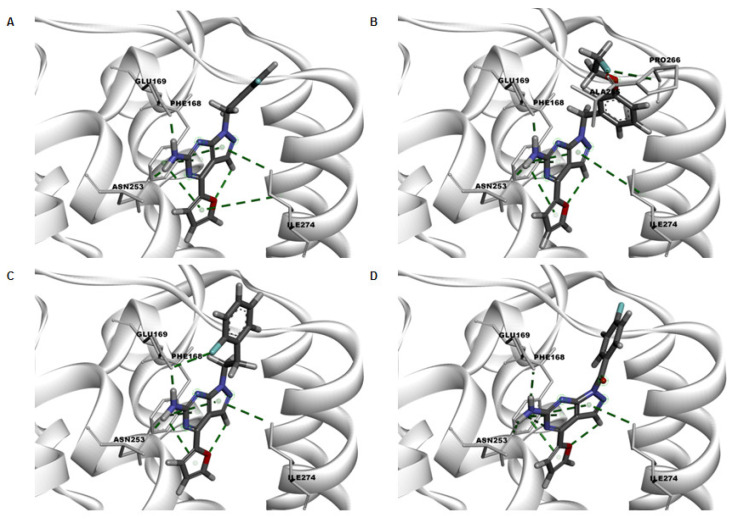
Key interactions in green dotted lines of the docking poses of representative active compounds (**A**) **PPY2**, (**B**) **PPY17**, (**C**) **PPY19** and (**D**) **PPY21** in the binding site of crystal structure of A_2A_R (PDB ID: 4EIY).

Preliminary quantitative structure-activity relationship (QSAR) modelling studies were carried out to predict binding poses and intermolecular interactions responsible for determined binding affinities (*K*_i_) by radioligand binding assays (Figure 4). The X-ray crystallographic chimeric protein structure of A_2A_R-BRIL in complex with the antagonist **ZM241385** (PDB ID: 4EIY) was selected for QSAR modelling due to the highest resolution (1.8 Å) of all available non-thermostabilized structures [42]. The pyrazolo[3,4-*d*]pyrimidine unit of the herein developed compounds is located in the binding cavity and is stabilized by aromatic π-π interaction with Phe168. Furthermore, the amine forms hydrogen bonds with Glu169 and Asn253. The benzyl and pyridyl groups of the fluorinated ligands **PPY1**-**PPY7** are additionally stabilized by π-π interaction with Tyr271. The decreased binding affinity of **PPY19** can be explained by the missing interaction with Tyr271 due to the linker extension resulting in another orientation of the benzyl group in the binding pocket. The same orientation change is obtained by the introduction of a carbonyl group as linker for ligands **PPY20** and **PPY21**. The high binding affinity of **PPY17** can be explained by additional interactions of the fluorine atom with His264, Ala265, Pro266 and Leu267. Despite the promising binding properties of **PPY17**, this compound was not selected for development of the corresponding radiotracer due to the predicted radiometabolites resulting from enzymatic *N*-debenzylation and *O*-dealkylation [43,44,45].

The structures of the final products prepared in this study along with determined binding affinities towards the A_2A_R and A_1_R subtypes are given in Table 1. Exchanging the halogen of the lead compound from chlorine (**PPY**) to fluorine (**PPY3**) resulted in a 3-fold decreased A_2A_R binding affinity. Thus, it could be assumed, that chlorine is involved in halogen bonding in the binding pocket of the A_2A_R. In contrast to chlorine, fluorine is not able to form halogen bonding interactions due to its higher electronegativity and the lack of polarizability [46]. Further investigations were based on the impact of fluorine in *ortho-*, *meta-* and *para-*position of the benzyl ring on the binding potency. The fluorination in *ortho*-position (**PPY2**) led to a 2.5-fold increased A_2A_R binding affinity compared to the *para-*position (**PPY1**). Due to our continued interest in nucleophilic aromatic radiofluorination, the reactivity of the ^18^F-labeling position has to be considered. Consequently, fluorinated pyridyl groups were established in the lead compound. In general, no further electron withdrawing substituents are needed for the radiolabeling of pyridines at the 2- and 4-positions due to the considerably low electron density, which facilitate the nucleophilic attack of [^18^F]fluoride [47,48]. However, the incorporation of heteroatoms in 2-position to fluorine (**PPY4**-**PPY7**) led to a decrease in the A_2A_R binding affinity. Subsequently, the tolerability of further substituents was investigated regarding the binding potency. For that purpose, the impact of substituents at the phenyl ring was explored by introduction of bromine (**PPY8**-**PPY11**) or a nitrile group (**PPY12**-**PPY15**) in *meta*-position to fluorine. In comparison with the monosubstituted **PPY2**, the bromine in compound **PPY8** led to a 2-fold increased A_2A_R binding affinity. Furthermore, a trend of A_2A_R binding potencies regarding the fluorine position in the benzyl ring can be ranked as follows: *ortho* (**PPY2**, **PPY8**, **PPY11**, **PPY12**, **PPY15**) > *meta* (**PPY3**, **PPY10**, **PPY14**) > *para* (**PPY1**, **PPY9**, **PPY13**). Based on the lead compound, elaboration of the *meta*-substituent by introduction of aliphatic fluorine was also investigated (**PPY16**-**PPY18**). The 3-(2-fluoroethyl)benzyl derivative **PPY16** exhibited increased A_2A_R affinity and only slightly decreased selectivity. Replacing of the fluoroethyl group by a fluoroethoxy group (**PPY17**) increased the A_1_R selectivity. Furthermore, extension of the linker between the pyrazole and the phenyl ring with one methylene group (**PPY19**) was detrimental for the binding potency towards the A_2A_R. *N*-acyl substitution was also tested by exchanging the methylene group by a carbonyl group (**PPY20**, **PPY21**). However, this modification led to a reduction of the A_2A_R binding affinity.

Among our derivatives, the most potent A_2A_R ligands based on the pyrazolo[3,4-*d*]pyrimidine scaffold are derivatives substituted with 4-fluorobenzyl (**PPY1**) and 2-fluorobenzyl (**PPY2**). These compounds were herein selected for radiofluorination to investigate the ability of this scaffold to cross the blood-brain barrier (BBB) and to assess its in vitro and in vivo profiles.

### 2.3. Radiochemistry

**Figure 5:**
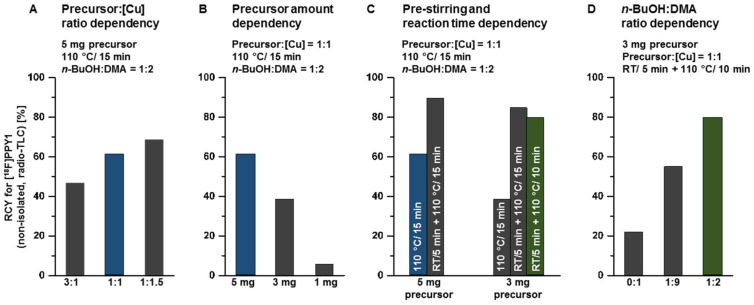
Investigated reaction conditions for the radiosynthesis of [^18^F]**PPY1**: (**A**) ratio of precursor **6**-to-copper catalyst, (**B**) amount of precursor **6** and (**C**) with/without pre-stirring of [^18^F]TBAF with [Cu] for 5 min at RT and reaction time dependency, (**D**) ratio of *n*-BuOH-to-DMA.

The radiosynthesis of [^18^F]**PPY1** and [^18^F]**PPY2** (Scheme 2) is based on a copper-mediated procedure established by the groups of P. J. Scott and V. Gouverneur [49,50,51,52,53] and further developed by several other groups [54,55]. In the present study, an alcohol-enhanced copper-mediated radiofluorination of the boronic acid pinacol ester precursor **6** was performed for the radiosynthesis of [^18^F]**PPY1**. The reaction conditions were systematically optimized by varying the ratio of precursor-to-copper catalyst, the amount of precursor, the solvent ratio of *n*-butanol (*n*-BuOH) and *N,N*-dimethylacetamide (DMA), the reaction time and pre-stirring of the tetra-*n*-butylammonium [^18^F]fluoride complex ([^18^F]TBAF) with the copper catalyst (Figure 5).

First, the dependency of the radiochemical yield on the ratio of precursor-to-tetrakis- (pyridine)copper(II) triflate (Cu(py)_4_(OTf)_2_, further on abbreviated as [Cu]) was investigated. The amount of both reactants was adjusted to simplify the purification step as well as to comply with the guidance of the International Council for Harmonisation of Technical Requirements for Pharmaceuticals for Human Use on elemental impurities (ICH Q3D). In this sense, the reaction was performed by nucleophilic substitution of the boronic acid pinacol ester **6** by [^18^F]fluoride using [^18^F]TBAF and [Cu] (5 mg vs. 3 mg vs. 1 mg, dissolved in 400 µL *n*-BuOH). Radio-TLC analysis from aliquots of the reaction mixture revealed radiochemical yields of 47%, 61% and 69% for precursor-to-[Cu] ratios of 3:1, 1:1 and 1:1.5, respectively. All further experiments were performed with a ratio of 1:1 of precursor-to-copper catalyst due to the toxicity of copper and to prevent overloading of the HPLC column during purification. Afterwards, the amount of precursor **6** was systematically reduced to simplify the isolation of [^18^F]**PPY1** by semi-preparative reversed-phase HPLC (RP-HPLC). As expected, this resulted in a decrease in the achievable radiochemical yield from 61% (5 mg) to 39% (3 mg) and 6% (1 mg) of [^18^F]**PPY1** (non-isolated, radio-TLC).

Furthermore, the effect of pre-stirring of the copper catalyst and the [^18^F]TBAF complex on the radiochemical yield was investigated. The proposed mechanism is based on the Chan-Evans-Lam coupling reaction (see Supporting information, Figure S1) and involves the transmetalation of a [Cu(II)(OTf)^18^F] complex and subsequent oxidation by air or another equivalent of Cu(II) triflate to generate the highly reactive Cu(III) species [56]. Afterwards, the high-valent Cu(III) complex is able to form facile C-F bonds by reductive elimination. The relatively mild conditions employed in the reaction also indicate that the C-F coupling has a low activation barrier. It is hypothesized that the initial formation of the [Cu(II)(OTf)^18^F)] complex might be capable of facilitating the following transmetalation step [57]. This hypothesis was investigated by pre-stirring of the [^18^F]TBAF complex as fluoride source and [Cu] as catalyst at room temperature (RT) for 5 min followed by addition of either 5 mg or 3 mg of the precursor **6** and then, thermal heating of the reaction mixture at 110 °C for 15 min. This approach resulted in a significant increase in the radiochemical yield of non-isolated [^18^F]**PPY1** to 90% (5 mg, radio-TLC) and 85% (3 mg, radio-TLC). All further experiments were performed with 3 mg of precursor **6** due to the high radiochemical yield of [^18^F]**PPY1** and the advantages for purification of the radiotracer by semi-preparative RP-HPLC as mentioned above. In addition, the reaction time of the ^18^F-labeling step could be reduced from 15 to 10 min without significant impact on the radiochemical yield (85% vs. 79%, radio-TLC).

The previous experiments for the optimization of the reaction conditions have been performed in a solvent mixture of *n*-BuOH/DMA (1:2, *v/v*). The efficient application of alcohols, especially *n*-BuOH as co-solvent for copper-mediated radiofluorination, was adapted from literature [55]. The use of only DMA or a mixture of *n-*BuOH/DMA (1:9, *v/v*) as solvent resulted in a reduction of the radiochemical yield to either 22% or 55% of [^18^F]**PPY1** (non-isolated, radio-TLC), respectively.

**Figure 6:**
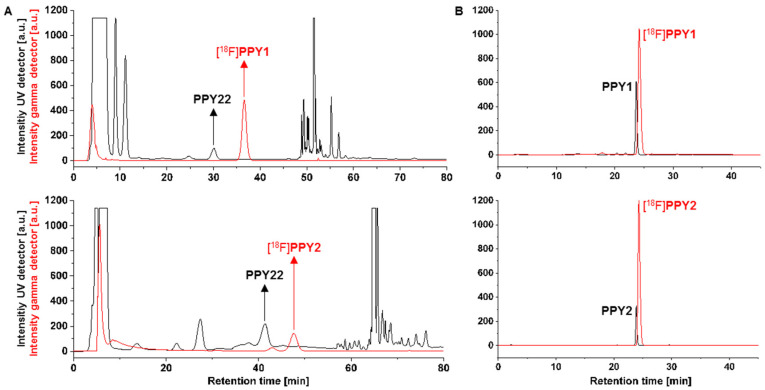
(**A**) Representative radio- and UV-chromatograms obtained for isolation of [^18^F]**PPY1** [ReproSil-GOLD 120 C18 column (250 × 10 mm), 34% MeCN/H_2_O, flow rate: 4 mL/min] and [^18^F]**PPY2** [ReproSil-Pur 120 C18-AQ column (250 × 10 mm), 30% MeCN/H_2_O, flow rate: 5 mL/min] by semi-preparative RP-HPLC (**PPY22** = by-product); (**B**) radio- and UV-chromatograms of formulated [^18^F]**PPY1** and [^18^F]**PPY2** co-injected with the corresponding reference compound **PPY1** or **PPY2** [ReproSil-GOLD 120 C18 column (250 × 4.6 mm), 10–90–10% MeCN/H_2_O, flow rate: 1 mL/min].

The optimized procedure for the radiosynthesis of [^18^F]**PPY1** is summarized in Scheme 2. In brief, [Cu] in DMA is added to the anhydrous [^18^F]TBAF complex in *n*-BuOH and the mixture is stirred at RT for 5 min. Afterwards, precursor **6** in DMA is added and the reaction mixture is stirred at 110 °C for 10 min. The reaction was quenched by the addition of 1 mL water to obtain [^18^F]**PPY1** with a radiochemical yield of 78 ± 7% (non-isolated, n = 6, radio-TLC). [^18^F]**PPY1** was isolated via semi-preparative RP-HPLC (Figure 6A) with a retention time of about 36 min, which was required to separate the radiotracer from the highly A_2A_R affine by-product **PPY22** (Table 1) produced by protodeboronation of the precursor **6** and identified by HPLC analysis of the reaction mixture co-eluted with **PPY22**. The final purification and concentration proceeded smoothly by loading the isolated fractions on a pre-conditioned RP-SPE cartridge and subsequent elution of [^18^F]**PPY1** with absolute ethanol. For biological investigation, the ethanolic solution containing [^18^F]**PPY1** was reduced under a gentle argon stream at 70 °C, DMSO was added for better solubility and [^18^F]**PPY1** was finally formulated in isotonic saline containing < 10% of both EtOH and DMSO (*v/v*), respectively. Starting with activities ranging from 1–3 GBq, [^18^F]**PPY1** was successfully obtained with a radiochemical yield of 52 ± 7% (n = 5, end of bombardment = EOB), a high radiochemical purity (≥99%) and molar activities in the range of 90–227 GBq/µmol (end of synthesis = EOS) in a total synthesis time of about 148 ± 8 min. Radio-HPLC analysis of the final product under co-elution with the corresponding reference compound **PPY1** confirmed the identity of the radiotracer (Figure 6B).

The radiosynthesis of [^18^F]**PPY2** was performed by an alcohol-enhanced copper-mediated radiofluorination of boronic acid pinacol ester **7** as optimized for the radiosynthesis of [^18^F]**PPY1** with minor modifications (Scheme 2). The amount of precursor and the reaction time had to be increased due to the low radiochemical yield of [^18^F]**PPY2,** which is likely to be caused by the steric hinderance at the *ortho*-position (3 mg/10 min: 8%, 5 mg/ 15 min: 12%, radio-TLC). Furthermore, the amount of *n-*BuOH was decreased to a *n*-BuOH-to-DMA ratio of 1:4 to prevent distortion of the peak shape during isolation of the radiotracer by semi-preparative RP-HPLC (Figure 6A). Starting with activities ranging from 2–4 GBq, [^18^F]**PPY2** was successfully obtained with a radiochemical yield of 12 ± 4% (n = 3, EOB), a high radiochemical purity (≥ 99%) and molar activities in the range of 50–80 GBq/µmol (EOS) in a total synthesis time of about 147 ± 17 min. Further attempts to increase the yield were not performed since [^18^F]**PPY2** was obtained in sufficiently high activity for biological investigation. Radio-HPLC analysis of the final product under co-elution with the corresponding reference compound **PPY2** confirmed the identity of the radiotracer (Figure 6B).

Stability of [^18^F]**PPY1** and [^18^F]**PPY2** was proven in all tested media (saline, PBS and *n*-octanol) at 37 °C up to 60 min. The distribution coefficient (Log*D*_7.4_) in the *n*-octanol-PBS system was experimentally determined by the shake-flask method. Observing Log*D*_7.4_ values of 1.59 ± 0.15 (n = 3) and 2.01 ± 0.16 (n = 3) for [^18^F]**PPY1** and [^18^F]**PPY2**, respectively, we assume that a significant passive diffusion through the BBB can be expected for both radiotracers [41,58,59].

### 2.4. Metabolite Analysis

**Figure 7:**
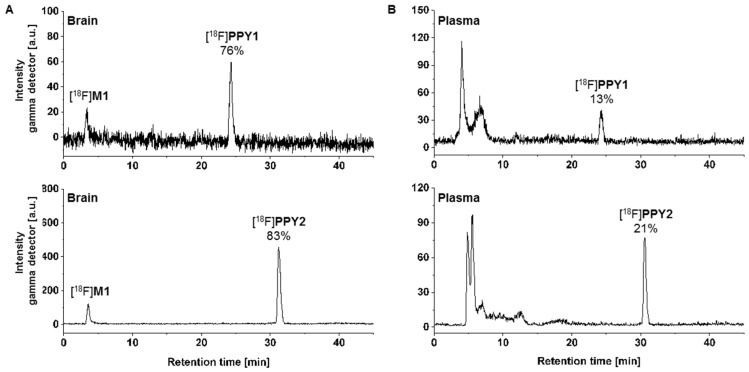
In vivo metabolism study in CD-1 mice: Representative radio-chromatograms of extracted (**A**) brain and (**B**) plasma samples at 30 min p.i. of [^18^F]**PPY1** [ReproSil-GOLD 120 C18 column (250 × 4.6 mm, 5 µm), 10-90-10% MeCN/H_2_O, flow rate: 1 mL/min] and [^18^F]**PPY2** [ReproSil-Pur 120 C18-AQ column (250 × 4.6 mm, 5 µm) 10–90–10% MeCN/H_2_O, flow rate: 1 mL/min].

**Scheme 3:**
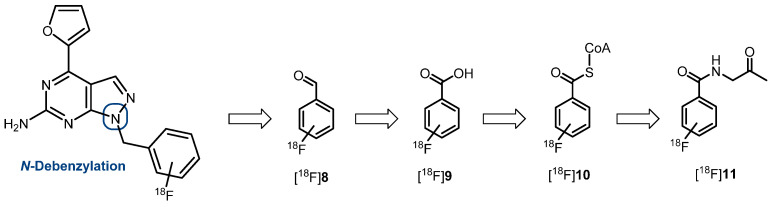
Possible radiometabolites of [^18^F]**PPY1** and [^18^F]**PPY2** and suggested metabolic pathway from [^18^F]fluorobenzaldehyde [^18^F]**8** to [^18^F]fluorobenzoglycine [^18^F]**11** [43,58].

Radiometabolite analysis of [^18^F]**PPY1** and [^18^F]**PPY2** was performed for plasma and brain at 30 min after radiotracer injection into CD-1 mice (n = 2, respectively). Recovery efficiencies of extracted activity were 82–93% and 95–98%, for plasma and brain homogenates, respectively. A high amount of radiometabolites was detected in plasma with only 13% and 21% of total activity corresponding to intact [^18^F]**PPY1** and [^18^F]**PPY2** (Figure 7B). In brain samples, 76% and 83% of total activity represented intact [^18^F]**PPY1** and [^18^F]**PPY2** (Figure 7A). One major hydrophilic metabolite ([^18^F]**M1**, t_R_ = 3 min) was detected in brain, which might be a result of *N*-debenzylation as previously reported in an in vivo study of **vipadenant [43]**. The possible pathway could be explained by the metabolic degradation of the ^18^F-bearing benzyl group and formation of [^18^F]fluorobenzaldehyde [^18^F]**8**, that is converted to [^18^F]fluorobenzoic acid [^18^F]**9**, which is then metabolized to [^18^F]fluorobenzoyl-CoA [^18^F]**10** and [^18^F]fluorobenzoglycine [^18^F]**11** (Scheme 3). It is known that [^18^F]**9** and [^18^F]**10** are able to cross the BBB and might limit the applicability of [^18^F]**PPY1** and [^18^F]**PPY2** for PET imaging and quantification of the A_2A_R in the brain.

### 2.5. Autoradiography Study

**Figure 8:**
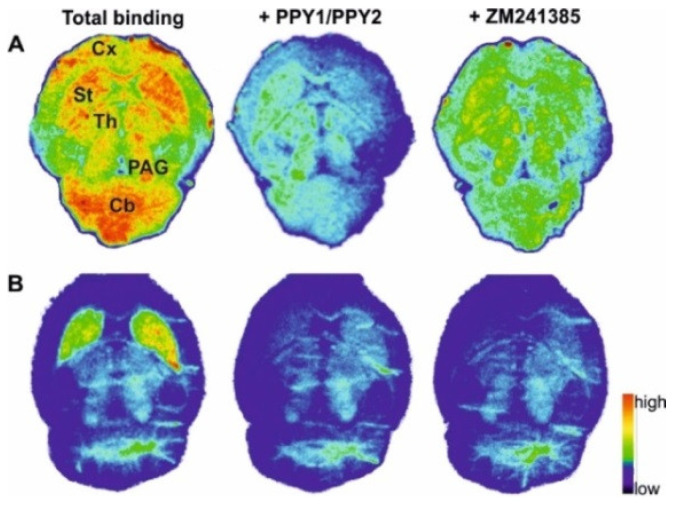
Representative autoradiographic images of the transversal plane of mouse brain slices representing total binding of (**A**) [^18^F]**PPY1** and (**B**) [^18^F]**PPY2**. Non-specific binding is depicted in the presence of either 1 µM **PPY1**, **PPY2** or **ZM241385**. Abbreviations: Cb—cerebellum; Cx—cortex; PAG—periaqueductal gray; St—striatum; Th—thalamus.

The in vitro autoradiography studies were performed to determine the distribution of [^18^F]**PPY1** and [^18^F]**PPY2** in mouse brain (Figure 8). For [^18^F]**PPY1**, displaceable binding was detected in various brain regions. The radiotracer binds to the striatum, an A_2A_R-rich region, as well as to A_2A_R-poor regions, such as cerebellum, periaqueductal gray, thalamus and cerebral cortex. In contrast to [^18^F]**PPY1**, the highest binding of [^18^F]**PPY2** was observed in the striatum. Competition experiments with the selected A_2A_R antagonist **ZM241385** revealed that 42% and 62% of total binding of [^18^F]**PPY1** and [^18^F]**PPY2**, respectively, could be displaced in striatum. A maximum receptor density (*B*_max_) value of 430 fmol/mg wet weight in mouse striatum was described by using [^3^H]**ZM241385** [59]. As a general rule, the *K*_d_ value of a PET radiotracer should be 5–10 times lower than the *B*_max_ value and, thus, the affinity of [^18^F]**PPY1** and [^18^F]**PPY2** might probably be sufficient to clearly visualize the A_2A_R in the brain [60]. The non-specific binding of [^18^F]**PPY1** as well as [^18^F]**PPY2** is presumably not related to significant binding to the A_1_R (Table 1), but to other off-targets.

### 2.6. PET Studies

**Figure 9:**
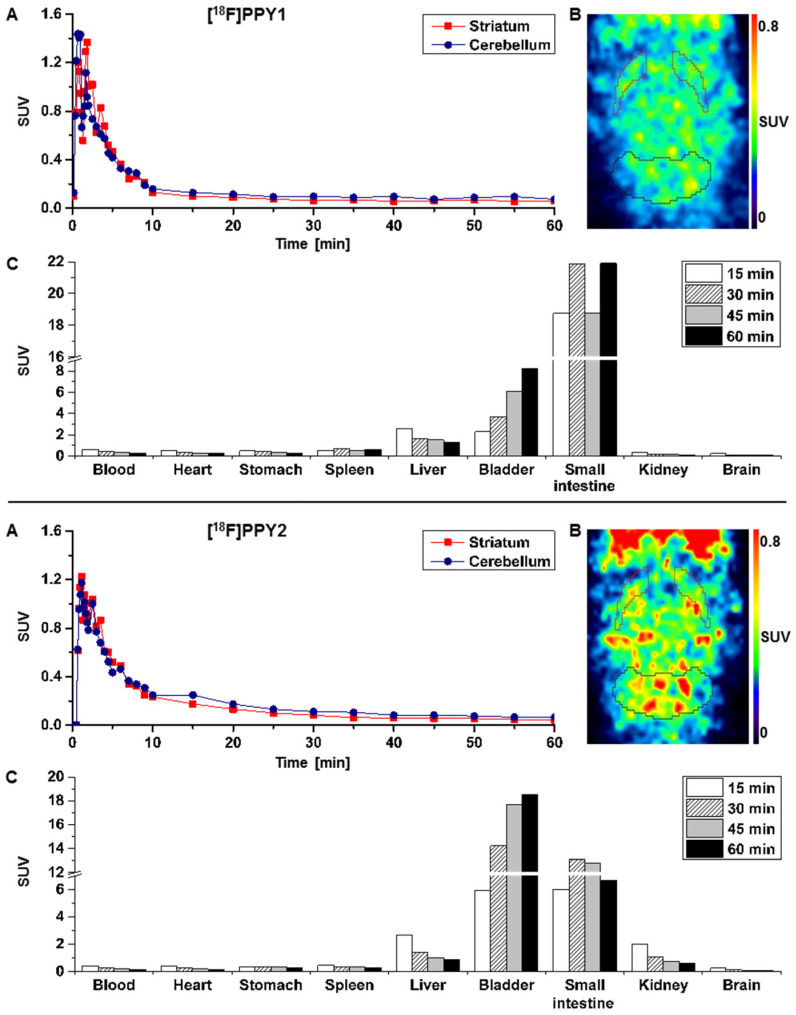
(**A**) Time–activity curves with SUVs for striatum and cerebellum; (**B**) Representative horizontal PET images (0–10 min) after injection of [^18^F]**PPY1** and [^18^F**]PPY2** in CD-1 mice (n = 2, respectively). (**C**) Biodistribution of [^18^F]**PPY1** and [^18^F**]PPY2** at different time points based on PET imaging (n = 1 and n = 2, respectively).

The initial brain uptake of [^18^F]**PPY1** and [^18^F]**PPY2** in CD1-mice was rather high with a standardized uptake value (SUV) of 1.2–1.4 between 2 and 3 min, respectively, indicating a significant BBB permeability of the radiotracers. The striatum (A_2A_R-rich region) and the cerebellum (A_2A_R-poor region) shared similar kinetics i.e., an initial uptake of ~1.3 SUV followed by a fast washout (Figure 9A) for both radiotracers, presuming a lack of specific binding. The maximum SUV ratio of striatum-to-cerebellum of 1.5 and 1.3 was achieved after only 4 min for [^18^F]**PPY1** and [^18^F]**PPY2**, respectively, indicative of their lack of selectivity. Although this result is not surprising for [^18^F]**PPY1** given its lack of specific binding already in vitro, [^18^F]**PPY2** shows the same characteristic in vivo, despite its better in vitro qualities and compliance with the Lipinski’s rule of five [61] (Figure 9B). Several factors in vivo may be responsible for this discrepancy, such as the presence of radiometabolites contributing to a lower specific-to-non-specific binding ratio in vivo compared to in vitro findings, an insufficient free fraction due to plasma protein binding, or a potential efflux by the permeability-glycoprotein (P-gp). Although the initial uptake value is in favor of a first passage of the BBB, the fast washout may reflect a P-gp transportation, which is likely to happen also in higher species [62,63]. Furthermore, the insufficient selective binding of [^18^F]**PPY1** could be related to a higher *K_d_* value in mice compared to human not in compliance with the above-mentioned rule (*B*_max_/*K*_d_ > 10). Organ distribution analysis displayed a low radiotracer uptake in the heart, spleen, stomach and, to a lesser extent, in the liver (initial SUV < 3), while an accumulation occurred in the small intestine (SUV_60 min_ 21.9 and 6.7 for [^18^F]**PPY1** and [^18^F]**PPY2**, respectively) and the bladder (SUV_60 min_ 8.2 and 18.5 for [^18^F]**PPY1** and [^18^F]**PPY2**, respectively) indicating excretion via the hepatobiliary and the urinary pathways (Figure 9C).

## 3. Materials and Methods

### 3.1. General Information

All chemicals and reagents were purchased from commercially available sources and used without further purification. Moisture-sensitive reactions were conducted under dry argon with oven-dried glassware and anhydrous solvents. Reaction progress was monitored by thin-layer chromatography (TLC) using Alugram^®^ SIL G/UV_254_ pre-coated plates (Macherey-Nagel, Düren, Germany). The spots were identified by using an UV lamp or by dipping the plates into a potassium permanganate solution (3 g KMnO_4_, 20 g K_2_CO_3_, 0.25 mL glacial acid, 300 mL water). For purification of products flash column chromatography was used with silica gel 40–63 μm from VWR International Chemicals (Darmstadt, Germany). The purity of all the tested compounds was ≥95% as determined by an LC-MS system including a DAD detector [Dionex Ultimate 3000 system incorporating a LPG-3400SD pump, a WPS-3000 TSL autosampler, a TCC-3000SD column compartment, a DAD 3000 diode array detector and a MSQ Plus low resolution mass spectrometer (Thermo Fisher Scientific Inc., Waltham, MA, USA), column: Reprosil-Pur Basic HD (150 × 3 mm; 3 µm; Dr. Maisch GmbH, Ammerbuch, Germany), gradient: 10–90–10% MeCN/ 20 mM NH_4_OAc_aq._ (*v/v*), run time: 15 min, flow rate: 0.6 mL/min, UV-detection: 254 nm]. ^1^H-, ^13^C- and ^19^F-NMR spectra were recorded on VARIAN Mercury plus (300 MHz for ^1^H-NMR, 75 MHz for ^13^C-NMR, 282 MHz for ^19^F-NMR; Agilent Technologies, Palo Alto, CA, USA) and BRUKER DRX-400 (400 MHz for ^1^H-NMR, 100 MHz for ^13^C-NMR, 377 MHz for ^19^F-NMR; Bruker, Billerica, MA, USA); chemical shifts (δ) in parts per million (ppm) are related to internal tetramethylsilane and coupling constants (*J*) are given with 0.1 Hz (see Supporting information, Figure S2–S45). High resolution mass spectra (HRFT-MS) were recorded on a FT-ICR APEX II spectrometer (Bruker Daltonics, Bruker Corporation, Billerica, MA, USA) using electrospray ionization (ESI).

### 3.2. Chemical Synthesis

#### 3.2.1. Synthesis of 4-(furan-2-yl)-1H-pyrazolo[3,4-d]pyrimidin-6-amine (3)

The synthesis was performed as described by Gillespie et al. [39,40] with minor modifications: Phosphoryl chloride (8 eq) was cooled to 0 °C and then, absolute DMF (2.5 eq) was added dropwise over a 20 min period. Afterwards, 2-amino-6-hydroxy-pyrimidin-4(3*H*)-one 1 (1 eq) was added over 20 min at RT. The reaction mixture was stirred at 90 °C for 3 h. The solution was cooled to RT and poured into 500 mL ice water. The mixture was refrigerated overnight and the precipitate was filtered and dried to provide 2-amino-4-chloro-6-oxo-1,6-dihydropyrimidine-5-carbaldehyde **1** as colorless solid, which was used directly in the next step. A solution of the aldehyde **1** (1 eq) in THF was treated with triethylamine (1.05 eq) and anhydrous hydrazine (1 eq) was added dropwise over 25 min. The reaction mixture was stirred at RT for 1 h. After the addition of water, the precipitate was filtered and the filtrate was evaporated to remove about 80% of THF under reduced pressure. Water was additionally added to the mixture and the resulting precipitate was filtered and dried under vacuum. The solid was then dissolved in a minimum of hot DMF and precipitated by the addition of water to provide 4-chloro-1*H*-pyrazolo[3,4-*d*]pyrimidin-6-amine **2** as colorless solid which was used directly in the next step. To a solution of **2** (1 eq) in DMF were added 2-(tributylstannyl)-furan (1 eq) and bis(triphenylphos-phine)palladium(II) dichloride (PdCl_2_(PPh_3_)_2_, 0.05 eq) under argon. The resulting mixture was stirred at 90 °C overnight and, then, the solvent was removed by rotatory evaporation. The remaining residue was dissolved in ethyl acetate (EA) and filtered over celite, which was washed with a mixture of EA and water (1:1, *v/v*). The aqueous solution was extracted with EA and the combined organic phases were washed with brine, dried over anhydrous Na_2_SO_4_, filtered and evaporated to dryness. The crude product was purified by flash chromatography (silica, gradient CH_2_Cl_2_/MeOH 1:0 → 98:2 → 96:4 → 94:6 → 92:8 → 9:1) to give **3** (57% over 3 steps) as yellow solid. TLC (EA/petrolether (PE), 1:1): *R*_f_ = 0.1; ^1^H-NMR (400 MHz, CDCl_3_): *δ* = 13.02 (s, 1H, NH), 8.22 (d, *J* = 1.3 Hz, 1H, 1), 8.04 (dd, *J =* 0.8, 1.8 Hz, 1H, 4), 7.41 (dd, *J* = 0.8, 3.5 Hz, 1H, 2), 6.76 (dd, *J* = 1.8, 3.5 Hz, 1H, 3), 6.74 (s, NH_2_); ^13^C-NMR (101 MHz, CDCl_3_): *δ* = 162.50, 158.24, 152.13, 150.48, 146.94, 134.31, 114.06, 113.12, 102.29.

#### 3.2.2. General Procedure A

To a solution of the corresponding (hetero)arene bromide (1 eq, 0.25 mmol) in 1 mL DMF were added compound **3** (50 mg, 1 eq, 0.25 mmol) and potassium carbonate (3 eq, 0.75 mmol). The reaction mixture was stirred overnight at RT. After the addition of 20 mL water, the aqueous phase was extracted with EA (3 × 20 mL) and the combined organic phases were washed with brine (20 mL), dried over anhydrous MgSO_4_, filtered and evaporated to dryness. The crude product was purified by flash chromatography (silica, gradient EA/PE 1:2 → 1.5:2 → 1:1) to afford the corresponding fluorinated derivative as yellow solid.

1-(4-Fluorobenzyl)-4-(furan-2-yl)-1*H*-pyrazolo[3,4-*d*]pyrimidin-6-amine (**PPY1**): yield: 60%; TLC (EA/PE, 1:1): *R*_f_ = 0.23; LC-MS: t_R_ = 9.9 min, >99%; ^1^H-NMR (400 MHz, CDCl_3_): δ = 8.30 (s, 1H), 7.85–7.61 (m, 2H), 7.32 (dd, *J* = 5.4, 8.5 Hz, 2H), 7.00 (t, *J* = 8.7 Hz, 2H), 6.69-6.63 (m, 1H), 5.68 (s, NH_2_), 5.43 (s, 2H); ^19^F-NMR (377 MHz, CDCl_3_): δ = −114,32; ^13^C-NMR (101 MHz, CDCl_3_): δ = 162.59 (d, *J* = 246.4 Hz), 159.95, 155.79, 147.18, 147.03, 146.89, 135.54, 132.26 (d, *J* = 3.0 Hz), 129.92 (d, *J* = 8.2 Hz, 2C), 116.79, 115.76 (d, *J* = 21.6 Hz, 2C), 113.25, 103.75, 49.66; HRFT-MS (ESI+): m/z = 309.1022 (calcd. 309.1026 for [M]^+^).

1-(2-Fluorobenzyl)-4-(furan-2-yl)-1*H*-pyrazolo[3,4-*d*]pyrimidin-6-amine (**PPY2**): yield: 51%; TLC (EA/PE, 1:1): *R*_f_ = 0.33; LC-MS: t_R_ = 9.9 min, >99%; ^1^H-NMR (400 MHz, CDCl_3_): δ = 8.32 (s, 1H), 7.86-7.59 (m, 2H), 7.32–7.21 (m, 2H), 7.18–7.02 (m, 3H), 6.70–6.62 (m, 1H), 5.79–5.33 (m, 4H); ^19^F-NMR (377 MHz, CDCl_3_): δ = −118,23; ^13^C-NMR (101 MHz, CDCl_3_): δ = 160.62 (d, *J* = 247.6 Hz), 160.16, 156.17, 150.65, 147.61, 147.11, 135.60, 129.91 (d, *J* = 8.5 Hz), 129.85 (d, *J* = 13.0 Hz), 124.45 (d, *J* = 3.5 Hz), 123.65 (d, *J* = 15.6 Hz), 115.66 (d, *J* = 21.2 Hz), 117.79, 113.19, 103.70, 43.90; HRFT-MS (ESI+): m/z = 309.1026 (calcd. 309.1026 for [M]^+^).

1-(3-Fluorobenzyl)-4-(furan-2-yl)-1*H*-pyrazolo[3,4-*d*]pyrimidin-6-amine (**PPY3**): yield: 38%; TLC (EA/PE, 1:1): *R*_f_ = 0.27; LC-MS: t_R_ = 9.9 min, >99%; ^1^H-NMR (400 MHz, CDCl_3_): δ = 8.29 (s, 1H), 7.74 (d, *J* = 1.7 Hz, 1H), 7.56 (s, 1H), 7.31–7.22 (m, 1H), 7.08 (d, *J* = 1.2, 7.7 Hz, 1H), 7.02–6.90 (m, 2H), 6.64 (dd, *J* = 1.7, 3.6 Hz, 1H), 5.67–5.31 (m, 4H); ^19^F-NMR (377 MHz, CDCl_3_): δ = −112.56; ^13^C-NMR (101 MHz, CDCl_3_): δ = 163.04 (d, *J* = 246.6 Hz), 160.91, 156.16, 146.65, 143.02, 139.14 (d, *J* = 7.3 Hz), 135.29, 130.36 (d, *J* = 8.2 Hz), 123.49 (d, *J* = 2.8 Hz), 115.57, 115.03 (d, *J* = 2.7 Hz), 114.81 (d, *J* = 1.0 Hz), 112.95, 103.90, 77.48, 49.67; HRFT-MS (ESI+): m/z = 309.1024 (calcd. 309.1026 for [M]^+^).

1-((2-Fluoropyridin-3-yl)methyl)-4-(furan-2-yl)-1*H*-pyrazolo[3,4-*d*]pyrimidin-6-amine (**PPY4**): yield: 62%; TLC (EA/PE, 1:1): *R*_f_ = 0.21; LC-MS: t_R_ = 8.8 min, >99%;^1^H-NMR (400 MHz, CDCl_3_): δ = 8.32 (s, 1H), 8.14 (dt, *J* = 5.0, 1.4 Hz, 1H), 7.83–7.71 (m, 1H), 7.56 (s, 1H), 7.47 (ddd, *J =* 9.5, 7.4, 1.9 Hz, 1H), 7.11 (ddd, *J* = 7.1, 4.9, 1.7 Hz, 1H), 6.66 (dd, *J* = 3.6, 1.7 Hz, 1H), 5.60–5.20 (m, 4H); ^19^F-NMR (377 MHz, CDCl_3_): δ = −71.66; ^13^C-NMR (101 MHz, CDCl_3_): δ = 161.12 (d, *J* = 239.6 Hz), 156.49, 154.42, 147.12 (d, *J =* 14.4 Hz), 146.74, 140.19 (d, *J =* 4.7 Hz), 138.74, 135.71, 131.00, 121.79 (d, *J* = 4.4 Hz), 119.00 (d, *J* = 29.2 Hz), 115.83, 113.05, 103.84, 43.62; HRFT-MS (ESI+): m/z = 311.1053 (calcd. 311.1057 for [M+H]^+^).

1-((6-Fluoropyridin-3-yl)methyl)-4-(furan-2-yl)-1*H*-pyrazolo[3,4-*d*]pyrimidin-6-amine (**PPY5**): yield: 73%; LC-MS: t_R_ = 8.7 min, >99%; ^1^H-NMR (400 MHz, CDCl_3_): δ = 8.37–8.20 (m, 2H), 7.87–7.59 (m, 3H), 6.88 (dd, *J* = 8.5, 2.9 Hz, 1H), 6.67 (dd, *J* = 3.7, 1.7 Hz, 1H), 5.46 (s, 2H); ^19^F-NMR (377 MHz, CDCl_3_): δ = −68.63; ^13^C-NMR (101 MHz, CDCl_3_): δ = 160.92 (d, *J* = 247.2 Hz), 155.77, 153.58, 147.46 (d, *J* = 15.1 Hz), 147.12, 143.38, 141.23 (d, *J* = 8.0 Hz), 137.30, 135.69, 129.74 (d, *J* = 5.4 Hz), 113.95, 113.12, 109.67 (d, *J* = 37.6 Hz), 103.63, 46.91; HRFT-MS (ESI+): m/z = 311.1053 (calcd. 311.1057 for [M+H]^+^).

1-((6-Fluoropyridin-2-yl)methyl)-4-(furan-2-yl)-1*H*-pyrazolo[3,4-*d*]pyrimidin-6-amine (**PPY6**): yield: 54%; TLC (EA/PE, 1:1): *R*_f_ = 0.27; LC-MS: t_R_ = 8.8 min, >97%; ^1^H-NMR (400 MHz, CDCl_3_): δ = 8.35 (s, 1H), 7.76 (s, 1H), 7.69 (q, *J* = 7.9 Hz, 1H), 7.57 (s, 1H), 6.81 (td, *J* = 6.9, 5.6, 2.4 Hz, 2H), 6.66 (dd, *J =* 3.6, 1.7 Hz, 1H), 5.65–5.26 (m, 4H); ^19^F-NMR (377 MHz, CDCl_3_): δ = −66.81; ^13^C-NMR (101 MHz, CDCl_3_): δ = 163.30 (d, *J* = 241.0 Hz), 161.08, 156.63, 155.61 (d, *J* = 13.7 Hz), 150.61, 146.77, 146.71, 141.98 (d, *J* = 7.7 Hz), 135.68, 118.70 (d, *J* = 4.0 Hz), 115.66, 113.03, 108.55 (d, *J* = 36.7 Hz), 103.87, 50.97; HRFT-MS (ESI+): m/z = 311.1054 (calcd. 311.1057 for [M+H]^+^).

1-((2-Fluoropyridin-4-yl)methyl)-4-(furan-2-yl)-1*H*-pyrazolo[3,4-*d*]pyrimidin-6-amine (**PPY7**): yield: 53%; TLC (EA/PE, 1:1): *R*_f_ = 0.18; LC-MS: t_R_ = 8.8 min, >99; ^1^H-NMR (400 MHz, CDCl_3_): δ = 8.36 (s, 1H), 8.16 (d, *J* = 5.2 Hz, 1H), 7.90–7.59 (m, 2H), 7.06 (d, *J* = 5.1 Hz, 1H), 6.76 (s, 1H), 6.69 (s, 1H), 5.92–5.28 (m, 4H); ^19^F-NMR (377 MHz, CDCl_3_): δ = −67.20; ^13^C-NMR (101 MHz, CDCl_3_): δ = 164.24 (d, *J* = 239.5 Hz), 160.27, 160.09, 156.32, 151.17 (d, *J* = 10.6 Hz), 148.24 (d, *J* = 15.2 Hz), 147.39, 140.95, 136.21, 120.22 (d, *J* = 4.1 Hz), 117.00, 113.37, 108.41 (d, *J* = 38.3 Hz), 103.63, 48.73; HRFT-MS (ESI+): m/z = 311.1054 (calcd. 311.1057 for [M+H]^+^).

1-(4-Bromo-2-fluorobenzyl)-4-(furan-2-yl)-1*H*-pyrazolo[3,4-*d*]pyrimidin-6-amine (**PPY8**): yield: 44%; LC-MS: t_R_ = 10.7 min, >99%; ^1^H-NMR (400 MHz, CDCl_3_): δ = 8.28 (s, 1H), 7.73 (d, *J* = 1.8 Hz, 1H), 7.44 (d, *J* = 3.5 Hz, 1H), 7.31-7.22 (m, 1H), 7.19 (dd, *J* = 8.3, 1.9 Hz, 1H), 6.98 (t, *J =* 8.1 Hz, 1H), 6.63 (dd, *J =* 3.6, 1.8 Hz, 1H), 5.48 (s, 2H), 5.38 (s, NH_2_); ^19^F-NMR (377 MHz, CDCl_3_): δ = −115.35; ^13^C-NMR (101 MHz, CDCl_3_): δ = 161.56, 160.30 (d, *J* = 252.1 Hz), 156.43, 151.91, 151.16, 146.32, 135.25, 131.02 (d, *J* = 4.4 Hz), 127.77 (d, *J* = 3.7 Hz), 123.21 (d, *J* = 14.8 Hz), 122.09 (d, *J* = 9.4 Hz), 119.30 (d, *J* = 24.5 Hz), 114.82, 112.78, 103.96, 43.34; HRFT-MS (ESI+): m/z = 388.0202 (calcd. 388.0209 for [M+H]^+^).

1-(2-Bromo-4-fluorobenzyl)-4-(furan-2-yl)-1*H*-pyrazolo[3,4-*d*]pyrimidin-6-amine (**PPY9**): yield: 54%; TLC (EA/PE, 1:1): *R*_f_ = 0.52; LC-MS: t_R_ = 10.7 min, >99%; ^1^H-NMR (500 MHz, CDCl_3_): δ = 8.33 (s, 1H), 7.76 (d, *J* = 1.7 Hz, 1H), 7.55 (s, 1H), 7.34 (dd, *J* = 2.6, 8.1 Hz, 1H), 6.93 (td, *J* = 2.6, 8.3 Hz, 1H), 6.84 (dd, *J* = 5.9, 8.7 Hz, 1H), 6.66 (dd, *J* = 1.7, 3.6 Hz, 1H), 5.54 (s, 2H), 5.40 (s, NH_2_); ^19^F-NMR (282 MHz, CDCl_3_): δ = −112.80; ^13^C-NMR (101 MHz, CDCl_3_): δ = 161.74 (d, *J* = 250.6 Hz), 161.35, 156.47, 151.77, 151.02, 146.18, 135.20, 131.97 (d, *J* = 3.5 Hz), 129.85 (d, *J* = 8.6 Hz), 122.63 (d, *J* = 9.6 Hz), 120.06 (d, *J* = 24.6 Hz), 114.75 (d, *J* = 21.1 Hz), 114.68, 112.65, 103.77, 49.32; HRFT-MS (ESI+): m/z = 388.0200 (calcd. 388.0209 for [M+H]^+^).

1-(3-Bromo-5-fluorobenzyl)-4-(furan-2-yl)-1*H*-pyrazolo[3,4-*d*]pyrimidin-6-amine (**PPY10**): yield: 56%; TLC (EA/PE, 1:1): *R*_f_ = 0.52; LC-MS: t_R_ = 10.8 min, >99%; ^1^H-NMR (400 MHz, CDCl_3_): δ = 8.31 (s, 1H), 7.84–7.69 (m, 1H), 7.57 (s, 1H), 7.25 (s, 1H), 7.15 (dt, *J* = 2.1, 8.1 Hz, 1H), 6.94 (dt, *J =* 1.8, 9.0 Hz, 1H), 6.66 (dd, *J* = 1.7, 3.6 Hz, 1H), 5.49-5.12 (m, 4H); ^19^F-NMR (282 MHz, CDCl_3_): δ = −110.10; ^13^C-NMR (101 MHz, CDCl_3_): δ = 162.81 (d, *J* = 251.5 Hz), 158.35, 156.24, 146.67, 142.30, 140.57, 135.58 (d, *J* = 2.4 Hz), 128.20, 126.82 (d, *J* = 3.0 Hz), 122.97 (d, *J* = 9.8 Hz), 118.74 (d, *J =* 24.5 Hz), 114.02 (d, *J* = 22.1 Hz), 113.01, 111.81, 103.92, 49.14; HRFT-MS (ESI+): m/z = 388.0199 (calcd. 388.0209 for [M+H]^+^).

1-(2-Bromo-6-fluorobenzyl)-4-(furan-2-yl)-1*H*-pyrazolo[3,4-*d*]pyrimidin-6-amine (**PPY11**): yield: 51%; LC-MS: t_R_ = 10.3 min, >95%; ^1^H-NMR (400 MHz, CDCl_3_): δ = 8.22 (s, 1H), 7.71 (dd, *J* = 1.7, 0.8 Hz, 1H), 7.48 (s, 1H), 7.41 (dt, *J* = 8.0, 1.1 Hz, 1H), 7.20 (td, *J* = 8.2, 5.9 Hz, 1H), 7.08 (t, *J* = 9.4, 8.3, 1.2 Hz, 1H), 6.62 (dd, *J* = 3.6, 1.7 Hz, 1H), 5.61 (s, 2H), 5.45 (s, 2H); ^19^F-NMR (282 MHz, CDCl_3_): δ = −111.58; ^13^C-NMR (101 MHz, CDCl_3_): δ = 162.16 (d, *J* = 252.7 Hz), 160.91, 156.29, 151.68, 150.73, 146.35, 135.12, 130.85 (d, *J* = 9.4 Hz), 128.99 (d, *J* = 3.4 Hz), 125.93 (d, *J* = 4.0 Hz), 123.59 (d, *J* = 16.4 Hz), 115.10 (d, *J* = 22.6 Hz), 115.05, 112.78, 103.78, 43.71; HRFT-MS (ESI+): m/z = 388.0202 (calcd. 388.0209 for [M+H]^+^).

2-((6-Amino-4-(furan-2-yl)-1*H*-pyrazolo[3,4-*d*]pyrimidin-1-yl)methyl)-5-fluoro-benzonitrile (**PPY12**): yield: 65%; TLC (EA/PE, 1:1): *R*_f_ = 0.38; LC-MS: t_R_ = 9.8 min, >98%; ^1^H-NMR (400 MHz, CDCl_3_): δ = 8.30 (s, 1H), 7.73 (d, *J* = 1.7 Hz, 1H), 7.39 (d, *J* = 3.2 Hz, 1H), 7.37 (d, *J* = 2.8 Hz, 1H), 7.23–7.09 (m, 2H,), 6.63 (dd, *J* = 1.8, 3.5 Hz, 1H), 5.67 (s, 2H), 5.29 (s, NH_2_); ^19^F-NMR (377 MHz, CDCl_3_): δ = −111.834; ^13^C-NMR (101 MHz, CDCl_3_): δ = 161.40 (d, *J* = 250.4 Hz), 161.78, 156.61, 152.10, 151.54, 146.07, 136.52 (d, *J* = 3.6 Hz), 135.38, 130.67 (d, *J* = 8.5 Hz), 120.76 (d, *J* = 21.3 Hz), 119.66 (d, *J* = 24.8 Hz), 116.06, 116.04, 114.34, 113.02, 112.97 (d, *J* = 9.4 Hz), 103.87, 47.25; HRFT-MS (ESI+): m/z = 335.1051 (calcd. 335.1057 for [M+H]^+^).

4-((6-Amino-4-(furan-2-yl)-1*H*-pyrazolo[3,4-*d*]pyrimidin-1-yl)methyl)-3-fluoro-benzonitrile (**PPY13**): yield: 77%; TLC (EA/PE, 1:1): *R*_f_ = 0.38; LC-MS: t_R_ = 9.7 min, >98%; ^1^H-NMR (400 MHz, CDCl_3_): δ = 8.32 (s, 1H), 7.76 (d, *J* = 1.7 Hz, 1H), 7.69-7.49 (m, 1H), 7.39 (dd, *J* = 7.9, 2.6 Hz, 1H), 7.25-7.11 (m, 2H), 6.66 (dd, *J* 3.6, 1.7 Hz, 1H), 5.67 (s, 2H), 5.38 (s, NH_2_); ^19^F-NMR (377 MHz, CDCl_3_): δ = −111.64; ^13^C-NMR (101 MHz, CDCl_3_): δ = 161.59 (d, *J* = 250.6 Hz), 161.11, 156.54, 146.73, 146.71, 136.40 (d, *J* = 3.1 Hz), 135.85, 131.82, 130.89 (d, *J* = 8.3 Hz), 120.92 (d, *J* = 21.4 Hz), 119.85 (d, *J* = 24.9 Hz), 116.15 (d, *J* = 2.8 Hz), 115.65, 113.79, 113.21 (d, *J =* 9.2 Hz), 113.01, 103.84, 77.48, 77.16, 76.84, 47.48; HRFT-MS (ESI+): m/z = 335.1047 (calcd. 335.1057 for [M+H]^+^).

3-((6-Amino-4-(furan-2-yl)-1*H*-pyrazolo[3,4-*d*]pyrimidin-1-yl)methyl)-5-fluoro-benzonitrile (**PPY14**): yield: 85%; TLC (EA/PE, 1:1): *R*_f_ = 0.38; LC-MS: t_R_ = 9.7 min, >99%; ^1^H-NMR (500 MHz, CDCl_3_): δ = 8.31 (s, 1H), 7.75 (s, 1H), 7.49 (s, 1H), 7.39 (t, *J* = 1.4 Hz, 1H), 7.28 (d, *J* = 1.5 Hz, 1H), 7.26-7.23 (m, 1H), 6.65 (dd, *J* = 1.8, 3.6 Hz, 1H), 5.48 (s, 1H), 5.32 (s, NH_2_); ^19^F-NMR (282 MHz, CDCl_3_): δ = −109.05 (t, *J* = 8.4 Hz); ^13^C-NMR (101 MHz, CDCl_3_): δ = 162.46 (d, *J* = 251.3 Hz), 161.51, 156.42, 146.55, 141.19 (d, *J* = 6.5 Hz), 139.68, 135.69, 127.49 (d, *J* = 3.3 Hz), 125.68 (d, *J* = 21.9 Hz), 118.65 (d, *J =* 24.7 Hz), 117.43 (d, *J* = 3.1 Hz), 115.31, 114.32 (d, *J* = 9.8 Hz), 112.91, 103.99, 77.48, 48.91; HRFT-MS (ESI+): m/z = 335.1049 (calcd. 335.1057 for [M+H]^+^).

2-((6-Amino-4-(furan-2-yl)-1*H*-pyrazolo[3,4-*d*]pyrimidin-1-yl)methyl)-3-fluoro-benzonitrile (**PPY15**): yield: 59%; TLC (EA/PE, 1:1): *R*_f_ = 0.38; LC-MS: t_R_ = 9.4 min, >99%; ^1^H-NMR (300 MHz, CDCl_3_): δ = 8.24 (s, 1H), 7.72 (d, J = 1.7 Hz, 1H), 7.58-7.27 (m, 4H), 6.63 (dd, *J* = 3.6, 1.8 Hz, 1H), 5.68 (s, 2H), 5.41 (s, NH_2_); ^19^F-NMR (282 MHz, CDCl_3_): δ = −112.33 (dd, *J* = 9.3, 5.1 Hz); ^13^C-NMR (75 MHz, CDCl_3_): δ = 161.40 (d, *J* = 252.4 Hz), 161.24, 156.56, 151.72, 150.78, 146.36, 135.45, 130.81 (d, *J* = 9.0 Hz), 129.41 (d, *J* = 3.7 Hz), 126.82 (d, *J* = 17.5 Hz), 120.79 (d, *J* = 22.0 Hz), 116.43 (d, *J* = 4.0 Hz), 115.54 (d, *J* = 5.4 Hz), 114.98, 112.80, 103.82, 42.43; HRFT-MS (ESI+): m/z = 335.1049 (calcd. 335.1057 for [M+H]^+^).

1-(3-(2-Fluoroethyl)benzyl)-4-(furan-2-yl)-1*H*-yrazolo[3,4-*d*]pyrimidin-6-amine (**PPY16**): yield: 51%; LC-MS: t_R_ = 10.0 min, >99%; ^1^H-NMR (400 MHz, CDCl_3_): δ = 8.27 (s, 1H), 7.77-7.65 (m, 1H), 7.43 (d, *J* = 3.5 Hz, 2H), 7.34-7.11 (m, 4H), 6.63 (dd, *J* = 3.5, 1.7 Hz, 1H), 5.46 (s, 2H), 5.33 (s, NH_2_), 4.59 (dt, *J* = 47.1, 6.6 Hz, 2H), 2.97 (dt, *J* = 23.0, 6.6 Hz, 2H); ^19^F-NMR (282 MHz, CDCl_3_): δ = −114.63; ^13^C-NMR (75 MHz, CDCl_3_): δ = 146.42, 145.93, 137.69 (d, *J =* 6.3 Hz), 137.06, 134.99, 129.82 (d, *J* = 8.1 Hz), 129.46, 129.04, 128.75, 128.58, 127.60 (d, *J* = 13.3 Hz), 126.37, 115.83, 112.84, 104.01, 84.07 (d, *J* = 169.0 Hz), 50.14, 36.93 (d, *J =* 20.4 Hz) HRFT-MS (ESI+): m/z = 338.1412 (calcd. 338.1417 for [M+H]^+^).

1-(3-(2-Fluoroethoxy)benzyl)-4-(furan-2-yl)-1*H*-pyrazolo[3,4-*d*]pyrimidin-6-amine (**PPY17**): yield: 54%; LC-MS: t_R_ = 9.8 min, >99%; ^1^H-NMR (400 MHz, CDCl_3_): δ = 8.27 (s, 1H), 7.73 (dd, *J* = 1.7, 0.8 Hz, 1H), 7.48 (s, 1H), 7.23 (t, *J* = 7.9 Hz, 1H), 6.97-6.78 (m, 3H), 6.64 (dd, *J* = 3.6, 1.8 Hz, 1H), 5.64-5.14 (m, 4H), 4.84-4.57 (m, 2H), 4.27-4.05 (m, 2H); ^19^F-NMR (377 MHz, CDCl_3_): δ = −223.92; ^13^C-NMR (101 MHz, CDCl_3_): δ = 161.27, 158.78, 156.17, 151.79, 150.85, 146.36, 138.48, 134.96, 129.94, 120.82, 115.01, 114.30, 113.99, 112.81, 104.03, 81.98 (d, *J* = 170.8 Hz), 67.17 (d*, J* = 20.6 Hz), 50.08; HRFT-MS (ESI+): m/z = 354.1316 (calcd. 354.1366 for [M+H]^+^).

1-(3-(3-Fluoropropoxy)benzyl)-4-(furan-2-yl)-1*H*-pyrazolo[3,4-*d*]pyrimidin-6-amine (**PPY18**): yield: 61%; LC-MS: t_R_ = 11.0 min, >95%; ^1^H-NMR (400 MHz, CDCl_3_): δ = 8.36 (s, 1H), 7.81-7.74 (m, 1H), 7.46 (d, *J* = 8.8 Hz, 1H), 7.38 (dd, *J* = 8.6, 5.4 Hz, 2H), 6.98 (t, *J* = 8.7 Hz, 1H), 6.70 (d, *J* = 3.0 Hz, 1H), 6.37 (d, *J =* 3.0 Hz, 1H), 5.53 (s, 2H), 4.65-4.46 (m, 4H), 3.93 (t, *J* = 6.1 Hz, 2H), 2.06 (dt, *J* = 26.1, 5.9 Hz, 2H); ^19^F-NMR (377 MHz, CDCl_3_): δ = −114.61; ^13^C-NMR (101 MHz, CDCl_3_): δ = 163.75, 161.30, 159.14, 156.07, 151.66, 146.43, 135.03, 132.57 (d, *J* = 3.1 Hz), 129.83 (d, *J* = 8.2 Hz), 120.41, 115.69 (d, *J* = 21.6 Hz), 115.09, 114.03 (d, *J* = 33.8 Hz), 112.84, 104.03, 80.86 (d, *J =* 164.4 Hz), 63.57 (d, *J =* 5.1 Hz), 49.52, 30.12 (d, *J* = 57.4 Hz); LRFT-MS (ESI+): m/z = 406.29 (calcd. 406.11 for [M+K]^+^).

1-(2-Fluorophenethyl)-4-(furan-2-yl)-1*H*-pyrazolo[3,4-*d*]pyrimidin-6-amine (**PPY19**): yield: 45%; TLC (EA/PE, 1:1): *R*_f_ = 0.46; LC-MS: t_R_ = 10.1 min, >99%; ^1^H-NMR (400 MHz, CDCl_3_): δ = 8.24 (s, 1H), 7.73 (dd, *J* = 0.8, 1.7 Hz, 1H), 7.54 (s, 1H), 7.20-7.13 (m, 1H), 7.10-6.87 (m, 3H), 6.63 (dd, *J* = 1.7, 3.5 Hz, 1H), 5.28 (s, NH_2_), 4.61-4.48 (m, 2H), 3.25 (t, *J* = 7.4 Hz, 2H); ^19^F-NMR (377 MHz, CDCl_3_): δ = −118.28; ^13^C-NMR (101 MHz, CDCl_3_): δ = 161.52 (d, *J =* 245.5 Hz), 161.02, 156.02, 151.83, 150.65, 146.23, 134.40, 131.18 (d, *J* = 5.1 Hz), 128.57 (d, *J* = 8.1 Hz), 125.22 (d, *J* = 15.9 Hz), 124.12 (d, *J* = 3.5 Hz), 115.39 (d, *J* = 21.9 Hz), 114.78, 112.76, 103.97, 46.51, 29.42; HRFT-MS (ESI+): m/z = 324.1281 (calcd. 324.1261 for [M+H]^+^).

1-Benzyl-4-(furan-2-yl)-1*H*-pyrazolo[3,4-*d*]pyrimidin-6-amine (**PPY22**): yield: 79%; TLC (EA/PE, 1:1): *R*_f_ = 0.47; LC-MS: t_R_ = 9.8 min, >97%; ^1^H-NMR (400 MHz, CDCl_3_): δ = 8.23 (s, 1H), 7.68 (d, *J* = 1.7 Hz, 1H), 7.43-7.14 (m, 6H), 6.58 (dd, *J* = 1.8, 3.6 Hz, 1H), 5.44 (s, 2H), 5.36 (s, NH_2_); ^13^C-NMR (101 MHz, CDCl_3_): δ = 161.60, 156.23, 152.11, 151.17, 146.12, 136.89, 134.76, 128.78 (2C), 127.92 (2C), 127.86, 114.50, 112.67, 104.10, 50.18; HRFT-MS (ESI+): m/z = 292.1189 (calcd. 292.1198 for [M+H]^+^).

#### 3.2.3. General Procedure B

The corresponding fluoro benzoic acid (1.1 eq, 0.28 mmol), (benzotriazol-1-yloxy)tris- (dimethylamino)phosphonium hexafluorophosphate (BOP, 1.3 eq, 0.33 mmol) and triethylamine (3 eq, 0.75 mmol) were dissolved in CH_2_Cl_2_ (3 mL) and stirred at RT for 30 min. After the addition of compound **3** (50 mg, 1 eq, 0.25 mmol), the reaction mixture was stirred overnight at RT. The solvent was removed by rotatory evaporation and the remaining residue was dissolved in EA (10 mL). After addition of 50 mL saturated NaHCO_3_ solution, the aqueous phase was extracted with EA (3x20 mL) and the combined organic phases were washed with brine (20 mL), dried over anhydrous MgSO_4_, filtered and evaporated to dryness. The crude product was purified by flash chromatography (silica, gradient EA/PE 1:2 → 1:1) to afford the corresponding fluorinated derivative as yellow solid.

(6-Amino-4-(furan-2-yl)-1*H*-pyrazolo[3,4-*d*]pyrimidin-1-yl)(2-fluorophenyl)-methanone (**PPY20**): yield: 82%; TLC (EA/PE, 1:1): *R*_f_ = 0.54; LC-MS: t_R_ = 9.4 min, >99%; ^1^H-NMR (400 MHz, DMF-d_7_): δ = 8.55 (s, 1H), 8.12 (d, *J =* 1.7 Hz, 1H), 7.83 (td, *J* = 1.8, 7.2 Hz, 1H), 7.73 (tdd, *J* = 1.8, 5.4, 7.5 Hz, 1H), 7.60 (s, NH_2_), 7.52 (dd, *J* = 0.8, 3.5 Hz, 1H), 7.47-7.38 (m, 2H), 6.85 (dd, *J* = 1.7, 3.6 Hz, 1H); ^19^F-NMR (377 MHz, DMF-d_7_): δ = −71.93 (d, *J* = 709.3 Hz); ^13^C-NMR (75 MHz, DMF d_7_): δ = 165.28, 163.91 (d, *J* = 133.6 Hz), 163.35, 160.78, 152.66, 152.33, 148.33, 140.21, 135.41 (d, *J* = 6.3 Hz), 134.63 (d, *J* = 8.7 Hz), 131.62 (d, *J* = 2.4 Hz), 125.49 (d, *J* = 3.5 Hz), 116.86 (d, *J* = 21.3 Hz), 113.88, 105.02; HRFT-MS (ESI+): m/z = 346.0715 (calcd. 346.0716 for [M+Na]^+^).

(6-Amino-4-(furan-2-yl)-1H-pyrazolo[3,4-*d*]pyrimidin-1-yl)(2-fluorophenyl)-methanone (**PPY21**): yield: 59%; TLC (EA/PE, 1:1): *R*_f_ = 0.54; LC-MS: t_R_ = 9.8 min, >99%; ^1^H-NMR (300 MHz, DMF-d_7_): δ = 8.58 (s, 1H), 8.20-8.11 (m, 3H), 7.62-7.37 (m, 5H), 6.86 (dd, *J* = 1.7, 3.5 Hz, 1H); ^19^F-NMR (282 MHz, DMF-d_7_): δ = −71.79 (d, *J* = 709.4 Hz); ^13^C-NMR (101 MHz, DMF d_7_): δ = 166.68, 165.00 (d, *J* = 164.0 Hz), 164.44, 160.53, 152.08, 151.57, 147.52, 138.68, 134.21 (d, *J =* 9.4 Hz, 2C), 130.32 (d, *J* = 2.3 Hz), 115.42 (d, *J =* 22.3 Hz, 2C), 114.68, 113.13, 104.14; HRFT-MS (ESI+): m/z = 324,0892 (calcd. 324.0897 for [M+Na]^+^).

#### 3.2.4. General Procedure C

To a solution of the corresponding bromomethyl-phenylboronic acid pinacol ester (1 eq, 0.5 mmol) in 1 mL DMF were added compound **3** (100 mg, 1 eq, 0.5 mmol) and potassium carbonate (3 eq, 1.5 mmol). The reaction mixture was then stirred overnight at RT. After evaporation to dryness, the crude product was purified by flash chromatography (silica, gradient EA/PE 1:2 → 1.5:2 → 1:1) to afford the corresponding precursor as tan solid.

4-(Furan-2-yl)-1-(4-(4,4,5,5-tetramethyl-1,3,2-dioxaborolan-2-yl)benzyl)-1*H*-pyrazolo[3,4-*d*]pyrimidin-6-amine (**6**): yield: 38%; TLC (EA/PE, 1:1): *R*_f_ = 0.62; ^1^H-NMR (400 MHz, CDCl_3_): δ = 8.25 (s, 1H), 7.79-7.65 (m, 3H), 7.38 (d, *J* = 3.5 Hz, 1H), 7.29 (d, *J* = 7.7 Hz, 2H), 6.62 (dd, *J* = 1.8, 3.6 Hz, 1H), 5.49 (s, 2H), 5.19 (s, NH_2_), 1.31 (s, 12H); ^13^C-NMR (101 MHz, CDCl_3_): δ = 161.73, 156.30, 152.33, 151.43, 149.32, 146.01, 139.93, 135.27 (2C), 134.69, 127.24 (2C), 114.22, 112.63, 104.18, 83.90, 77.48, 77.16, 76.84, 50.26 (2C), 24.97 (4C).

4-(Furan-2-yl)-1-(2-(4,4,5,5-tetramethyl-1,3,2-dioxaborolan-2-yl)benzyl)-1*H*-pyrazolo[3,4-*d*]pyrimidin-6-amine (**7**): yield: 60%; TLC (EA/PE, 1:1): *R*_f_ = 0.62; ^1^H-NMR (400 MHz, CDCl_3_): δ = 8.31 (s, 1H), 7.87 (dd, *J* = 1.6, 7.3 Hz, 1H), 7.73 (dd, *J* = 0.8, 1.8 Hz, 1H), 7.45 (d, *J* = 3.4 Hz, 1H), 7.33-7.18 (m, 3H), 6.69-6.61 (m, 2H), 5.86 (s, 2H), 5.44 (s, NH_2_), 1.36 (s, 12H); ^13^C-NMR (101 MHz, CDCl_3_): δ = 161.44, 156.50, 152.08, 150.97, 146.14, 143.21, 136.34, 136.22, 134.63, 131.45, 126.67, 126.33, 114.57, 112.71, 104.02, 83.97 (2C), 49.90, 25.06 (4C).

### 3.3. Docking Stimulation

Molecular docking studies were carried out using GOLD (Genetic Optimization for Ligand Docking) 5.5 program from Cambridge Crystallographic Data Center (CCDC, Cambridge, UK). GOLD uses a genetic algorithm for docking ligands into protein binding sites to explore the full range of ligand conformational flexibility with partial flexibility of the active site of the protein. The X-ray crystallographic chimeric protein structure of A_2A_R-BRIL in complex with the antagonist **ZM241385** (PDB ID: 4EIY) was considered for the purpose of docking stimulation. Among the several other crystal structures in the Protein Data Bank (PDB), this structure was particularly selected due to the highest resolution of all available non-thermostabilized structures. The A_2A_R protein was prepared by using the protein preparation wizard tool implemented in the GOLD software that removes all water molecules and adds hydrogen atoms to the protein structure. After the definition of the active site with a 4 Å radius around the ligand **ZM241385** present in the orthosteric binding site of the A_2A_R, **ZM241385** was removed from the protein structure. The ligand preparation was carried out in CambridgeSoft Chem3D 17.0 program (PerkinElmer, Waltham, MA, USA). The energy of each compound was minimized by using the MM2 force field method. A total of ten docking runs were performed per structure and the early termination step was activated if the first three poses have a root-mean-square deviation (RMSD) value of less than 1.5 Å. Other parameters were set as default. After docking, the individual binding poses of each compound were observed and their molecular interactions within the active site were evaluated. The program Discovery Studio 2017 from BIOVIA^®^ (San Diego, CA, USA) was used to visualize the key aspects of the docking results from GOLD.3.4. Radiosynthesis.

### 3.4. Radiosynthesis

#### 3.4.1. Preparation [^18^F]TBAF Complex

No carrier added (n.c.a.) [^18^F]fluoride was produced via the [^18^O(p,n)^18^F] nuclear reaction by irradiation of an [^18^O]H_2_O target (Hyox 18 enriched water, Rotem Industries Ltd., Mishor Yamin, Israel) on a Cyclone 18/9 (iba RadioPharma Solutions, Louvain-la-Neuve, Belgium) with fixed energy proton beam using Nirta [^18^F]fluoride XL target. N.c.a. [^18^F]fluoride in 1.0 mL of water was trapped on a Sep-Pak Accell Plus QMA Carbonate Plus light cartridge (Waters GmbH, Eschborn, Germany). Then, the activity was eluted with a mixture of 300 µL water and 10 μL of an aqueous tetrabutylammonium hydrogen carbonate solution (TBAHCO_3_, 0.075 M) in a conical 4 mL vial with 1 mL MeCN. The aqueous [^18^F]fluoride was azeotropically dried under vacuum and nitrogen flow within 7–10 min using a CEM Discover PETwave Microwave (75 W, at 50–60 °C, power cycling mode, CEM GmbH, Kamp-Lintfort, Germany). Two aliquots of anhydrous MeCN (2 × 1 mL) were added during the drying procedure.

#### 3.4.2. Radiosynthesis of [^18^F]PPY1

The [^18^F]TBAF complex was dissolved in 200 µL *n*-BuOH. Cu(OTf)_2_(py)_4_ (4.9 mg in 100 µL DMA) was added and the mixture was stirred at RT for 5 min. Afterwards, the boronic acid pinacol ester precursor **6** (3 mg) in 300 µL DMA was added and the reaction mixture was heated at 110 °C for 10 min. The reaction was cooled to RT, quenched by the addition of 1 mL water and injected onto a semi-preparative RP-HPLC system (ReproSil-GOLD 120 C18 column (250 × 10 mm, particles size 10 µm), 34% MeCN/H_2_O, flow rate: 4 mL/min). The fractions containing [^18^F]**PPY1** were collected and diluted with water to a total volume of 40 mL. Thereafter, the solution was passed through a preconditioned Sep Pak^®^ C18 Plus cartridge (5 mL EtOH, 60 mL H_2_O, Waters GmbH, Eschborn, Germany), washed with 2 mL water and 2 mL 10% EtOH/H_2_O (*v/v*), and [^18^F]**PPY1** was subsequently eluted with 1.5 mL absolute ethanol. After addition of 30 µL DMSO, the solvent was reduced under a stream of nitrogen at 70 °C (up to approx. 50 μL) and [^18^F]**PPY1** was formulated in sterile isotonic saline up to a final concentration of <10% of both EtOH and DMSO (*v/v*), respectively. Radiochemical and chemical purities were assessed by radio-TLC and analytical HPLC. Molar activities were determined based on aliquots taken from the formulated radiotracer solution, the mass determination for the corresponding reference standard was performed via a calibration curve obtained under the same analytical HPLC conditions (see quality control section).

#### 3.4.3. Radiosynthesis of [^18^F]PPY2

The [^18^F]TBAF complex was dissolved in 140 µL *n*-BuOH and 60 µL DMA. Cu(OTf)_2_(py)_4_ (8.1 mg in 100 µL DMA) was added and the mixture was stirred at RT for 5 min. Afterwards, the boronic acid pinacol ester precursor **7** (5 mg) in 400 µL DMA was added and the reaction mixture was heated at 110 °C for 15 min. The reaction was cooled to RT, quenched by the addition of 1 mL water and injected onto a semi-preparative RP-HPLC [ReproSil-Pur 120 C18-AQ column (250 × 10 mm, particle size: 5 µm; Dr. Maisch HPLC GmbH, Ammerbuch-Entringen, Germany), 30% MeCN/H_2_O, flow rate: 5 mL/min]. The fractions containing [^18^F]**PPY2** were collected and diluted with water to a total volume of 40 mL. Thereafter, the solution was passed through a preconditioned Sep Pak^®^ C18 Plus cartridge (5 mL EtOH, 60 mL H_2_O, Waters GmbH, Eschborn, Germany), washed with 2 mL water and 2 mL 10% EtOH/H_2_O (*v/v*), and [^18^F]**PPY2** was subsequently eluted with 1.5 mL absolute ethanol. After the addition of 30 µL DMSO, the solvent was reduced under a stream of nitrogen at 70 °C (approx. 50 μL), and [^18^F]**PPY2** was formulated in sterile isotonic saline up to a final concentration of <10% of both EtOH and DMSO (*v/v*), respectively. Radiochemical and chemical purities were assessed by radio-TLC and analytical HPLC. Molar activities were determined based on aliquots taken from the formulated radiotracer solution, and the mass determination for the corresponding reference standard was performed via a calibration curve obtained under the same analytical HPLC conditions (see quality control section).

### 3.5. Quality Control

Radio-TLC was performed on silica gel (Polygram^®^ SIL G/UV_254_, Roth, Germany) pre-coated plates with a mixture of EA/PE 1/4 (*v/v*) as eluent. The plates were exposed to storage phosphor screens (BAS-IP MS 2025; FUJIFILM Co., Saitama, Japan) and recorded using the Amersham Typhoon RGB Biomolecular Imager (GE Healthcare Life Sciences). Images were quantified with the ImageQuant TL8.1 software (GE Healthcare Life Sciences, Solingen, Germany). HPLC analysis were performed on a JASCO LC-2000 system (Jasco Deutschland GmbH, Pfungstadt, Germany), incorporating a PU-2080Plus pump (Jasco Deutschland GmbH, Pfungstadt, Germany), AS-2055Plus auto injector (100 μL sample loop, Jasco Deutschland GmbH, Pfungstadt, Germany), and a UV-2070Plus detector coupled with a gamma radioactivity HPLC detector (Gabi Star, raytest Isotopenmessgeräte GmbH, Staubenhardt, Germany) and RP-HPLC columns (Dr. Maisch HPLC GmbH, Ammerbruch, Germany). Data analysis was performed with the Galaxie chromatography software (Agilent Technologies, Palo Alto, CA, USA). [^18^F]**PPY1**: ReproSil-GOLD 120 C18 column (250 × 4.6 mm, particle size 5 µm), eluent: MeCN/H2O, gradient mode (0–5′ 10% MeCN, 5–30′ up to 90% MeCN, 30–35′ 90% MeCN, 35–40′ up to 10% MeCN, 40–45 min 10% MeCN), flow rate: 1 mL/min, UV-detection: 254 nm. [^18^F]**PPY2**: Reprosil-Pur 120 C18-AQ column (250 × 4.6 mm; particle size: 5 µm), further conditions as for [^18^F]**PPY1**. The molar activities were determined on the basis of a calibration curve carried out under isocratic HPLC conditions (0.04–20 µg **PPY1** or **PPY2**, 34% MeCN/H_2_O, analytical ReproSil-GOLD 120 C18 column) using chromatograms obtained at 240 nm as the maximum of UV absorbance.

### 3.6. In Vitro Stability and Lipophilicity (LogD_7.4_)

The chemical stability of [^18^F]**PPY1** and [^18^F]**PPY2** was investigated in isotonic saline, phosphate-buffered saline (PBS, pH 7.4) and *n*-octanol by incubation at 37 °C. Samples were taken at 15, 30 and 60 min of incubation time and analyzed by radio-TLC and radio-HPLC. Log*D*_7.4_ values of [^18^F]**PPY1** and of [^18^F]**PPY2** were experimentally determined in *n*-octanol/PBS at RT by the shake-flask method [64]. The measurements were performed in triplicate.

### 3.7. Biological Evaluation

All experimental work including the use of animals has been conducted in accordance with the national legislation on the use of animals for research (Tierschutzgesetz (TierSchG), Tierschutz-Versuchstierverordnung (TierSchVersV)) and were approved by the Animal Care and Use Committee of Saxony (TVV 18/18 Landesdirektion Sachsen; DE24.1-5131/446/19; 20 June 2018). All animal experiments were performed with female CD-1 mice (10–12 weeks, 26–38 g) obtained from the Medizinisch-Experimentelles Zentrum (MEZ) at Universität Leipzig (Leipzig; Germany).

### 3.8. In Vitro Binding Assays

CHO-K1 cells stably transfected with the human A_1_R or A_2A_R, a donation from Prof. Karl-Norbert Klotz (Institute of Pharmacology and Toxicology; Universität Würzburg; Würzburg; Germany), were cultured in DMEM/F12 medium supplemented with 15 mM HEPES, 10% FCS, 1% L-Glutamine, 1% Penicillin/Streptomycin, and G418 as selective antibody at 0.2 mg/mL at 5% CO_2_ and 37 °C. Cells were harvested by scraping followed by centrifugation (800 rpm, 5 min), and the resulting pellet incubated in 50 mM TRIS-HCl, pH 7.4, on ice for 20 min. The crude membrane homogenate was obtained by centrifugation (15,000 rpm; 30 min, 4 °C), suspended in 50 mM TRIS-HCl, pH 7.4, and stored at −25 °C. Membrane suspension was incubated with the A_2A_R-specific [^3^H]**ZM241385** (American Radiolabelled Chemicals Inc., St. Louis, MO, USA; ART0884; A_m_ = 1.851 TBq/mmol) (*K*_i_(*h*A_2A_R) = 0.8 nM); or the A_1_-specific [^3^H]**DPCPX** (PerkinElmer, Waltham, MA, USA; NET974250UC, A_m_ = 6.068 TBq/mmol) (*K*_i_(*h*A_1_R) = 0.45 nM); the test compound at different concentrations in buffer at RT. Non-specific binding was determined by co-incubation with 10 μM **ZM241385** or 1 μM **DPCPX**. The IC_50_ values were determined by non-linear regression analysis with GraphPad Prism 4.1 (GraphPad Inc., La Jolla, CA, USA), and K_i_ values were estimated according to the Cheng-Prusoff equation with *K*_D,ZM241385_(*h*A_2A_R) = 0.8 nM and *K*_D,DPCPX_(*h*A_1_R) = 0.45 nM.

### 3.9. In Vitro Autoradiography

Brains of CD-1 mice frozen in isopentane were cut using a cryostat, thaw-mounted onto microscope slides, and after air-drying stored at −80 °C until use. Briefly, the brain sections were dried in a stream of cold air, and pre-incubated in 50 mM TRIS-HCl buffer (pH 7.4, 100 mM NaCl, 5 mM MgCl_2_, 1 mM EDTA) containing 1 µU/mL adenosine deaminase (ADA) for 15 min at RT. Afterwards, brain sections were incubated with 0.24 MBq/mL of [^18^F]**PPY1** (0.2 nM) or 0.26 MBq/mL of [^18^F]**PPY2** (5.1 nM) in buffer for 90 min at RT. Non-specific binding was determined in the presence of 10 µM **ZM241385**. Displacement of both radiotracers was evaluated with 10 µM **PPY1** and **PPY2**, respectively. Subsequently, the sections were washed twice for 5 min in ice-cold TRIS-HCl buffer, and dipped for 5 s in ice-cold deionized water. The sections were rapidly dried in a stream of cold air before being exposed overnight on an imaging plate. Developed autoradiographs were analyzed in a phosphor imager (HD-CR 35, Duerr NDT GmbH, Bietigheim-Bissingen, Germany). Quantification was performed by using 2D-densitometric analysis (AIDA 2.31 software, raytest Isotopenmessgeräte GmbH, Straubenhardt, Germany). Further data analysis was performed with GraphPad Prism 4.1 (GraphPad Inc., La Jolla, CA, USA).

### 3.10. In Vivo Metabolism

The radiotracer was administered i.v. as bolus in awake CD-1 mice (40 MBq [^18^F]**PPY1**; 30 MBq [^18^F]**PPY2**; n = 2, respectively). At 30 min post injection (p.i.), blood samples were taken retroorbitally from the anesthetized animals. Plasma was separated by centrifugation at 8000 rpm at RT for 1 min (Centrifuge 5418, Eppendorf Vertrieb Deutschland GmbH, Wesseling-Berzdorf, Germany), and brain homogenized in 1 mL water on ice (10 strokes of a PTFE plunge at 1000 rpm in a borosilicate glass cylinder; Potter S Homogenizer; B. Braun Melsungen AG, Melsungen, Germany). For protein precipitation and extraction an ice-cold mixture of acetone/water (4/1; *v/v*) was used in a ratio of 4:1 (*v/v*) of solvent to plasma or brain homogenate, respectively. The samples were vortexed for 3 min, equilibrated on ice for 5 min, and centrifuged for 5 min at 10,000 rpm. After separating the supernatant, the precipitates were washed with 100 µL of the solvent mixture and subjected to the same procedure. The combined supernatants were concentrated at 75 °C under nitrogen flow to a final volume of approx. 100 µL and analyzed by analytical radio-HPLC (see section quality control). To determine the percentage of activity in the supernatants compared to total activity, aliquots of each step as well as the precipitates were quantified by a gamma counter (Wallac Wizard 1480; PerkinElmer, Turku, Finland).

### 3.11. PET Imaging

For the time of the experiments, CD-1 mice were kept in a dedicated climatic chamber with free access to water and food under a 12:12 h dark:light cycle at a constant temperature (24 °C). The animals were anaesthetized (anesthesia unit U-410; agntho’s; Lidingö; Sweden) with isoflurane (1.8%, 0.35 L/min) delivered in a 60% oxygen/40% air mixture (Gas Blender 100 Series, MCQ instruments, Rome, Italy) and maintained at 37 °C with a thermal bed system. The formulated radiotracer was injected into the tail vein ([^18^F]**PPY1**: 3.2–4.3 MBq in 150 µL isotonic saline and <1% EtOH/DMSO; A_m_: 150 GBq/µmol; [^18^F]**PPY2**: 4.1–4.9 MBq in 150 µL isotonic saline and <1% EtOH/DMSO; A_m_: 80 GBq/µmol) followed by a 60 min PET/MR scan (PET/MR 1Tesla; nanoScan^®^, MEDISO Medical Imaging Systems, Budapest, Hungary). Each PET image was corrected for random coincidences, dead time, scatter and attenuation (AC), based on a whole body (WB) MR scan. The list mode data was sorted into sinograms using a framing scheme of 12 × 10 s, 6 × 30 s, 5 × 300 s, 9 × 600 s. The reconstruction parameters for the list mode data were 3D-ordered subset expectation maximization (OSEM), 4 iterations, 6 subsets, energy window: 400–600 keV, coincidence mode: 1–5, ring difference: 81. The mice were positioned prone in a special mouse bed (heated up to 37 °C), with the head fixed to a mouth piece for the anesthetic gas supply with isoflurane in 40% air and 60% oxygen (anesthesia unit: U-410, agnthos, Lidingö, Sweden; Gas blender: MCQ, Rome, Italy). The PET data were collected by a continuous WB scan during the entire investigation. Following the 60 min PET scan a T1 weighted WB gradient echo sequence (TR/TE: 20/6.4 ms, NEX: 1, FA: 25, FOV: 64 × 64 mm, matrix: 128 × 128, slice thickness: 0.5 mm) was performed for AC and anatomical orientation. Image registration and evaluation of the volume of interest (VOI) was done with PMOD 3.9 (PMOD technologies LLC, Zurich, Switzerland). The respective brain regions were identified using the mouse brain atlas template Ma-Benveniste-Mirrione-FDG. Spherical VOI with diameters of 1 to 2 mm were placed at the center of the liver, stomach, and spleen. Small intestine, kidney, and bladder VOI were delineated from the PET signal. Heart wall and blood were delineated from the T1 weighted image avoiding spill over from neighboring organs. The activity data is expressed as mean SUV of the overall VOI.

## 4. Conclusions

A series of novel fluorinated pyrazolo[2,3-*d*]pyrimidine derivatives (**PPY1**-**PPY21**) was synthesized to develop an ^18^F-labeled PET radiotracer for A_2A_R imaging in the brain by modifying the structure of the recently published lead compound **PPY**. Among those, the highly affine 4-fluorobenzyl ligand **PPY1** and the 2-fluorobenzyl ligand **PPY2** were chosen for ^18^F-labeling. Herein, a new approach was used for the radiosynthesis of [^18^F]**PPY1** and [^18^F]**PPY2** via an alcohol-enhanced copper-mediated one-step radiolabeling strategy starting from the corresponding boronic acid pinacol ester precursors. The in vivo metabolism study revealed 76% and 83% of intact [^18^F]**PPY1** and [^18^F]**PPY2**, respectively, in the mouse brain at 30 min after injection. In vitro autoradiography studies using [^18^F]**PPY1** and [^18^F]**PPY2** showed a specific binding in mouse striatum of 42% and 62%, respectively. In vivo PET experiments with CD-1 mice revealed rather high brain uptake of [^18^F]**PPY1** and [^18^F]**PPY2** and a lack of specific binding in striatum, suggesting their unfitness to image the A_2A_R in the brain. Based on these findings, the lead compound **PPY** will not consider further derivatization. Our ongoing work is focusing on other promising chemical scaffolds with enhanced in vivo stability and target selectivity to improve the A_2A_R imaging [65,66].

## Figures and Tables

**Table 1:** Overview of synthesized **PPY** derivatives and in-house determined in vitro binding affinities towards the A_2A_R and A_1_R subtypes; mean value ± standard derivation (n ≥ 3) or mean value (n = 2, single values are shown in brackets).

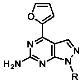	R	*K*_i_(*h*A_2A_R) in nM	*K*_i_(*h*A_1_R) in nM	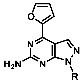	R	*K*_i_(*h*A_2A_R) in nM	*K*_i_(*h*A_1_R) in nM
**PPY1**	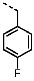	5.29 ± 1.35	220 (235, 205)	**PPY12**	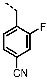	66.7 (83.4, 50.0)	810 (821, 799)
**PPY2**	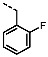	2.13 ± 0.42 (Lit.: 4) [39]	147 (165, 129) (Lit.: 264) [39]	**PPY13**	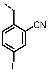	310 (369, 250)	1452 (2298, 606)
**PPY3**	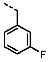	3.60 ± 1.29	132 (125, 140)	**PPY14**	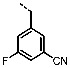	291 (297, 285)	954 (682, 1226)
**PPY4**	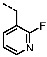	47.8 ± 4.8	822 (839, 805)	**PPY15**	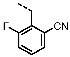	12.3 (15.6, 8.98)	470 (637, 302)
**PPY5**	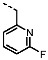	25.7 ± 0.9	689 (709, 669)	**PPY16**	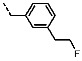	1.63 ± 0.22	69.2 ± 32.8
**PPY6**	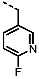	27.1 ± 2.6	566 (574, 558)	**PPY17**	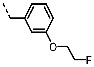	4.50 ± 0.99	215 ± 38
**PPY7**	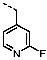	161 ± 19	2879 (3036, 2722)	**PPY18**	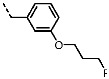	76.8 ± 12.9	527 ± 574
**PPY8**	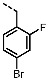	0.93 ± 0.10	31.3 ± 15.5	**PPY19**	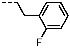	107 (114, 99.9)	245 (259, 231)
**PPY9**	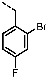	34.1 (40.5, 27.6)	766 (743, 489)	**PPY20**	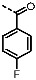	275 (287, 263)	1417 (1529, 1304)
**PPY10**	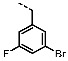	14.2 (15.1, 13.2)	160 (217, 103)	**PPY21**	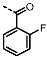	283 (293, 273)	1551 (1816, 1285)
**PPY11**	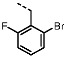	2.35 ± 0.30	162 ± 22	**PPY22**	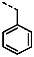	1.68 ± 0.14 (Lit.: 3) [39]	155 ± 15 (Lit.: 68) [39]

## Data Availability

The data that support the findings of this study are available from the corresponding author upon reasonable request.

## Supplementary Materials

The following are available online at https://www.mdpi.com/1422-0067/22/5/2285/s1. Figure S1: Proposed reaction mechanism based on the Chan-Evans-Lam coupling for the copper-mediated radiofluorination of an aryl boronic pinacol ester acid precursor, Figure S2-45: 1H-NMR and LC-MS of **PPY1**-**PPY22**.
