# Supplementary material for: Development of 18F-Labeled Radiotracers for PET Imaging of the Adenosine A2A Receptor: Synthesis, Radiolabeling and Preliminary Biological Evaluation
Source: Int J Mol Sci. 2021 Feb 25;22(5):2285. doi: 10.3390/ijms22052285 (PMC7956753; doi:10.3390/ijms22052285)
Supplement: Supplementary file 1 [file ijms-22-02285-s001.pdf]

International Journal of *Molecular Sciences*

MDPI

Supporting Information

Development of ^{18}F-Labeled Radiotracers for PET Imaging of the Adenosine A2A Receptor: Synthesis, Radiolabeling and Preliminary Biological Evaluation

Thu Hang Lai [1,2,*], Susann Schröder [2], Magali Toussaint [1], Sladjana Dukić-Stefanović [1], Mathias Kranz [1,3,4], Friedrich-Alexander Ludwig [1], Steffen Fischer [1], Jörg Steinbach [1,2], Winnie Deuther-Conrad [1], Peter Brust [1], Rareş-Petru Moldovan [1,*]

1 Helmholtz-Zentrum Dresden-Rossendorf (HZDR), Department of Neuroradiopharmaceuticals, Institute of Radiopharmaceutical Cancer Research, Research site Leipzig, 04318 Leipzig, Germany; m.toussaint@hzdr.de (M.T.); s.dukic-stefanovic@hzdr.de (S.D.-S.); mathias.kranz@uit.no (M.K.); f.ludwig@hzdr.de (F.-A.L.); s.fischer@hzdr.de (S.F.); steinbach-joerg@web.de (J.S.); w.deuther-conrad@hzdr.de (W.D.-C.); p.brust@hzdr.de (P.B.)

2 Department of Research and Development, ROTOP Pharmaka Ltd., Dresden 01328, Germany; s.schroeder@hzdr.de

3 PET Imaging Center, University Hospital of North Norway (UNN), 9009 Tromsø, Norway

4 Nuclear Medicine and Radiation Biology Research Group, The Arctic University of Norway, 9009 Tromsø, Norway

* Correspondence: t.lai@hzdr.de (T.H.L.); r.moldovan@hzdr.de (R.-P.M.); Tel.: +49-341-234-179-4635 (T.H.L.); +49-341-234-179-4634 (R.-P.M.)

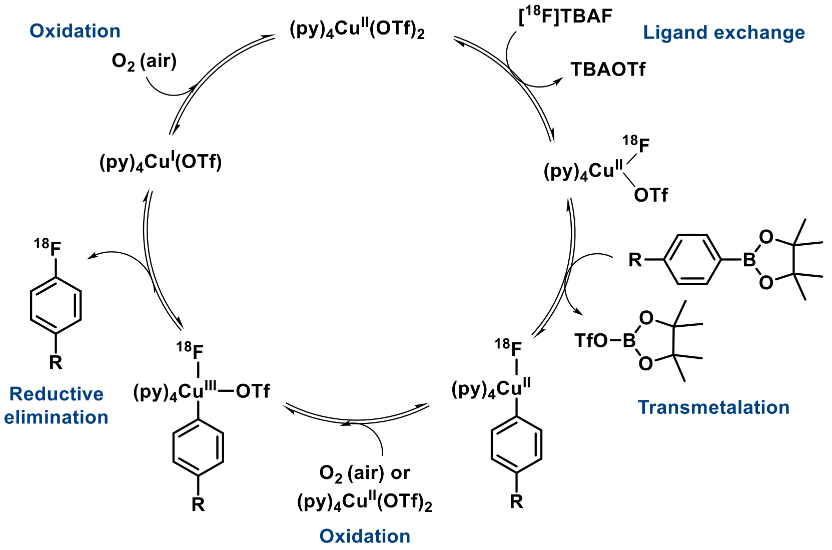

Figure S1: Proposed reaction mechanism based on the Chan-Evans-Lam coupling for the copper-mediated radiofluorination of an aryl boronic pinacol ester acid precursor.

1-(4-Fluorobenzyl)-4-(furan-2-yl)-1*H*-pyrazolo[3,4-*d*]pyrimidin-6-amine (PPY1)

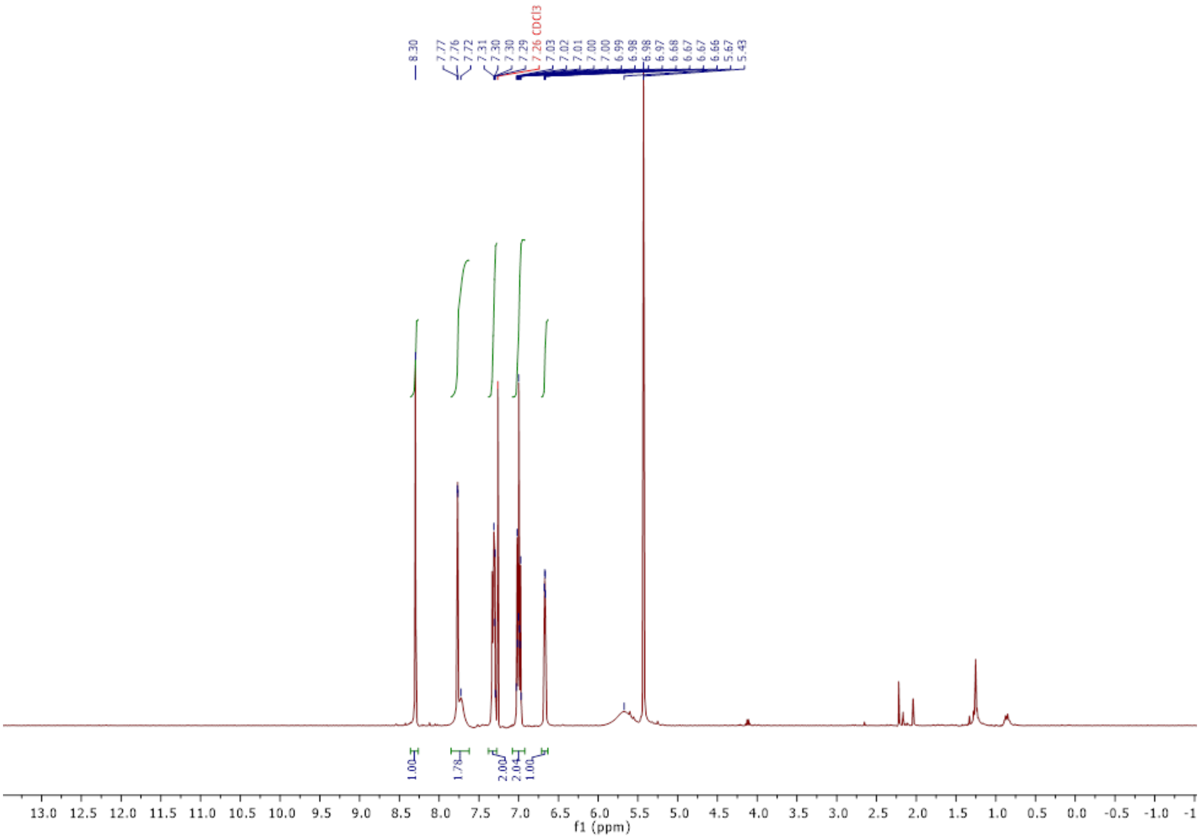

Figure S2: ^{1}H-NMR of **PPY1**.

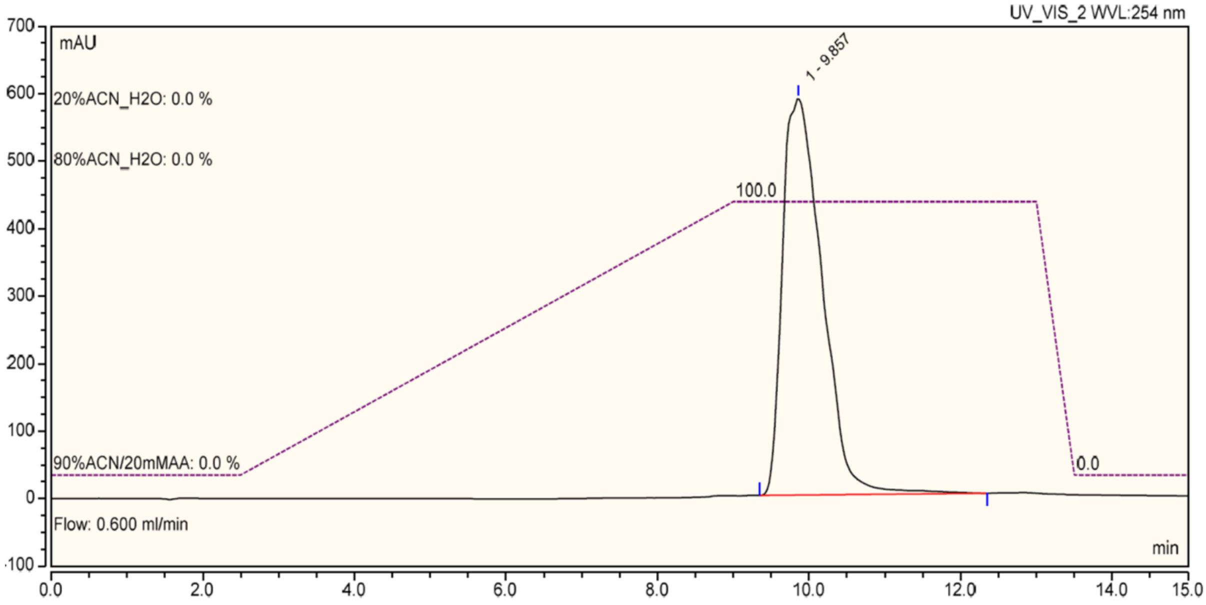

Figure S3: LC-MS chromatogram of **PPY1**.

1-(2-Fluorobenzyl)-4-(furan-2-yl)-1*H*-pyrazolo[3,4-*d*]pyrimidin-6-amine (PPY2)

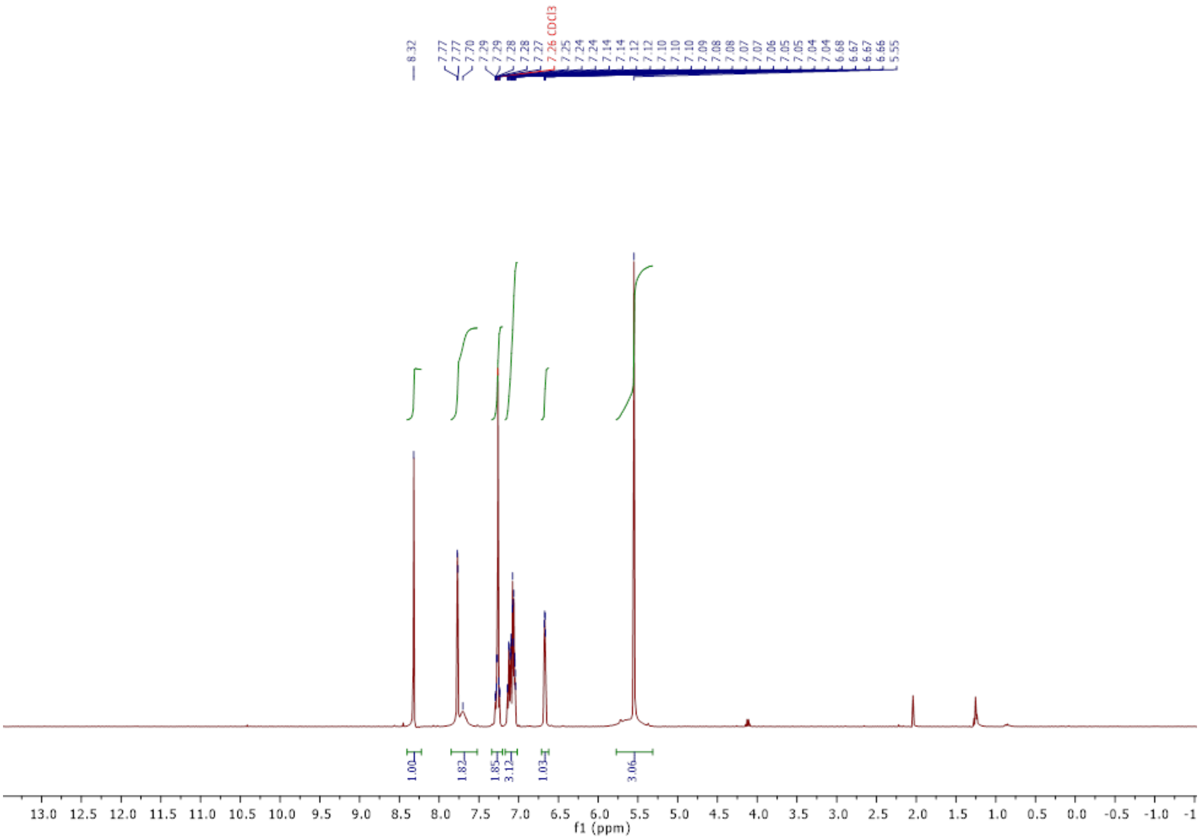

Figure S4: ^{1}H-NMR of **PPY2**.

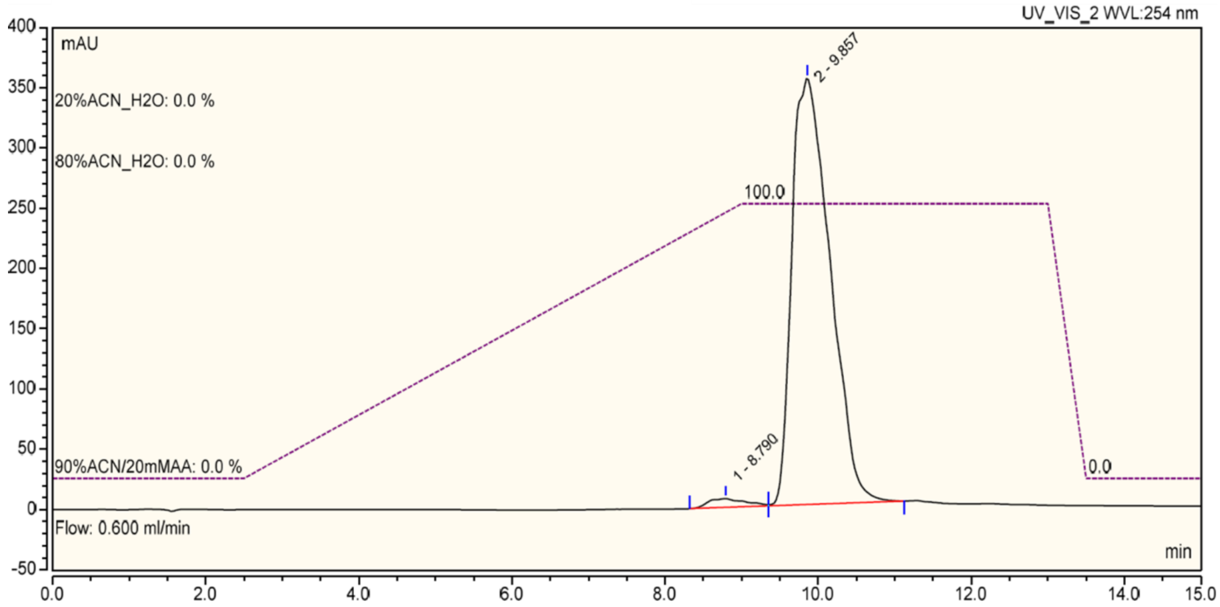

Figure S5: LC-MS chromatogram of **PPY2**.

1-(3-Fluorobenzyl)-4-(furan-2-yl)-1*H*-pyrazolo[3,4-*d*]pyrimidin-6-amin (PPY3)

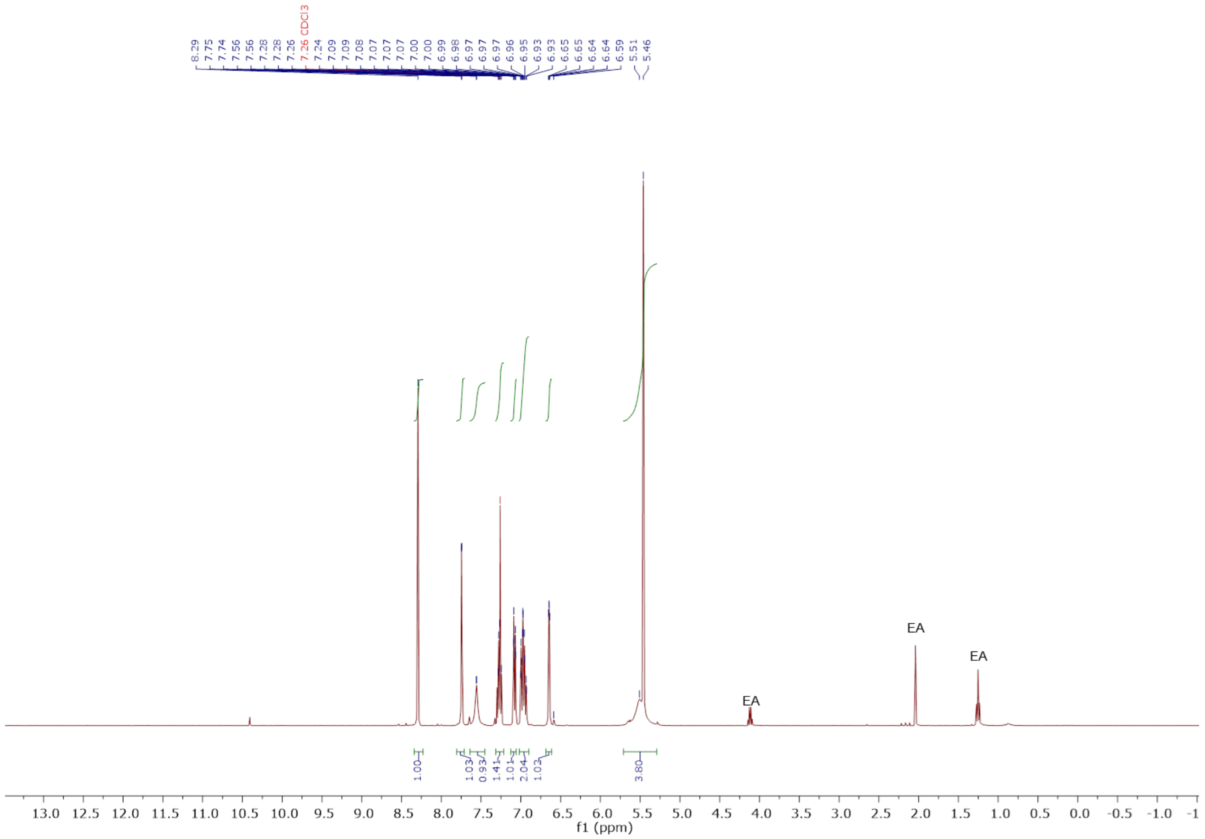

Figure S6: ^{1}H-NMR of **PPY3**.

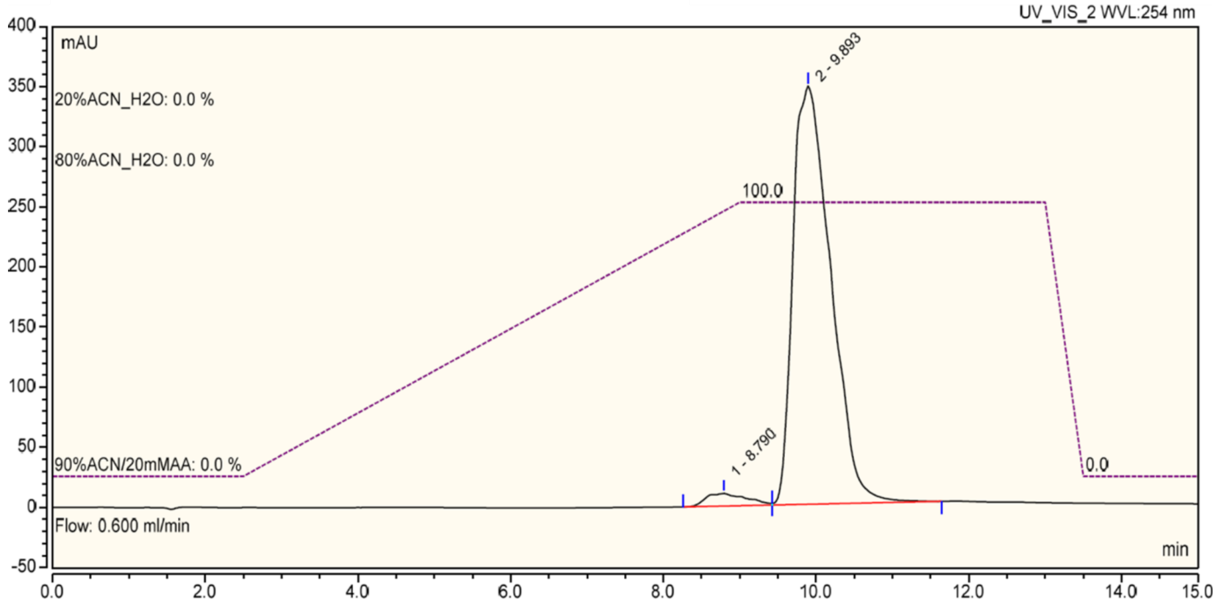

Figure S7: LC-MS chromatogram of **PPY3**.

1-((2-Fluoropyridin-3-yl)methyl)-4-(furan-2-yl)-1*H*-pyrazolo[3,4-*d*]pyrimidin-6-amine (PPY4)

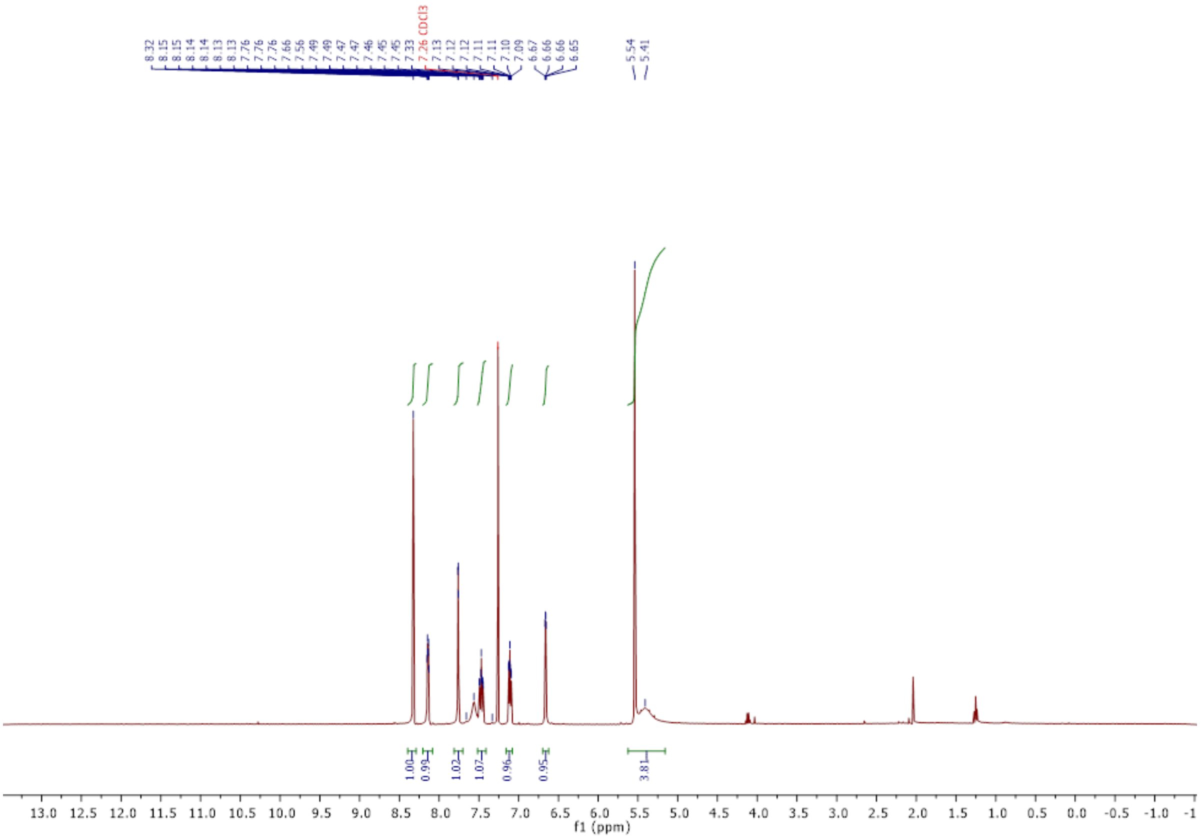

Figure S8: ^{1}H-NMR of **PPY4**.

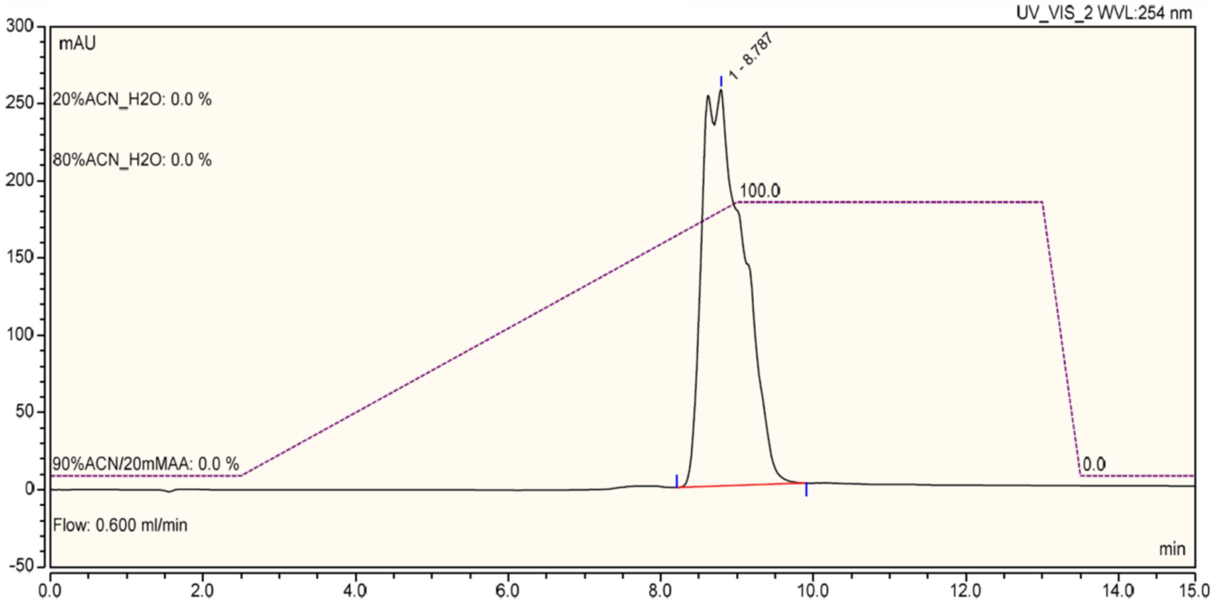

Figure S9: LC-MS chromatogram of **PPY4**.

1-((6-Fluoropyridin-3-yl)methyl)-4-(furan-2-yl)-1*H*-pyrazolo[3,4-*d*]pyrimidin-6-amine (PPY5)

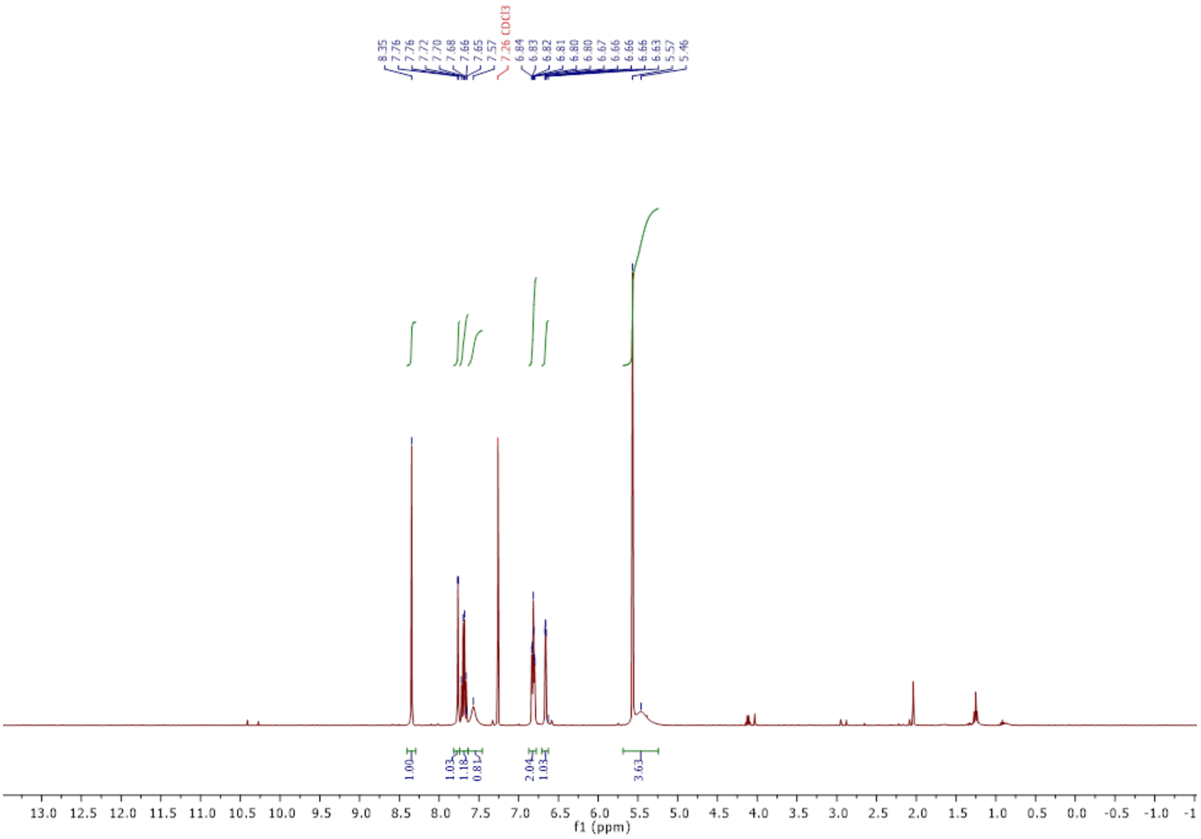

Figure S10: ^{1}H-NMR of **PPY5**.

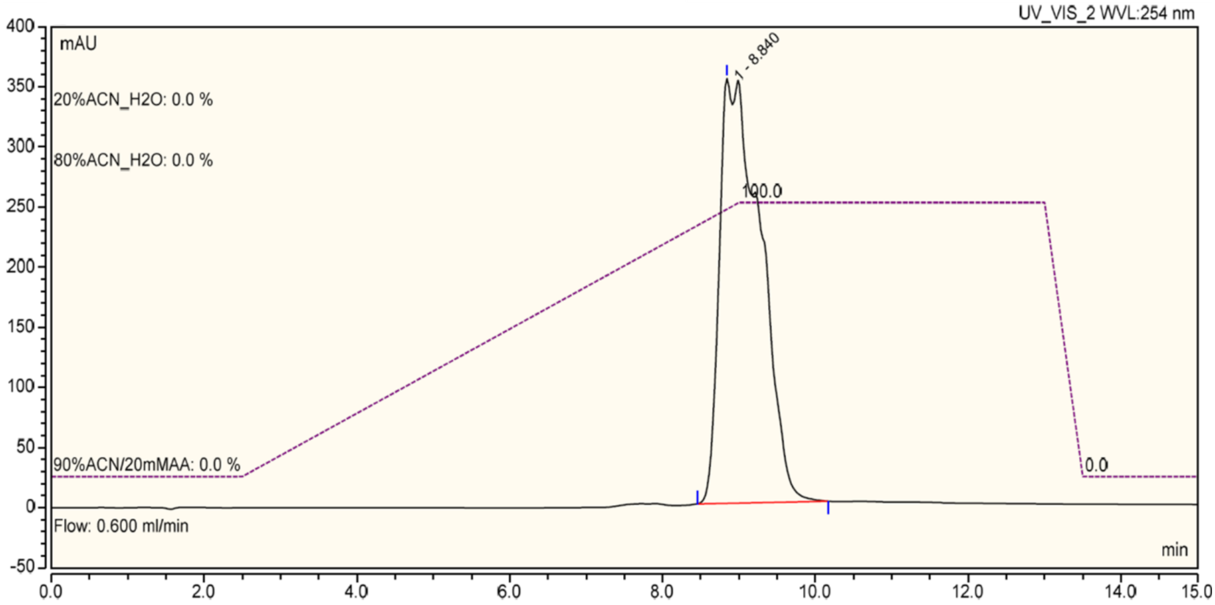

Figure S11: LC-MS chromatogram of **PPY5**.

1-((6-Fluoropyridin-2-yl)methyl)-4-(furan-2-yl)-1*H*-pyrazolo[3,4-*d*]pyrimidin-6-amine (PPY6)

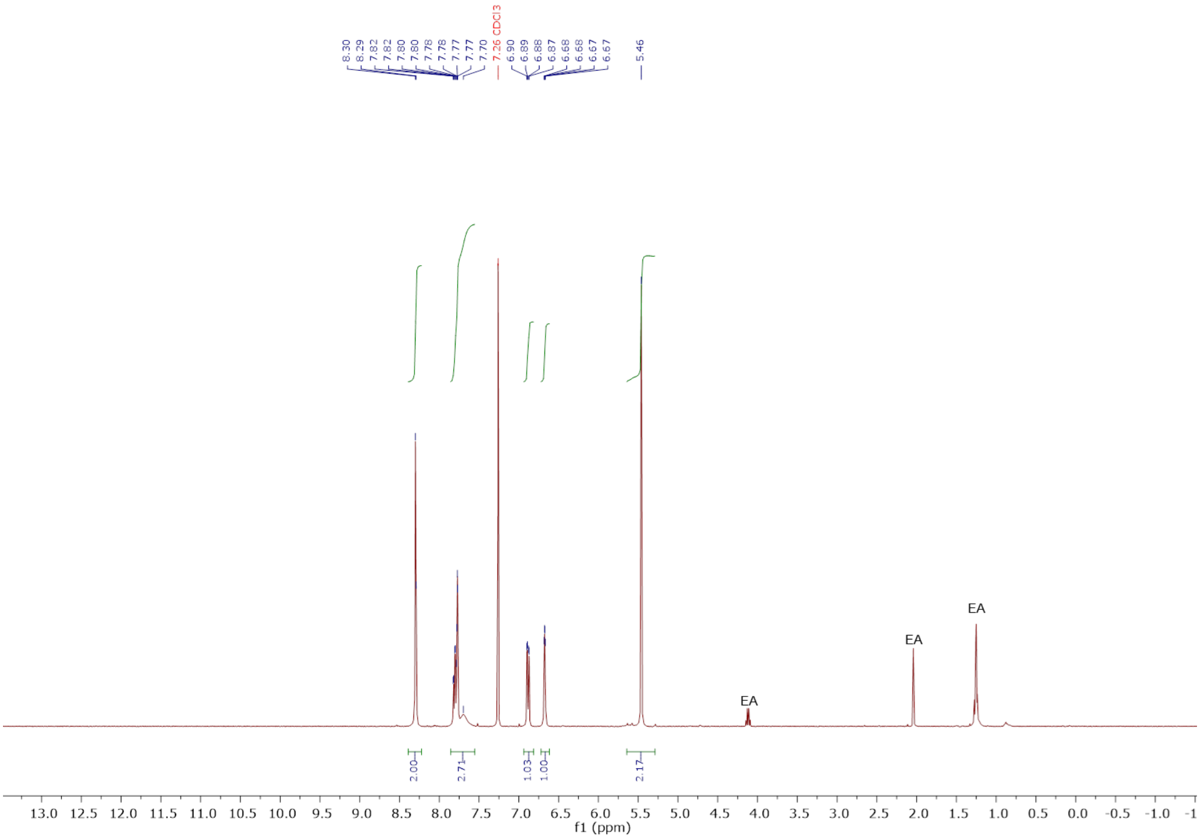

Figure S12: ^{1}H-NMR of **PPY6**.

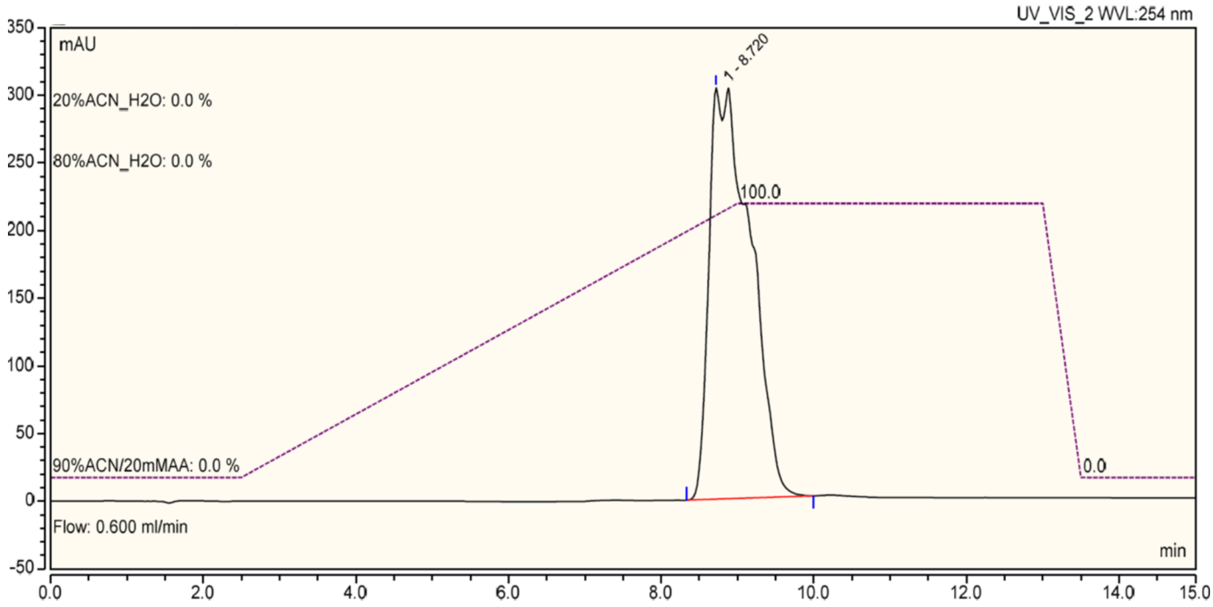

Figure S13: LC-MS chromatogram of **PPY6**.

1-((2-Fluoropyridin-4-yl)methyl)-4-(furan-2-yl)-1*H*-pyrazolo[3,4-*d*]pyrimidin-6-amine (PPY7)

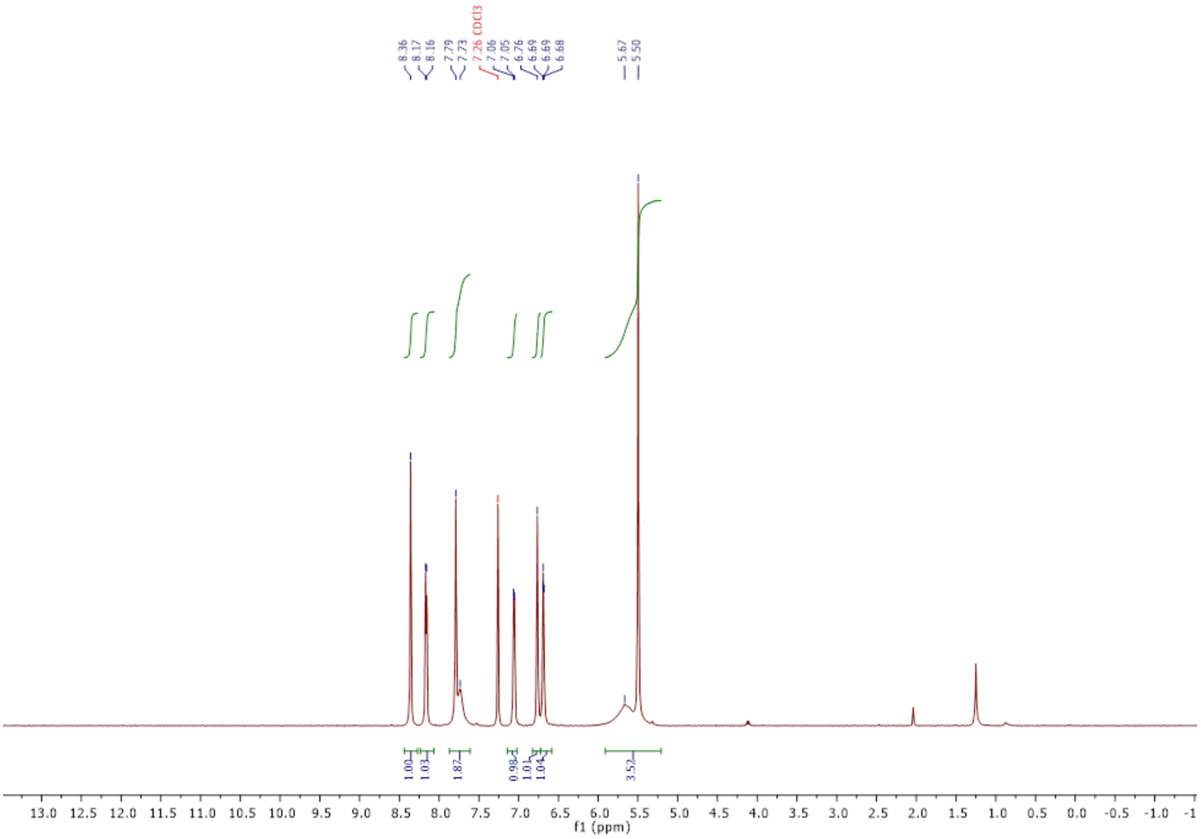

Figure S14: ^{1}H-NMR of **PPY7**.

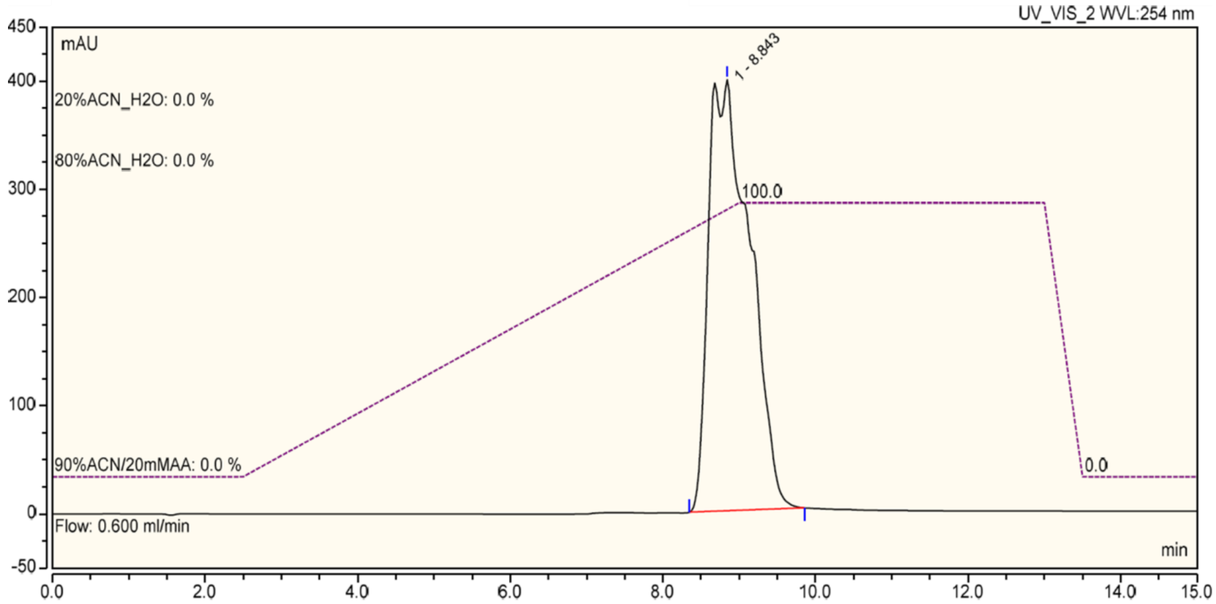

Figure S15: LC-MS chromatogram of **PPY7**.

1-(4-Bromo-2-fluorobenzyl)-4-(furan-2-yl)-1*H*-pyrazolo[3,4-*d*]pyrimidin-6-amine (PPY8)

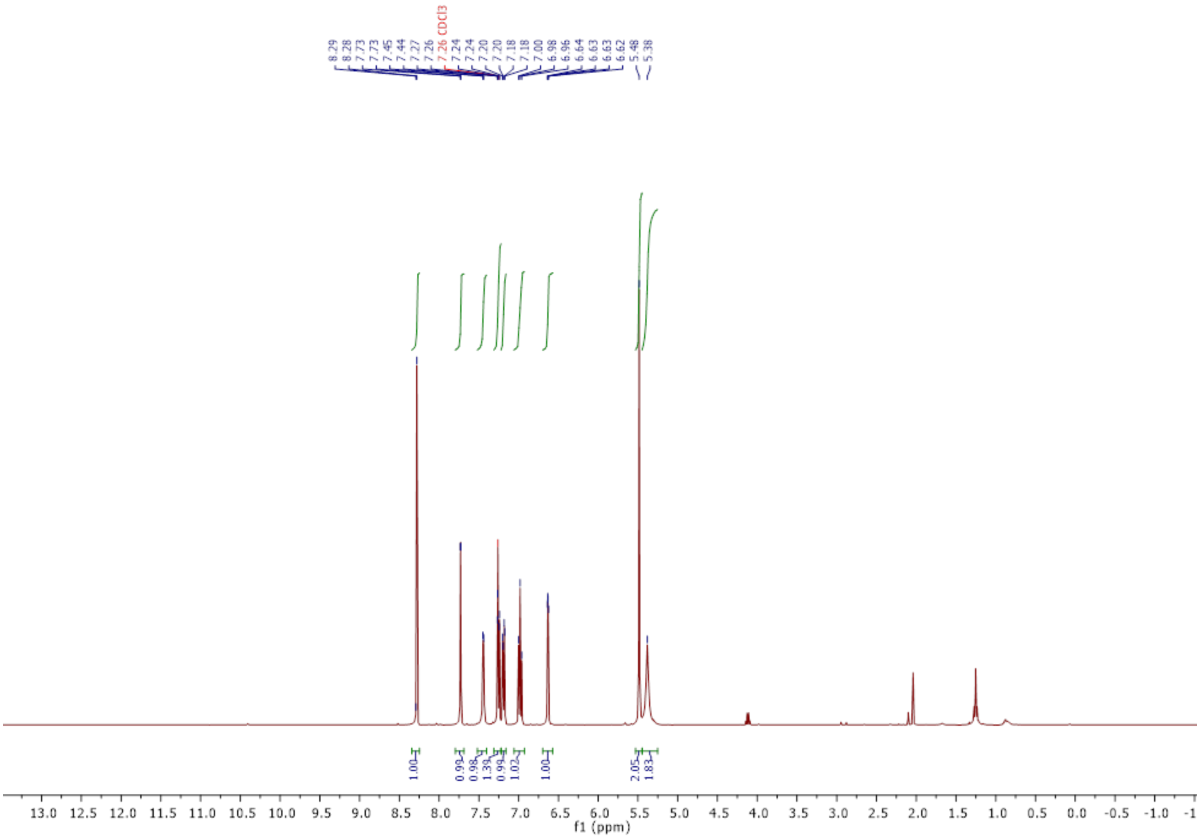

Figure S16: ^{1}H-NMR of **PPY8**.

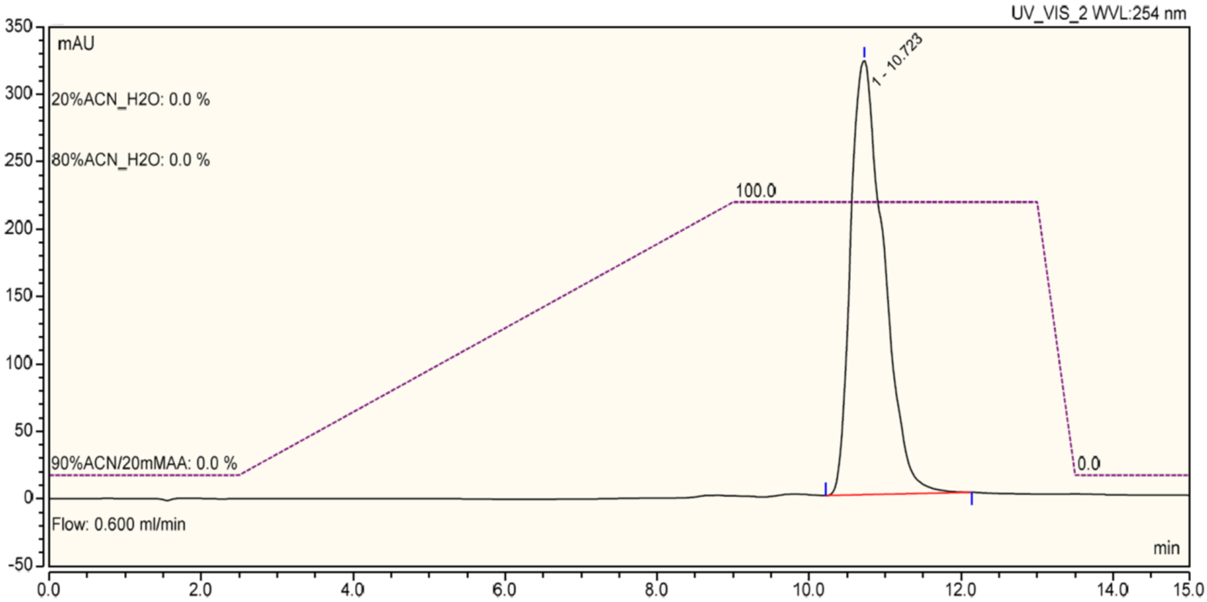

Figure S17: LC-MS chromatogram of **PPY8**.

1-(2-Bromo-4-fluorobenzyl)-4-(furan-2-yl)-1*H*-pyrazolo[3,4-*d*]pyrimidin-6-amine (PPY9)

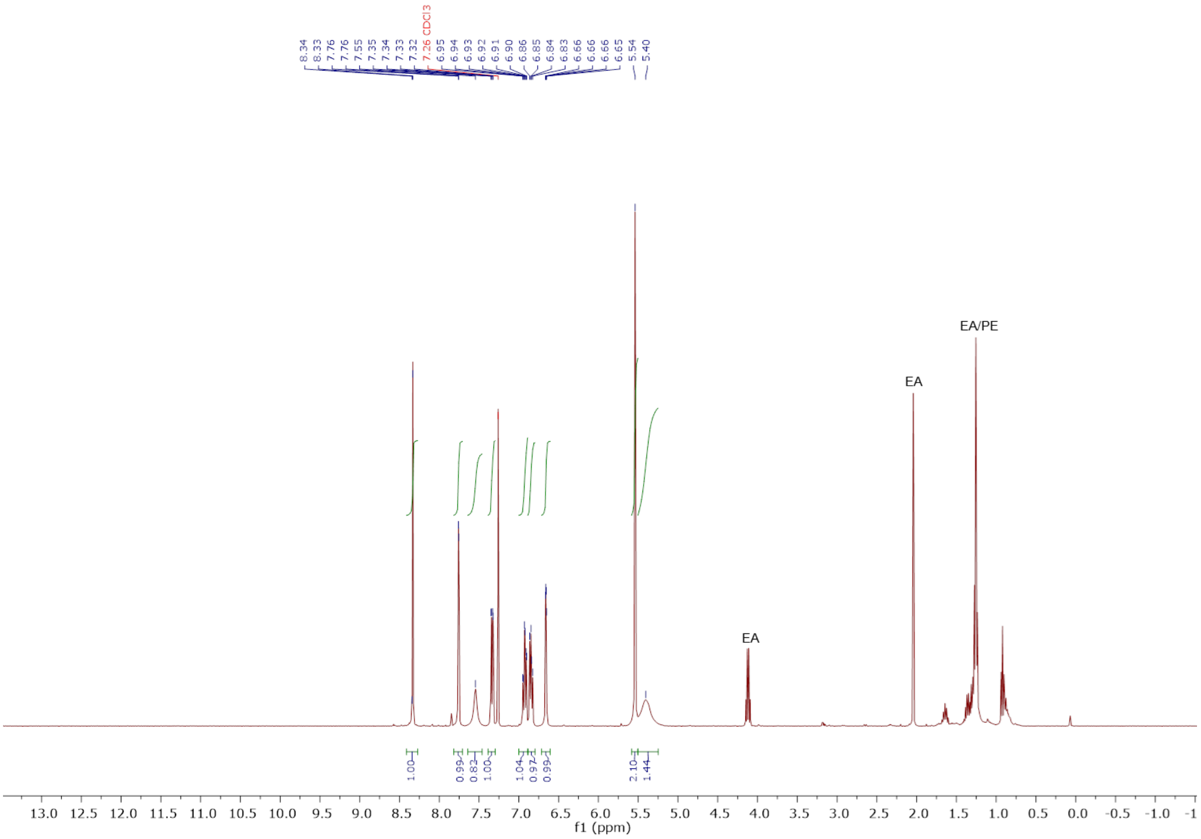

Figure S18: ^{1}H-NMR of **PPY9**.

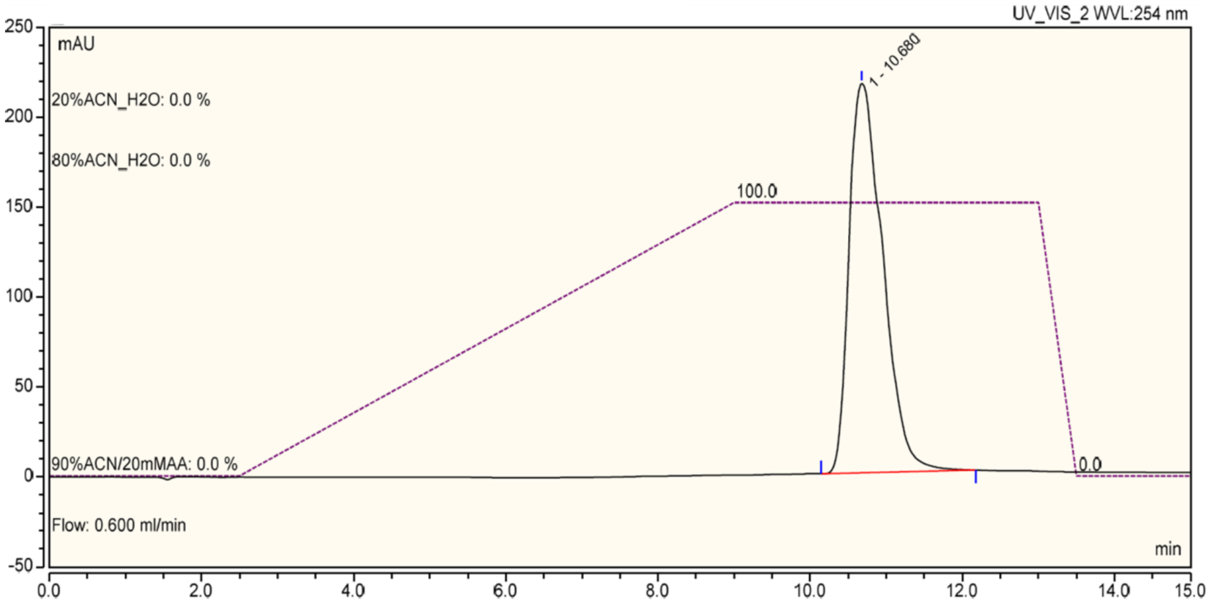

Figure S19: LC-MS chromatogram of **PPY9**.

1-(3-Bromo-5-fluorobenzyl)-4-(furan-2-yl)-1*H*-pyrazolo[3,4-*d*]pyrimidin-6-amine (PPY10)

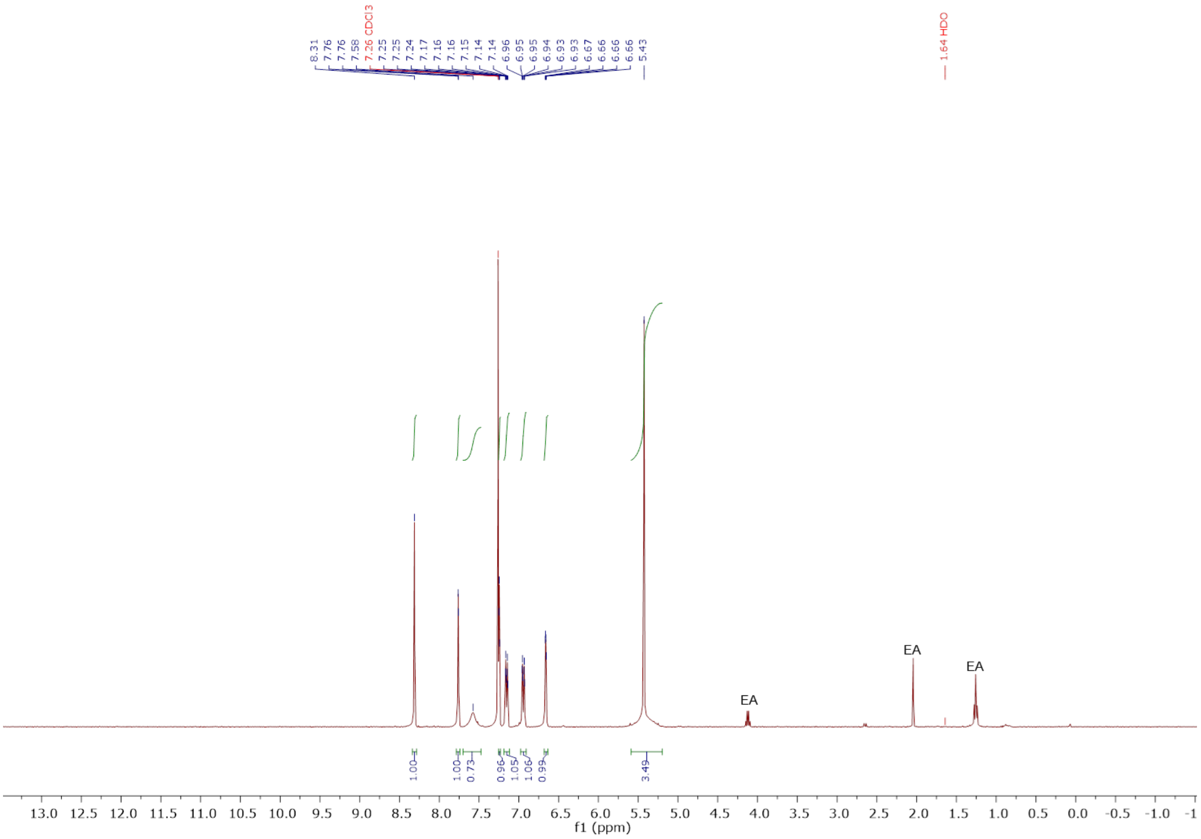

Figure S20: ^{1}H-NMR of **PPY10**.

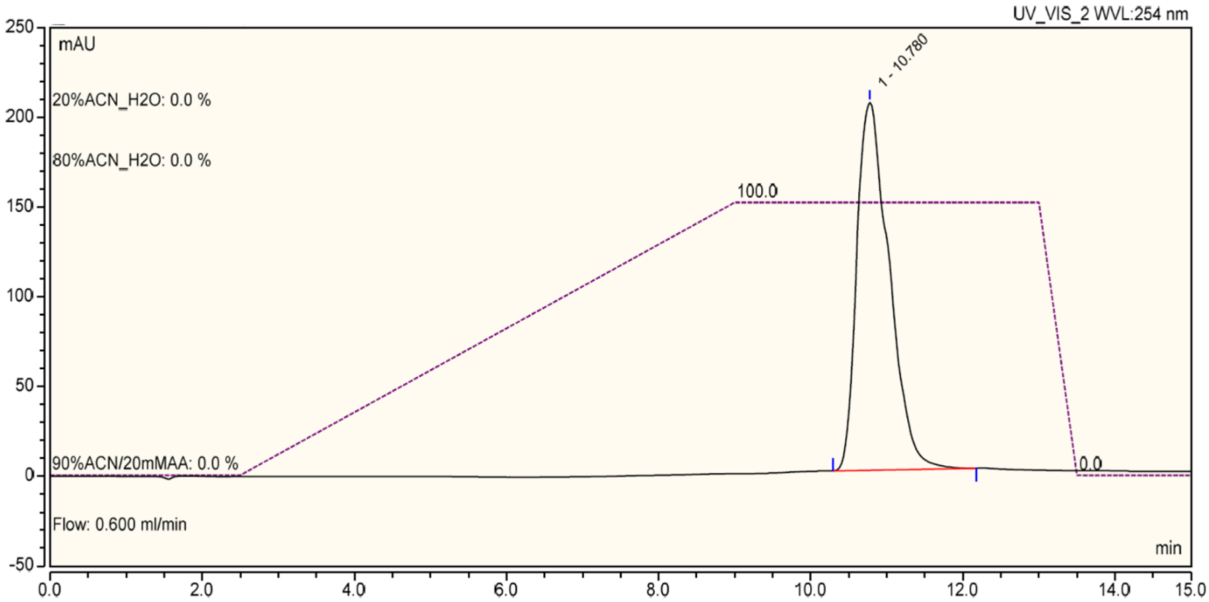

Figure S21: LC-MS chromatogram of **PPY10**.

1-(2-Bromo-6-fluorobenzyl)-4-(furan-2-yl)-1*H*-pyrazolo[3,4-*d*]pyrimidin-6-amin (PPY11)

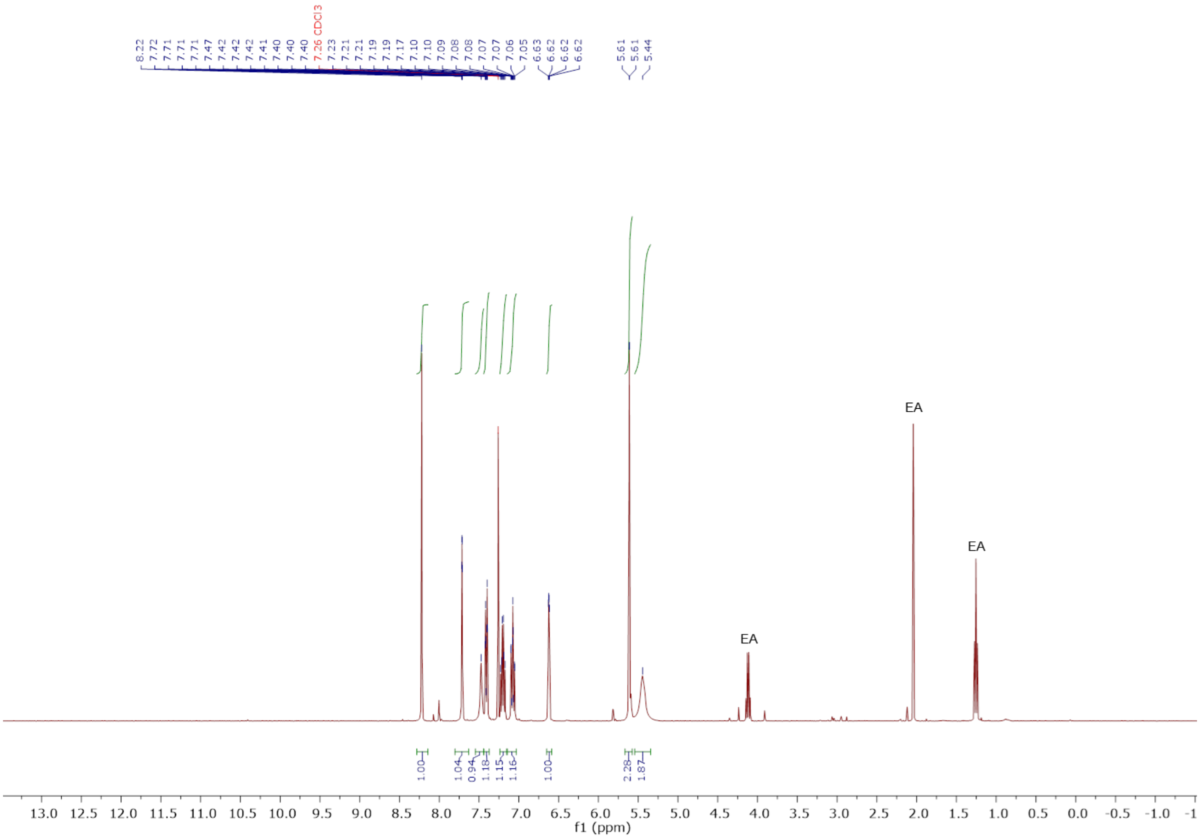

Figure S22: ^{1}H-NMR of **PPY11**.

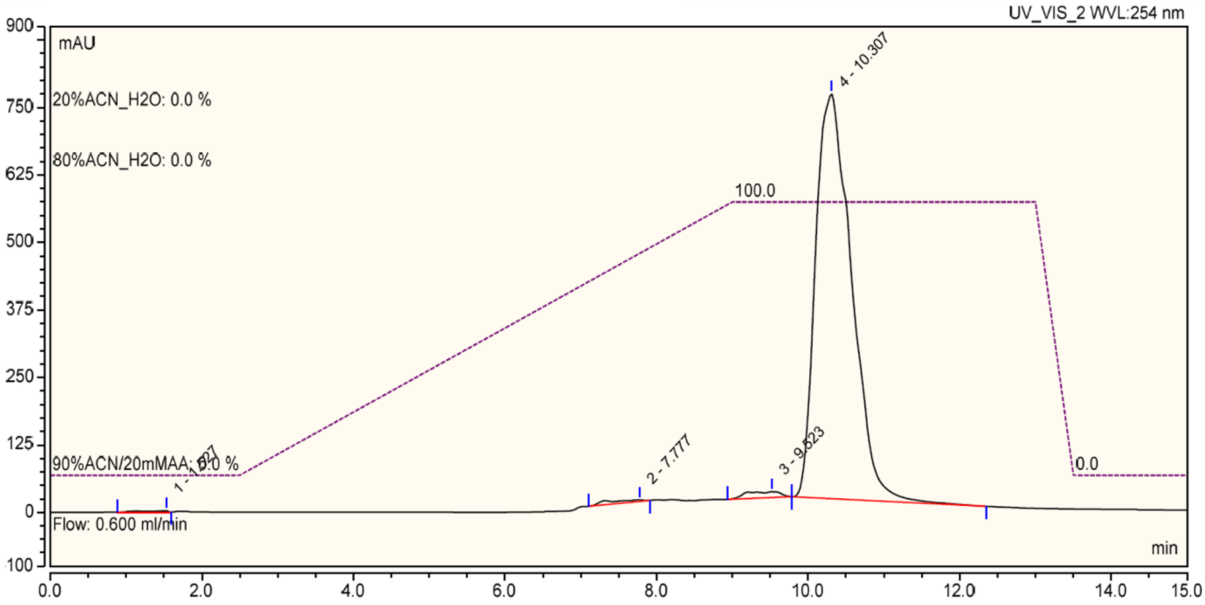

Figure S23: LC-MS chromatogram of **PPY11**.

2-((6-Amino-4-(furan-2-yl)-1*H*-pyrazolo[3,4-*d*]pyrimidin-1-yl)methyl)-5-fluoro-benzonitrile (PPY12)

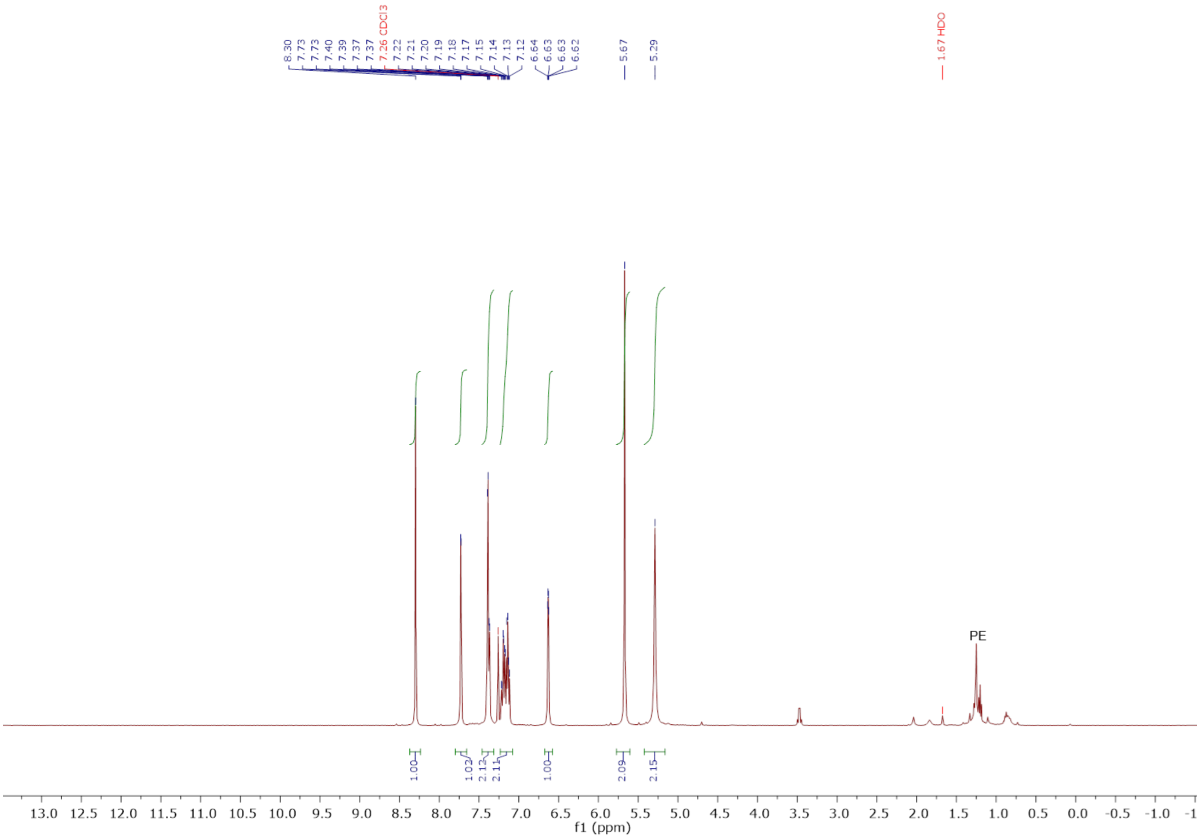

Figure S24: ¹H-NMR of **PPY12**.

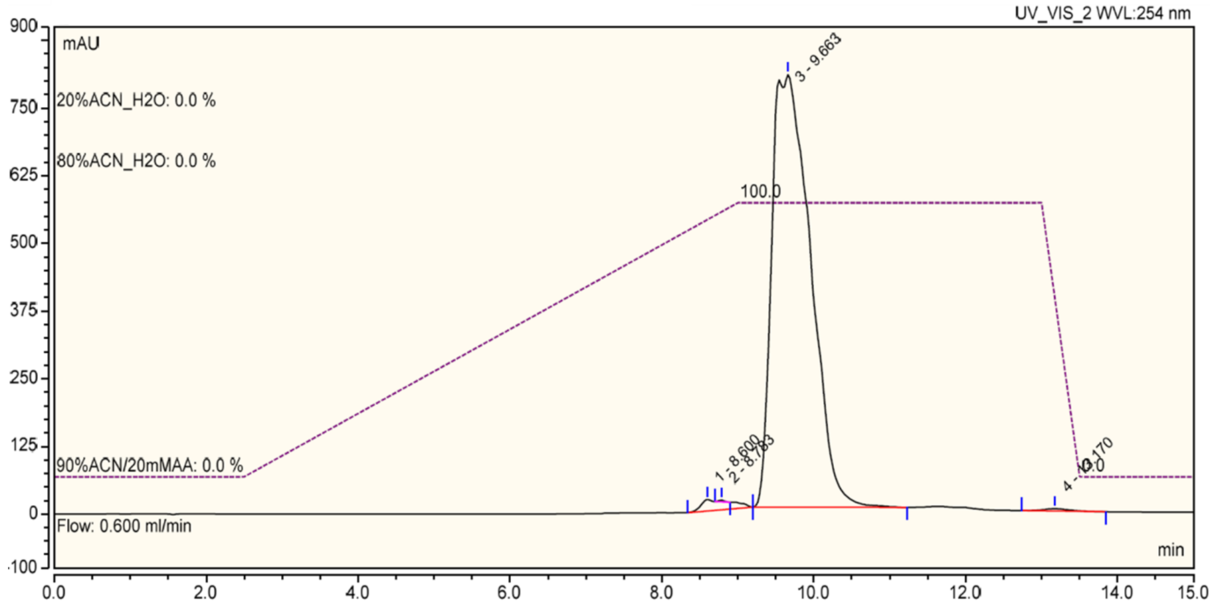

Figure S25: LC-MS chromatogram of **PPY12**.

4-((6-Amino-4-(furan-2-yl)-1*H*-pyrazolo[3,4-*d*]pyrimidin-1-yl)methyl)-3-fluoro-benzonitrile (PPY13)

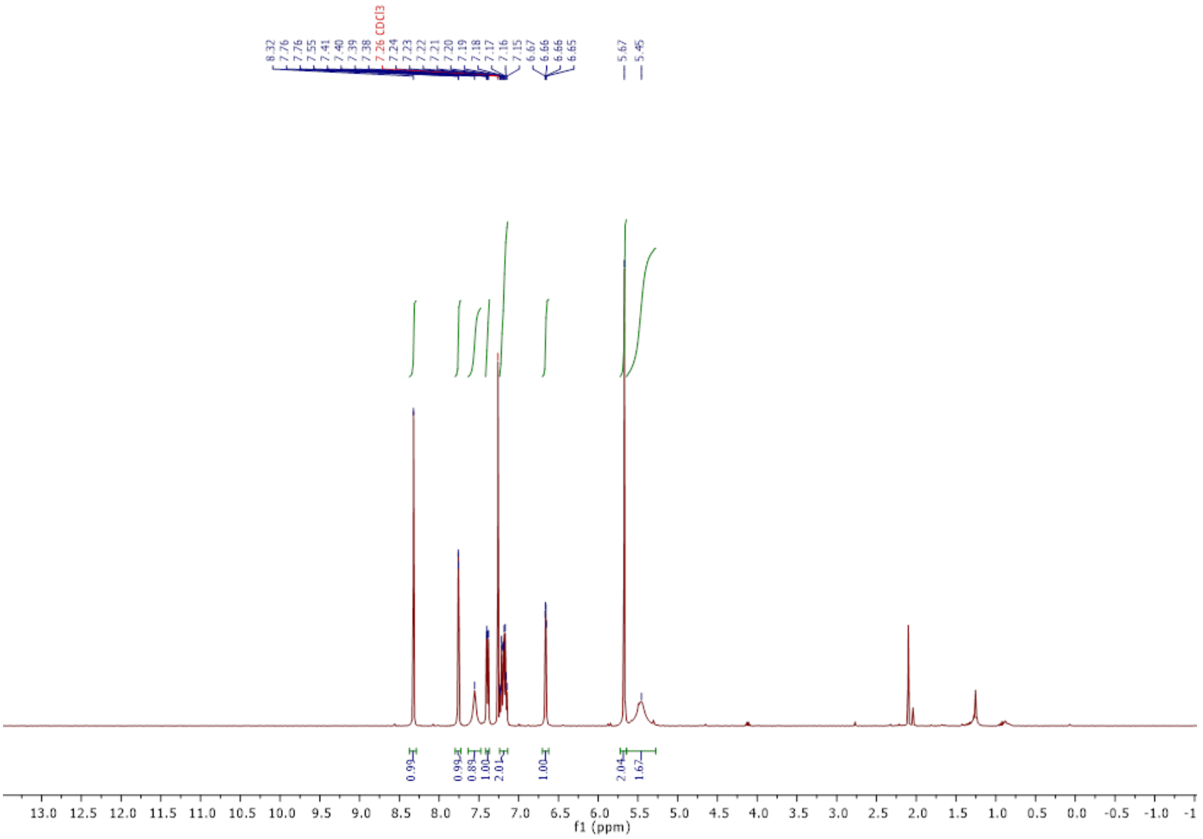

Figure S26: ^{1}H-NMR of **PPY13**.

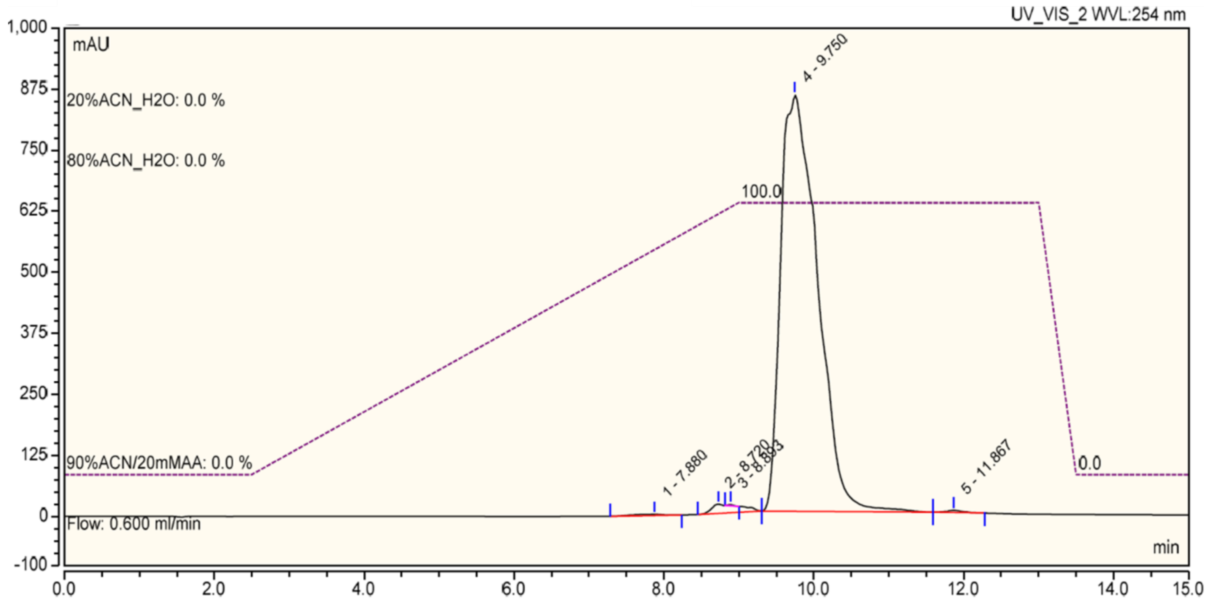

Figure S27: LC-MS chromatogram of **PPY13**.

3-((6-Amino-4-(furan-2-yl)-1*H*-pyrazolo[3,4-*d*]pyrimidin-1-yl)methyl)-5-fluoro-benzonitrile (PPY14)

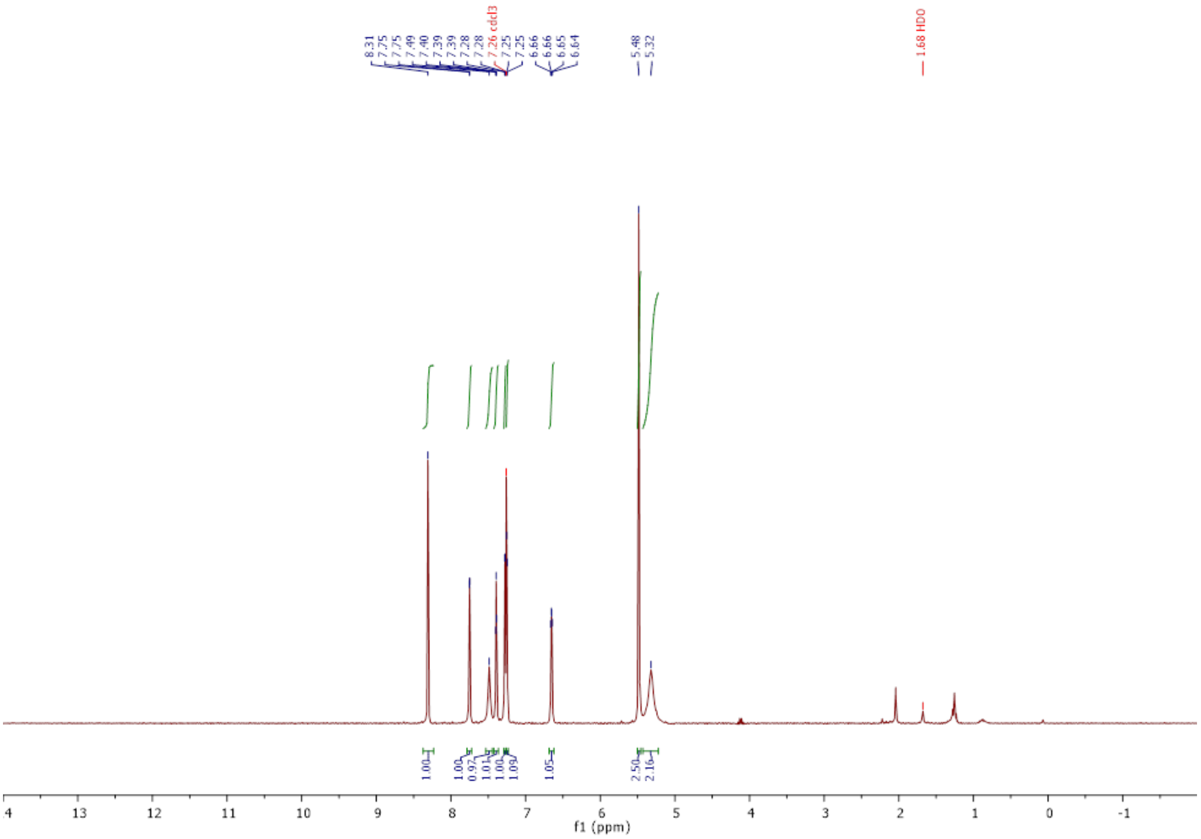

Figure S28: ^{1}H-NMR of **PPY14**.

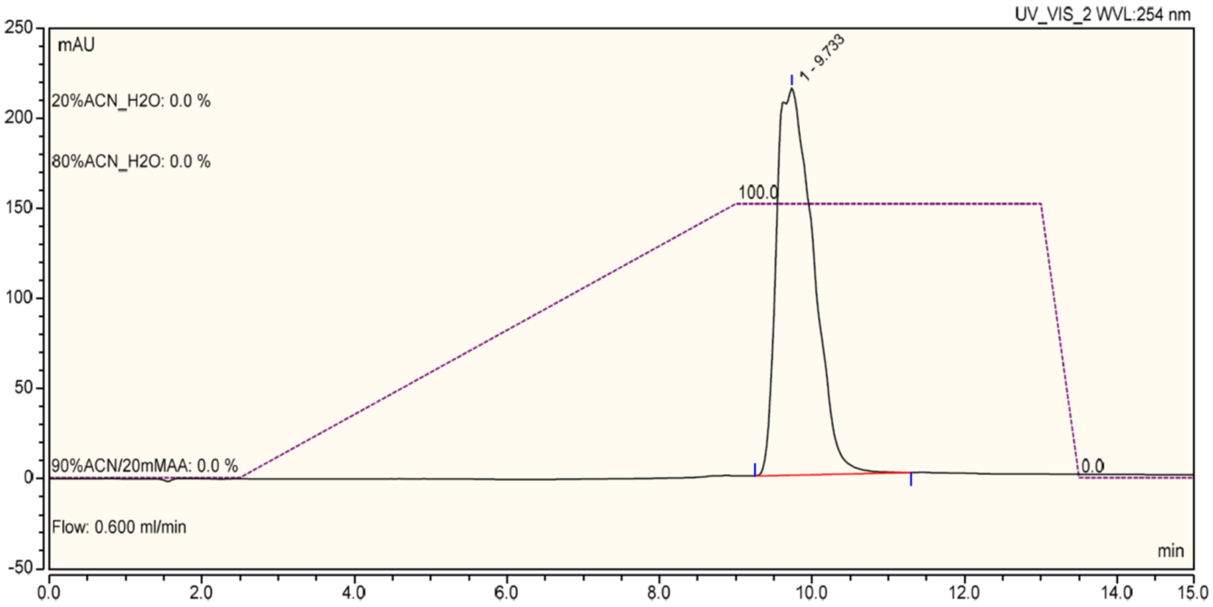

Figure S29: LC-MS chromatogram of **PPY14**.

2-((6-Amino-4-(furan-2-yl)-1*H*-pyrazolo[3,4-*d*]pyrimidin-1-yl)methyl)-3-fluoro-benzonitrile (PPY15)

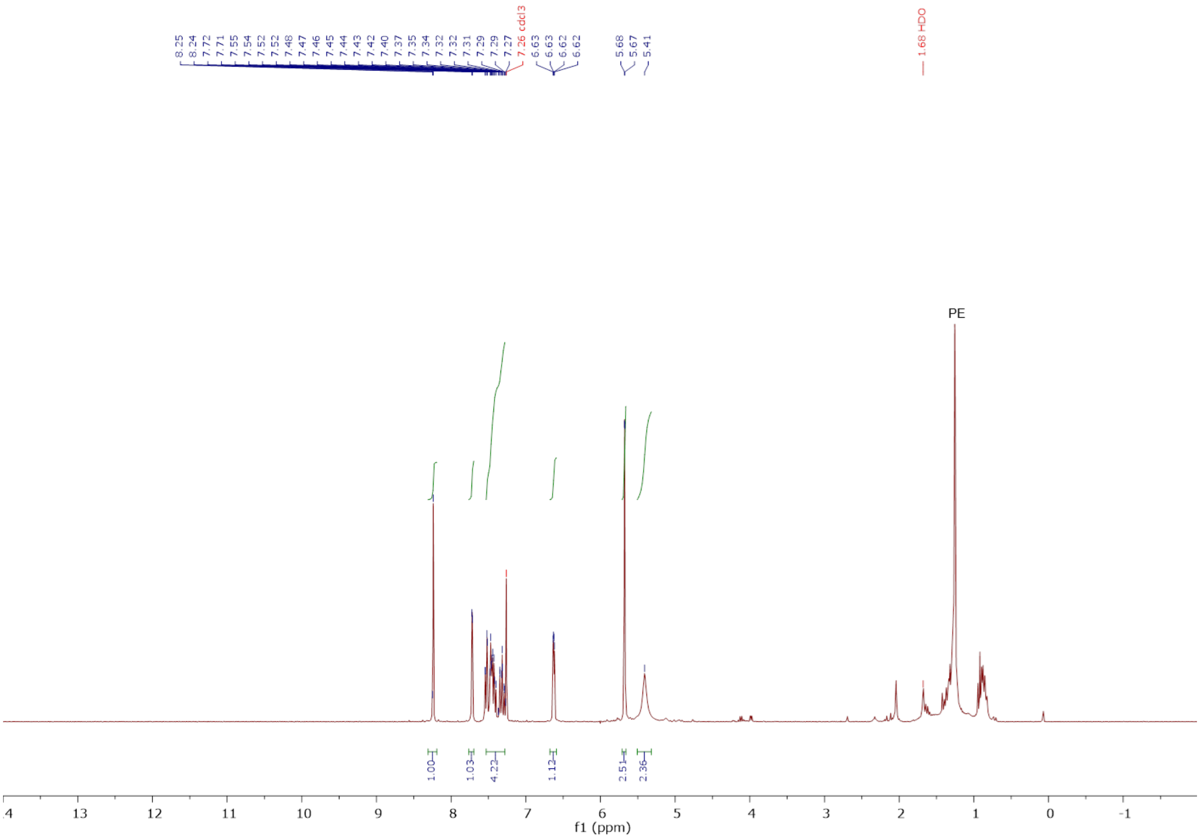

Figure S30: ^{1}H-NMR of **PPY15**.

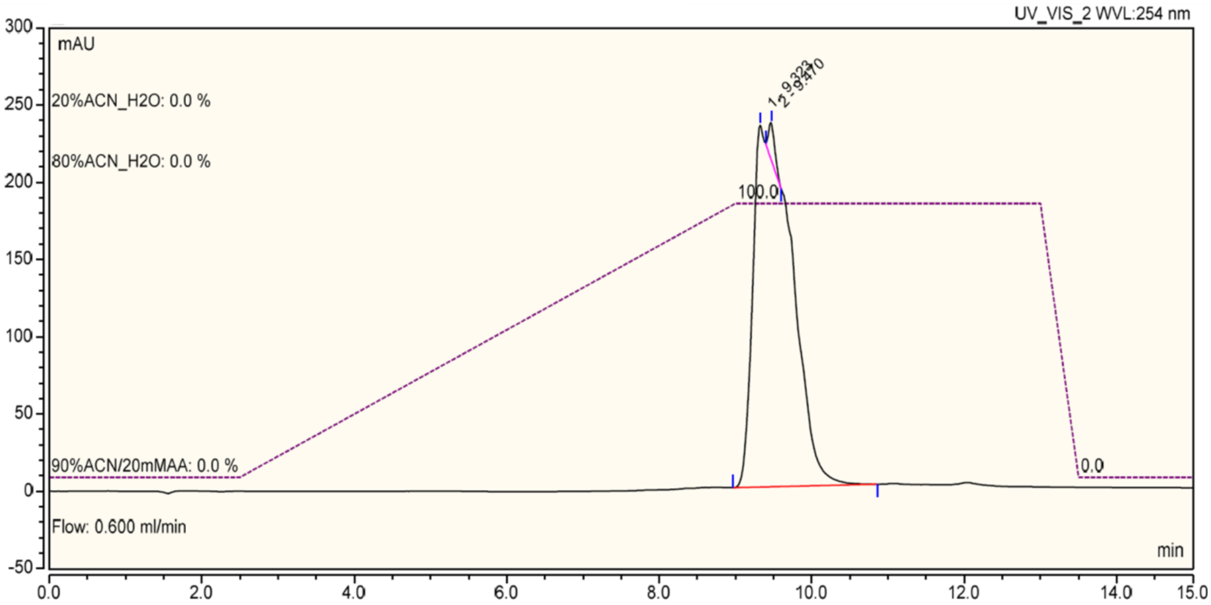

Figure S31: LC-MS chromatogram of **PPY15**.

1-(3-(2-Fluoroethyl)benzyl)-4-(furan-2-yl)-1*H*-yrazolo[3,4-*d*]pyrimidin-6-amine (PPY16)

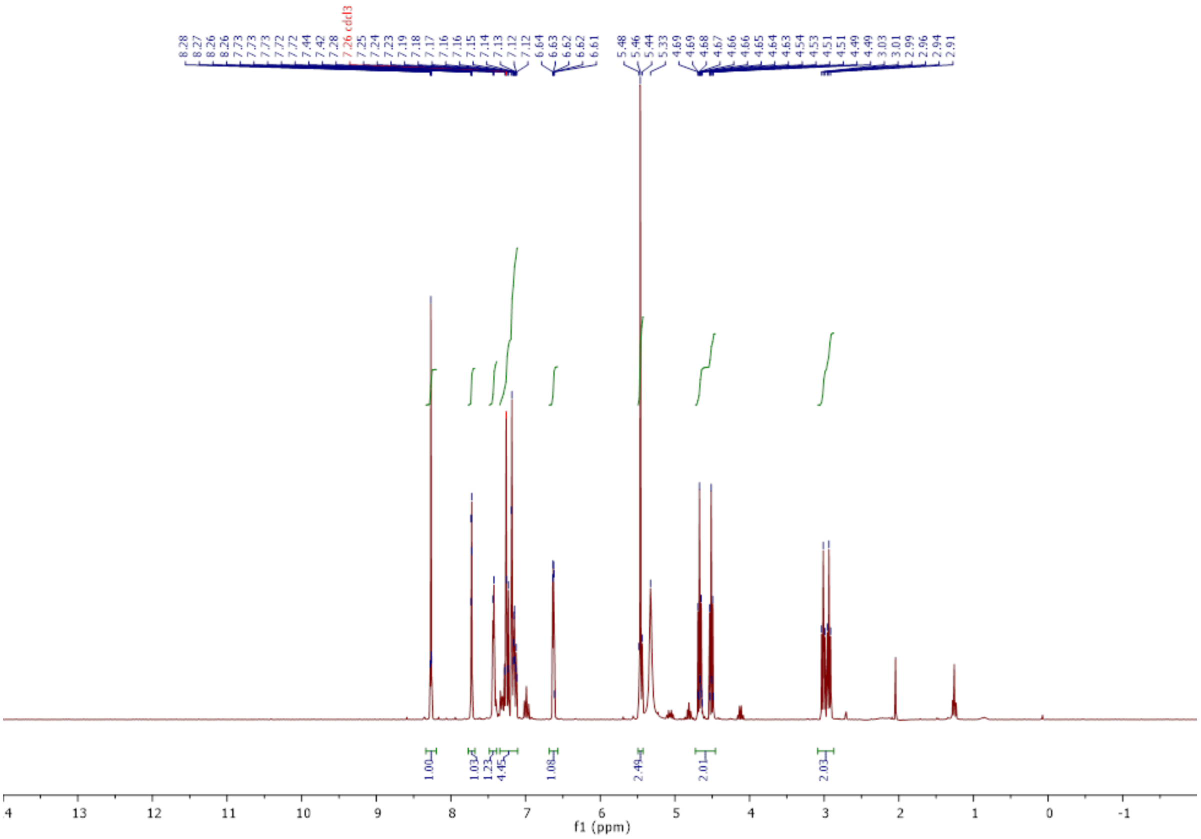

Figure S32: ^{1}H-NMR of **PPY16**.

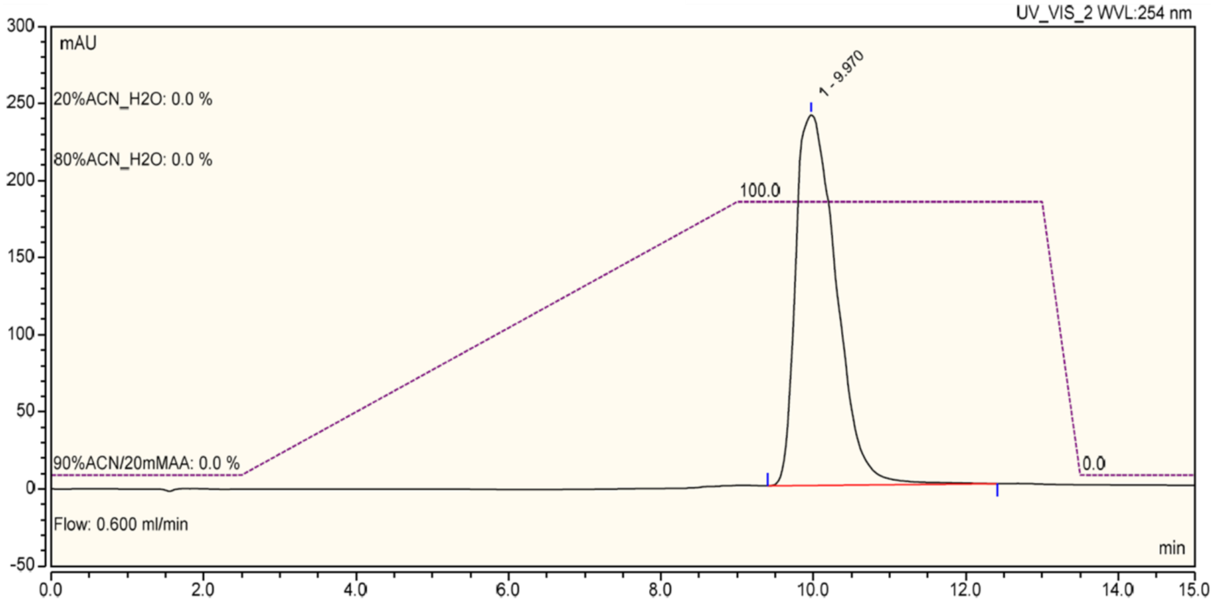

Figure S33: LC-MS chromatogram of **PPY16**.

1-(3-(2-Fluoroethoxy)benzyl)-4-(furan-2-yl)-1*H*-pyrazolo[3,4-*d*]pyrimidin-6-amine (PPY17)

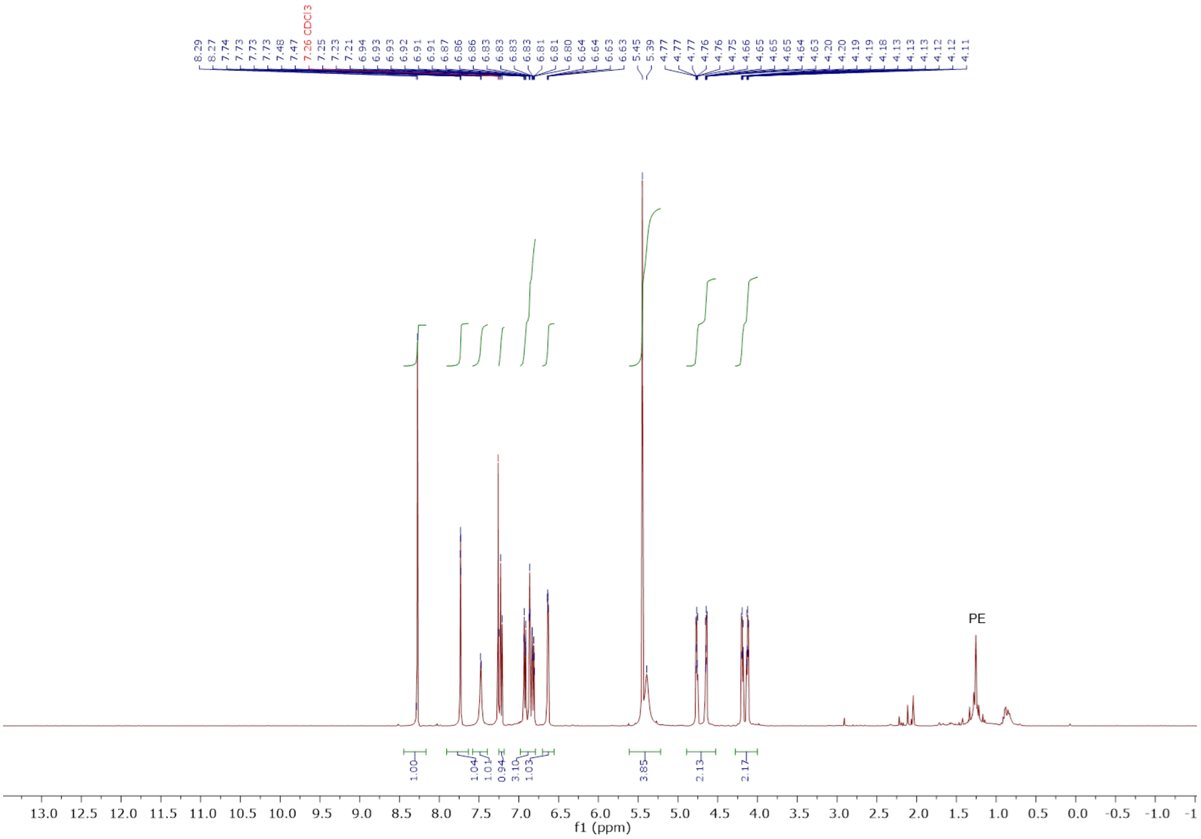

Figure S34: ^{1}H-NMR of **PPY17**.

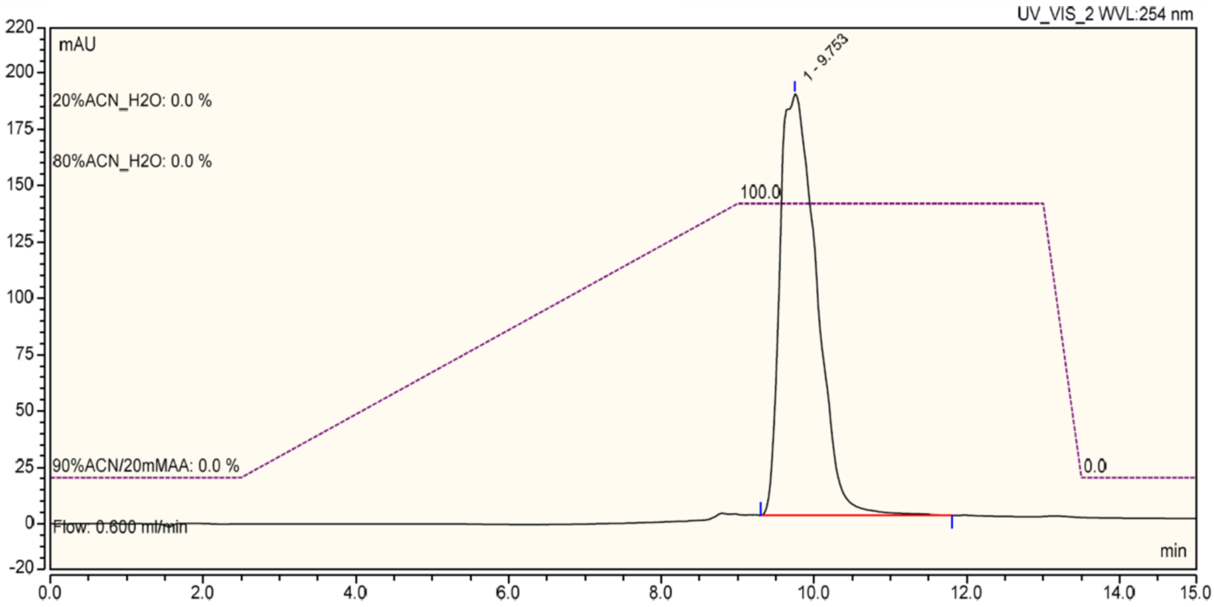

Figure S35: LC-MS chromatogram of **PPY17**.

1-(3-(3-Fluoropropoxy)benzyl)-4-(furan-2-yl)-1*H*-pyrazolo[3,4-*d*]pyrimidin-6-amine (PPY18)

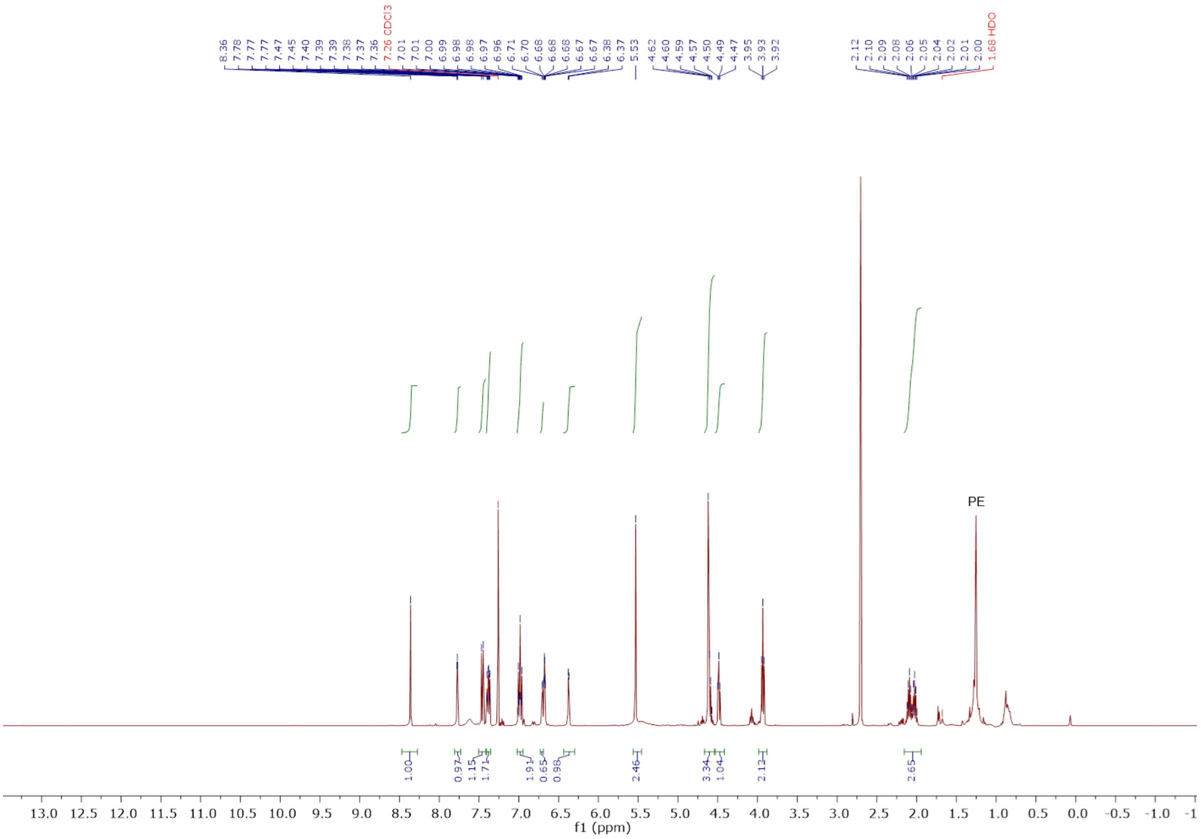

Figure S36: ^{1}H-NMR of **PPY18**.

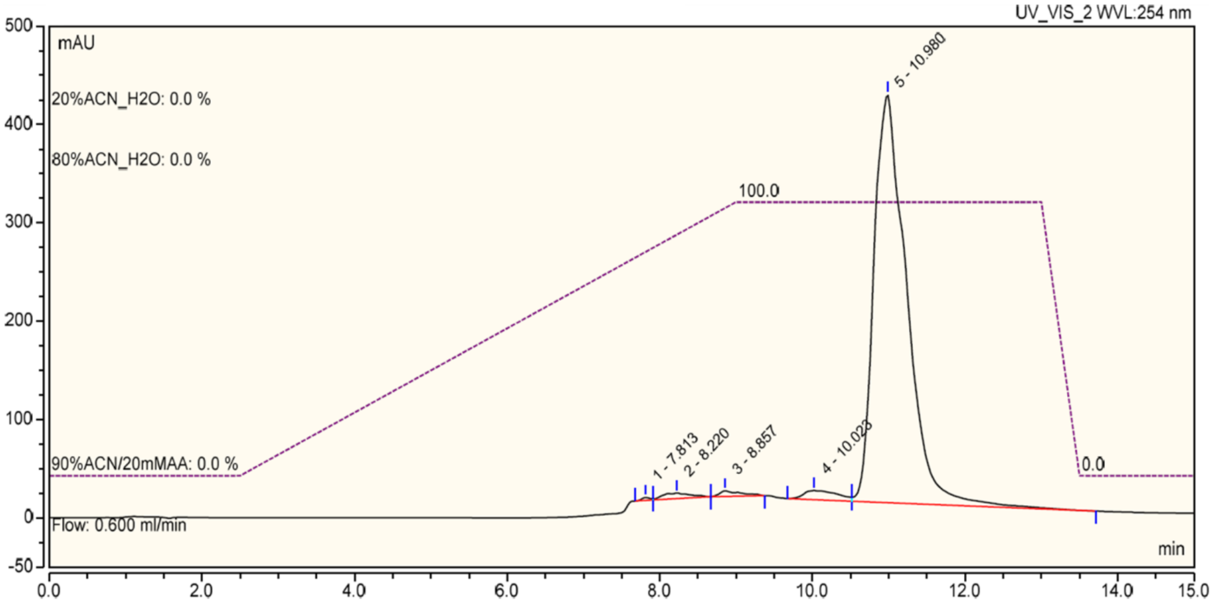

Figure S37: LC-MS chromatogram of **PPY18**.

1-(2-Fluorophenethyl)-4-(furan-2-yl)-1*H*-pyrazolo[3,4-*d*]pyrimidin-6-amine (PPY19)

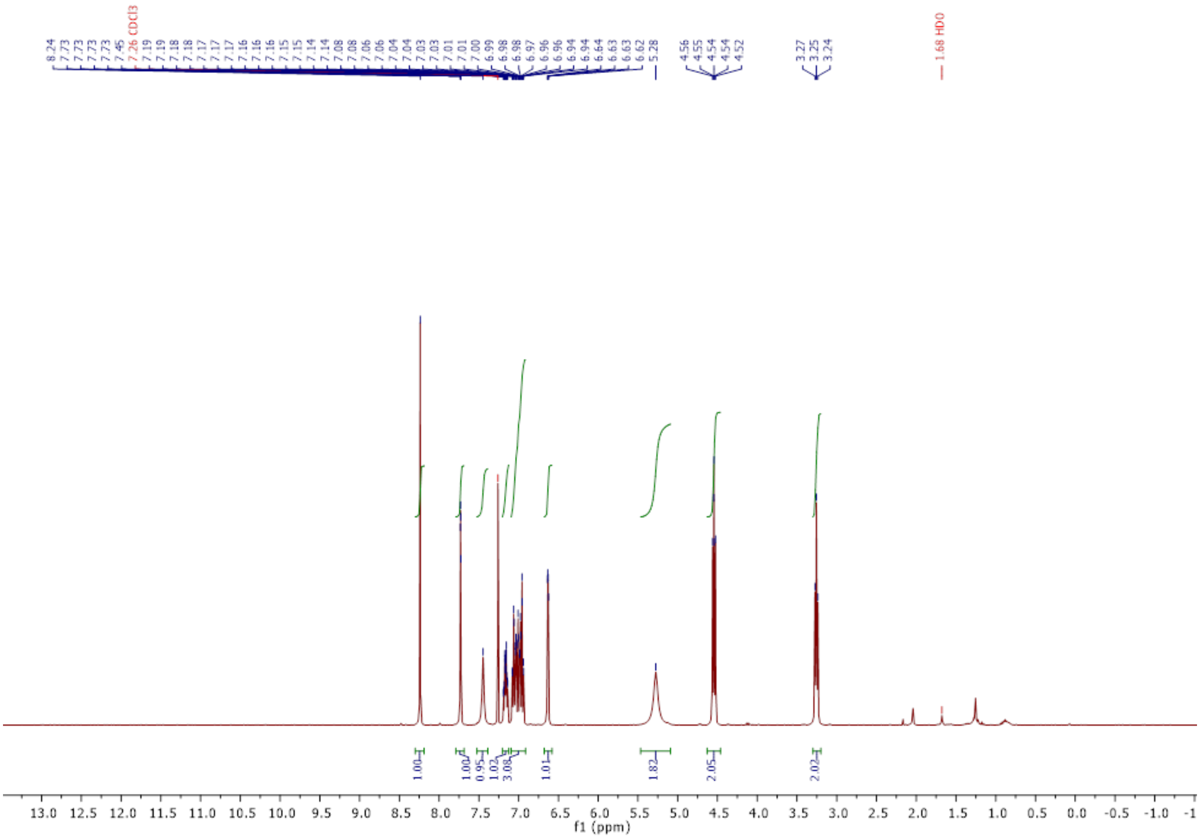

Figure S38: ^{1}H-NMR of **PPY19**.

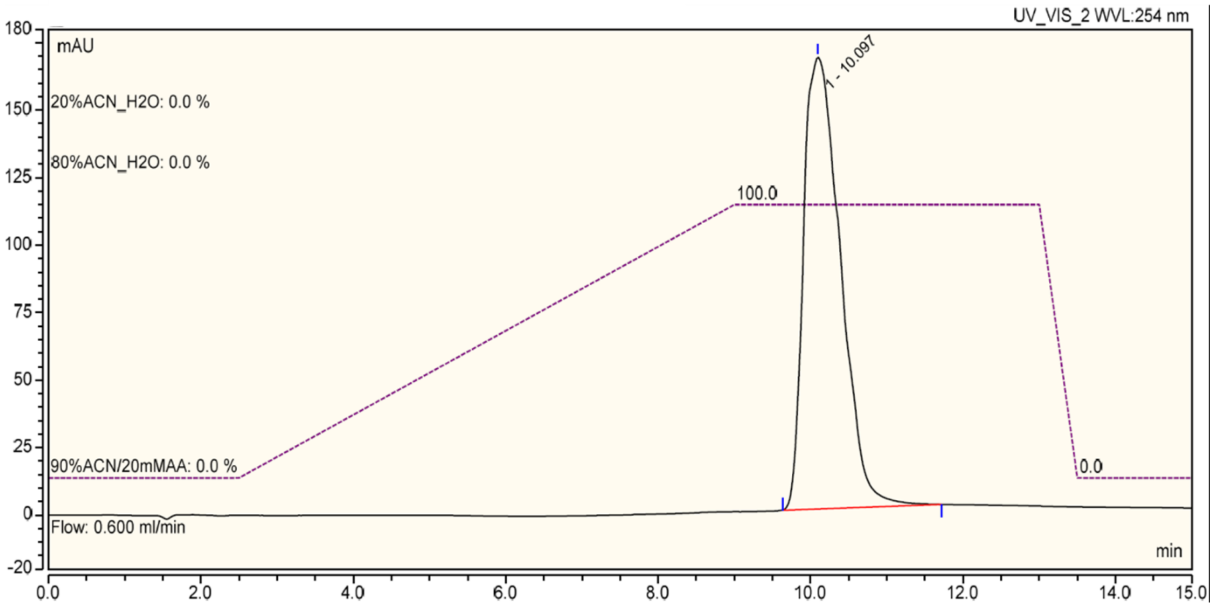

Figure S39: LC-MS chromatogram of **PPY19**.

(6-Amino-4-(furan-2-yl)-1*H*-pyrazolo[3,4-*d*]pyrimidin-1-yl)(2-fluorophenyl)-methanone (PPY20)

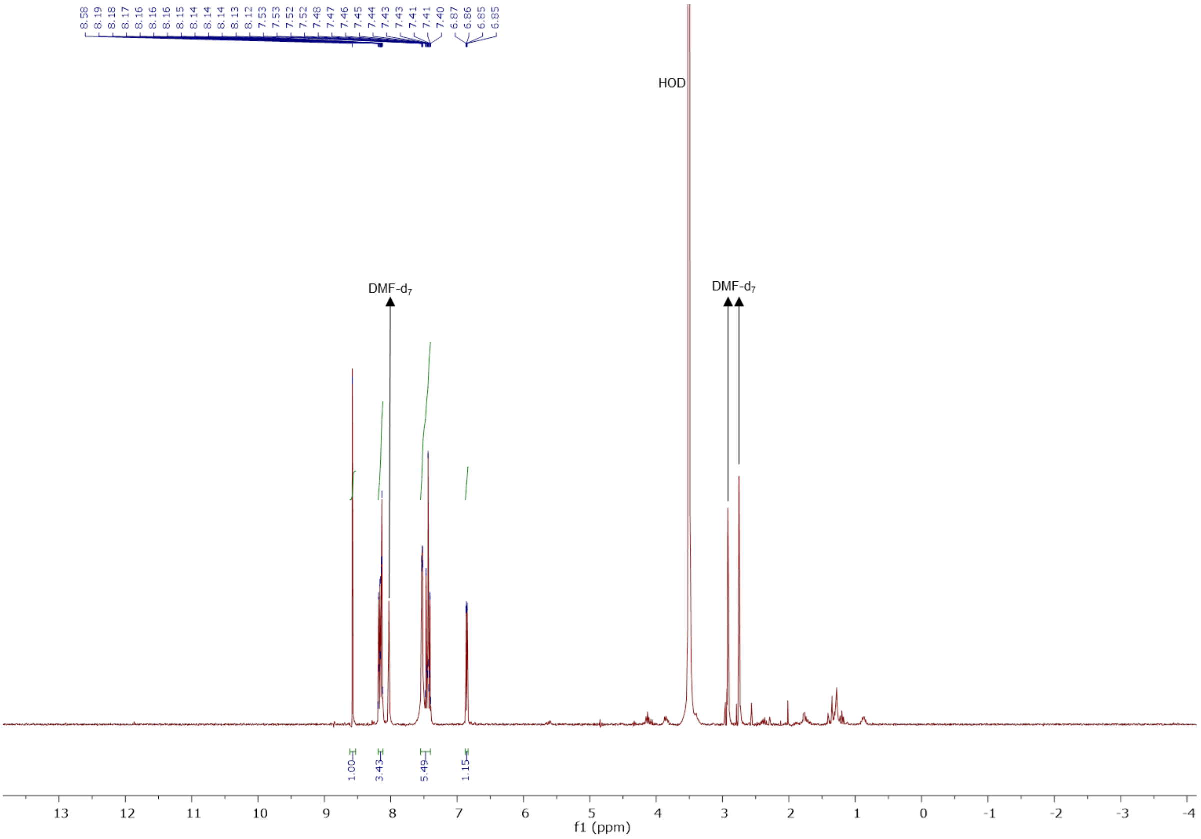

Figure S40: ^{1}H-NMR of **PPY20**.

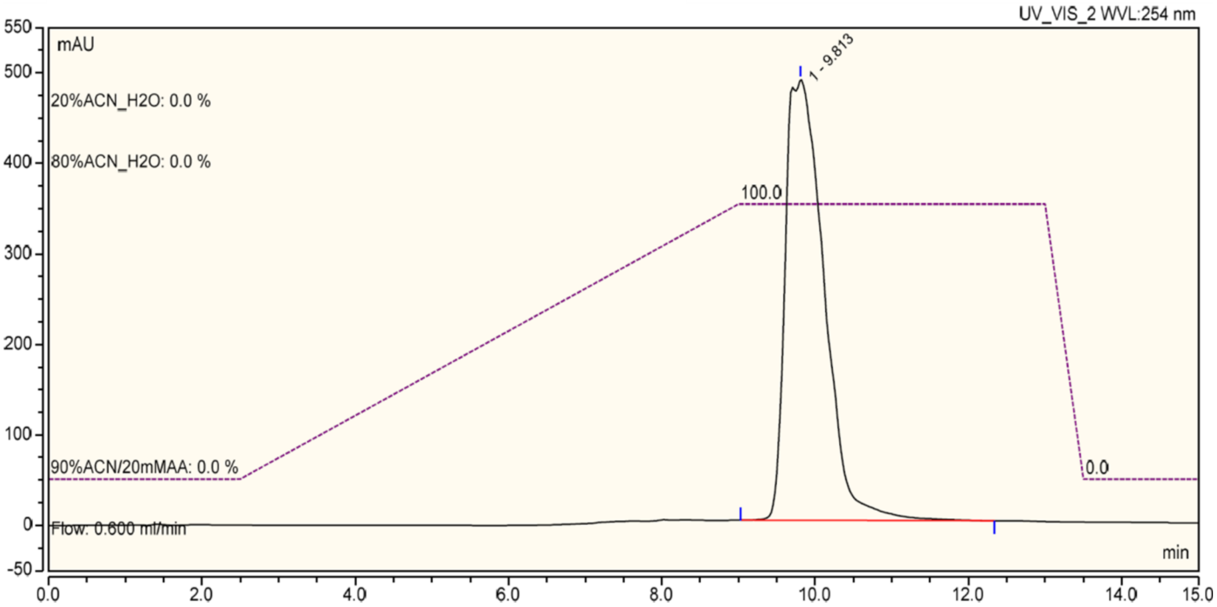

Figure S41: LC-MS chromatogram of **PPY20**.

(6-Amino-4-(furan-2-yl)-1H-pyrazolo[3,4-*d*]pyrimidin-1-yl)(2-fluorophenyl)-methanone (PPY21)

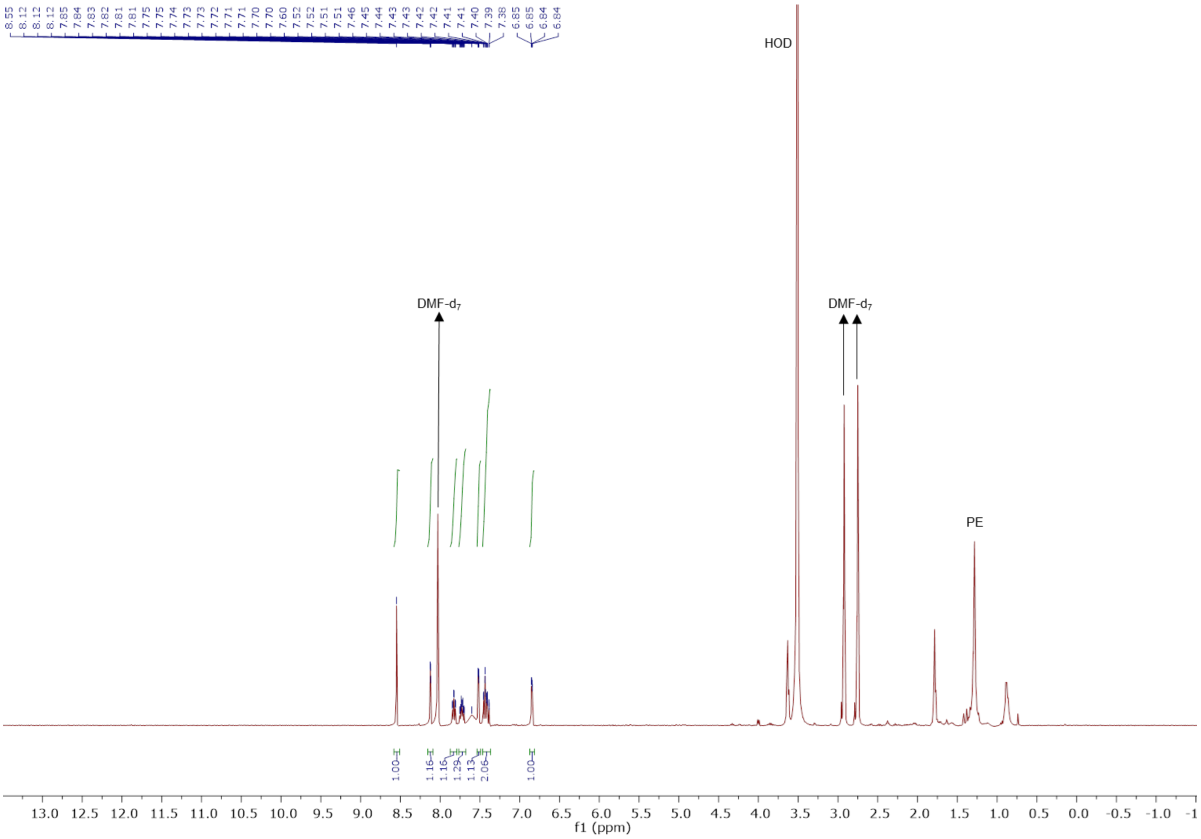

Figure S42: ^{1}H-NMR of **PPY21**.

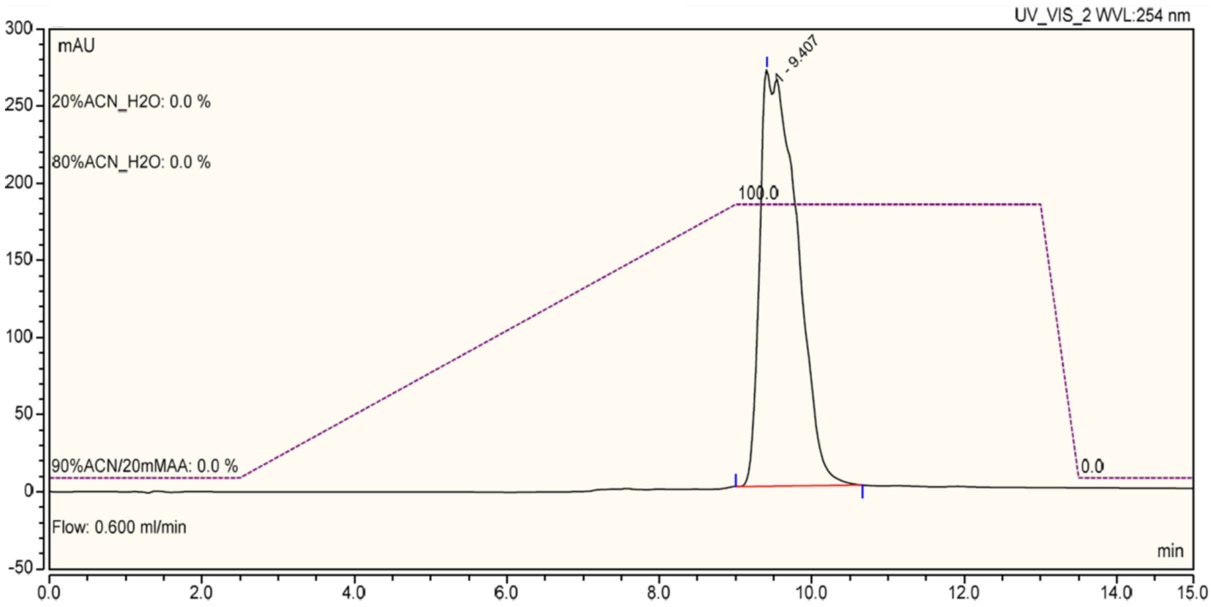

Figure S43: LC-MS chromatogram of **PPY21**.

1-Benzyl-4-(furan-2-yl)-1*H*-pyrazolo[3,4-*d*]pyrimidin-6-amine (PPY22)

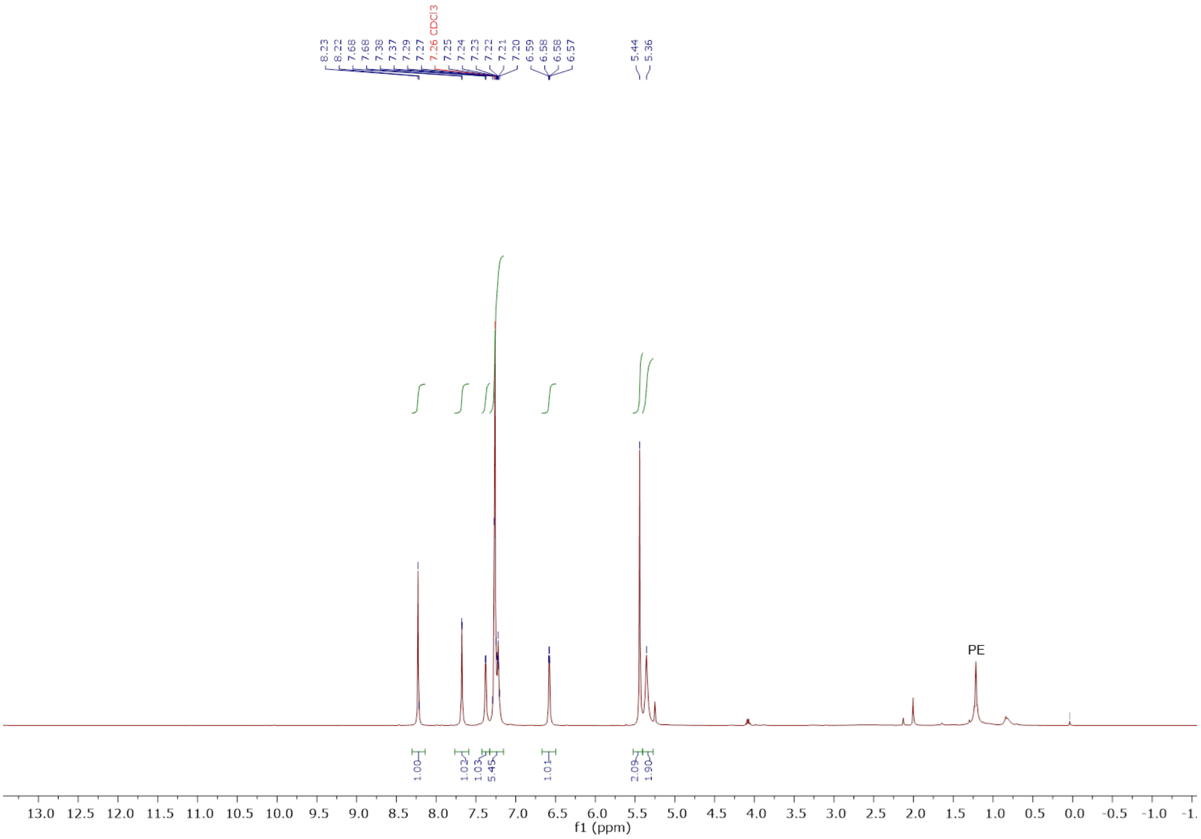

Figure S44: ^{1}H-NMR of **PPY22**.

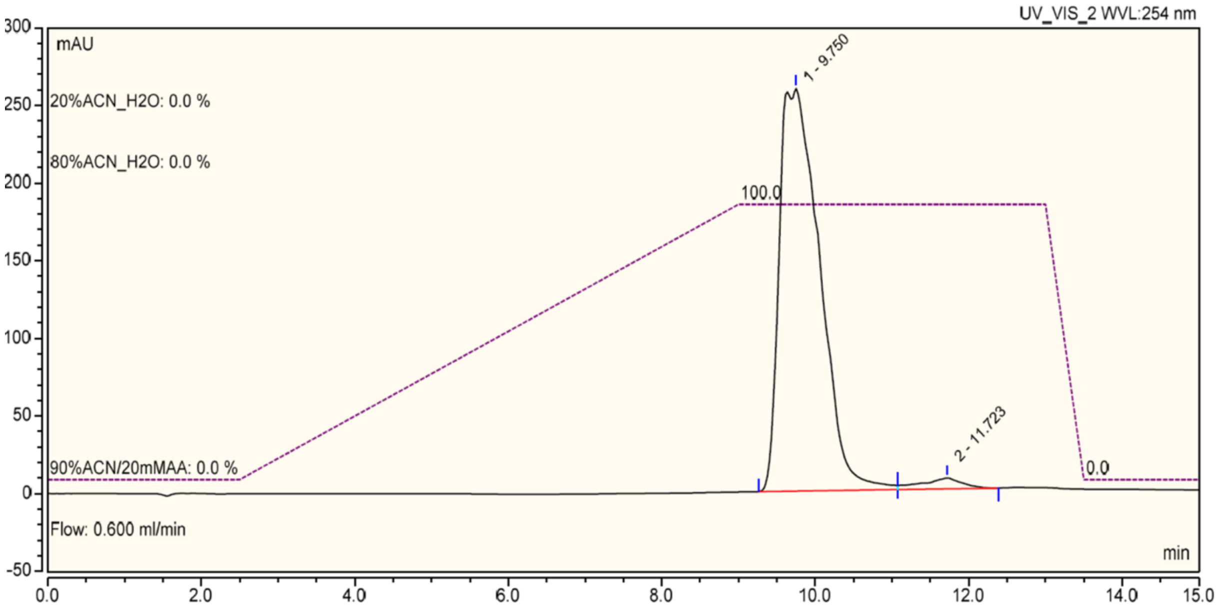

Figure S45: LC-MS chromatogram of **PPY22**.
